# Proteomics Analysis of Gastric Cancer Patients with Diabetes Mellitus

**DOI:** 10.3390/jcm10030407

**Published:** 2021-01-21

**Authors:** Hugo Osório, Cátia Silva, Marta Ferreira, Irene Gullo, Valdemar Máximo, Rita Barros, Fernando Mendonça, Carla Oliveira, Fátima Carneiro

**Affiliations:** 1 i3S—Instituto de Investigação e Inovação em Saúde, Universidade do Porto, 4200-135 Porto, Portugal; catias@i3s.up.pt (C.S.); martaf@ipatimup.pt (M.F.); irene.gullo12@gmail.com (I.G.); vmaximo@ipatimup.pt (V.M.); rbarros@ipatimup.pt (R.B.); carlaol@i3s.up.pt (C.O.); 2 Ipatimup—Institute of Molecular Pathology and Immunology of the University of Porto, University of Porto, 4200-135 Porto, Portugal; 3 Department of Pathology, Faculty of Medicine, University of Porto, 4200-319 Porto, Portugal; 4 Centro Hospitalar Universitário de São João, 4200-319 Porto, Portugal; fernandomiguel_92@hotmail.com

**Keywords:** gastric cancer (GC), diabetes mellitus (DM), proteomics, label free quantification (LFQ)

## Abstract

Proteomics is a powerful approach to study the molecular mechanisms of cancer. In this study, we aim to characterize the proteomic profile of gastric cancer (GC) in patients with diabetes mellitus (DM) type 2. Forty GC tissue samples including 19 cases from diabetic patients and 21 cases from individuals without diabetes (control group) were selected for the proteomics analysis. Gastric tissues were processed following the single-pot, solid-phase-enhanced sample preparation approach—SP3 and enzymatic digestion with trypsin. The resulting peptides were analyzed by LC-MS Liquid Chromatography—Mass Spectrometry (LC-MS). The comparison of protein expression levels between GC samples from diabetic and non-diabetic patients was performed by label-free quantification (LFQ). A total of 6599 protein groups were identified in the 40 samples. Thirty-seven proteins were differentially expressed among the two groups, with 16 upregulated and 21 downregulated in the diabetic cohort. Statistical overrepresentation tests were considered for different annotation sets including the Gene Ontology(GO), Kyoto Encyclopedia of Genes and Genomes (KEGG), Reactome, and Disease functional databases. Upregulated proteins in the GC samples from diabetic patients were particularly enriched in respiratory electron transport and alcohol metabolic biological processes, while downregulated proteins were associated with epithelial cancers, intestinal diseases, and cell–cell junction cellular components. Taken together, these results support the data already obtained by previous studies that associate diabetes with metabolic disorders and diabetes-associated diseases, such as Alzheimer’s and Parkinson’s, and also provide valuable insights into seven GC-associated protein targets, claudin-3, polymeric immunoglobulin receptor protein, cadherin-17, villin-1, transglutaminase-2, desmoglein-2, and mucin-13, which warrant further investigation.

## 1. Introduction

Gastric cancer (GC) is the fifth most frequent cancer and the third leading cause of cancer deaths worldwide [1]. The rate of incidence for GC is two-fold higher in men than in women and represents the most commonly diagnosed cancer, as well as the leading cause of cancer death in men from Eastern Asian countries [1].

Various risk factors are associated with this disease. One of the most common risk factors is infection by *Helicobacter pylori*, a World Health Organization (WHO) class I carcinogen that is thought to be responsible for the majority of GCs [2]. Behavioral factors such as smoking, low fruit and vegetable intake, and high salt intake increase the risk of developing GC [2,3]. A complex interplay of several risk factors, including genetic susceptibility, microbial community, and dietary habits, contributes to create a prone environment for gastric carcinogenesis [2,4].

Diabetes mellitus (DM) is a leading cause of death worldwide and is known to be associated with an increased risk for several cancer types, including hepatocellular, pancreatic and urothelial carcinoma. A possible relationship between DM and GC has been discussed for several years due to their common characteristics, including hyperinsulinemia, hyperglycemia, and inflammation [5]. It is estimated that in 2030, the DM population will be five times higher than that in 2000, thus substantially increasing the risk of cancer and related mortality worldwide [6]. Importantly, the above-mentioned risk factors for GC are also associated with an increased risk of DM [7].

However, the relation between GC and DM has not been completely elucidated. In 2011, Lin et al. noted hyperglycemia as a possible risk factor for the imbalance of energy/metabolism and impairment of the immune system as a cause of GC [8]. In this study, the authors observed an increased risk of GC in diabetic patients, regardless of their Body Mass Index. This theory was also confirmed in a meta-analysis performed by Cheung et al., in which type 2 DM was linked to gastric carcinogenesis in patients treated for *Helicobacter pylori* infection [9]. More recently, Mansory et al. performed a meta-analysis of case-control studies and observed a positive association between *Helicobacter pylori* infection and DM [10]. However, a review article by Tseng et al. published in 2014 concluded that the previous publications relating DM to a higher risk of GC had several limitations [11]. More recently, Zheng J et al. studied the relationship between prediabetes or diabetes and GC in a cohort including more than 110,000 participants with a long follow-up. In this Swedish cohort study, the authors did not find a clear association between the two diseases [12].

Several proteomics approaches have been developed to study GC in recent years. In 2019, Rostami-Nejad et al. reviewed 65 proteomics studies focusing on GC [13]. The authors highlighted the importance of heat shock proteins, metabolic proteins, and galectins, among other proteins, which may play a major role in gastric carcinogenesis. In a recent study combining transcriptomics and proteomic data with the objective of understanding the relation between DM and colon cancer, several signaling processes were found to be overrepresented in normal diabetic colon mucosa adjacent to malignant tissues that may be related with carcinogenesis in the setting of DM [14].

To the best of our knowledge, no proteomics studies have addressed GC patients in the context of type 2 DM.

In the current study, we performed a comprehensive proteomics approach on GC samples from 40 patients aiming to elucidate the possible links between DM and GC.

## 2. Experimental Section

### 2.1. Sample Selection

The study design was approved by the Ethical Committee of Centro Hospitalar Universitário de São João at 16 March 2017 under the Project entitled “Diabetes & obesity at the crossroads between Oncological and Cardiovascular diseases—a system analysis NETwork towards precision medicine (DOCnet)”. Forty samples of GC from 19 individuals with DM and 21 individuals without DM (controls) were processed for proteomic analysis. A diagnosis of diabetes mellitus was considered when at least 1 of the following criteria was met: (1) DM clearly listed in the clinical records; (2) the presence of analytical studies complying with the DM diagnostic criteria of the 2020 American Diabetes Association guidelines; and/or (3) the patient taking antidiabetic medication. The clinicopathological features of the 40 patients were collected from the clinical records and from the files of the Department of Pathology. To avoid confounding results and selection biases, the selection of DM and non-DM patients was performed rigorously by creating two groups of patients with an equivalent male:female ratio, median age of diagnosis, tumor stage, and histological type.

### 2.2. Protein Extraction

Frozen GC samples in Optimal Cutting Fluid (OCT) from each patient were independently processed in 2 mL tubes containing lysing matrix A (MP Biomedicals, Irvine, CA, USA) and a lysis buffer (100 mM Tris-HCl pH 8.5, 1% sodium deoxycholate (SDC), 10 mM tris (2-carboxyethyl) phosphine (TCEP), 40 mM chloroacetamide (CAA), and protease inhibitors. Protein homogenization was performed using FastPrep-24 equipment (MP Biomedicals) at 6.0 m/s in 3 cycles of 30 s each with intervals of 5 min on ice. Then, the protein extracts were centrifuged for 5 min at 13,400 *r.p.m.* using a benchtop centrifuge and transferred into 1.5 mL low protein binding tubes. Further, the extracts were incubated for 10 min at 95 °C at 1000 *r.p.m.* (Thermomixer, Eppendorf, Hamburg, Germany), sonicated (Bioruptor, Diagenode, Liège, Belgium) for ten cycles, 30 s on and 30 s off at 4 °C, followed by new centrifugation. The clarified lysate was then transferred into a new 1.5 mL vial, and the protein concentration was measured. One-hundred micrograms of protein from each sample were processed for proteomic analysis following the solid-phase-enhanced sample-preparation (SP3) protocol as described in [15]. Enzymatic digestion was performed with trypsin/LysC (2 micrograms) overnight at 37 °C at 1000 rpm. The resulting peptide concentration was measured by fluorescence.

### 2.3. Proteomics Data Acquisition

Protein identification and quantitation were performed by nanoLC-MS/MS. This equipment is composed of an Ultimate 3000 liquid chromatography system coupled to a Q-Exactive Hybrid Quadrupole-Orbitrap mass spectrometer (Thermo Scientific, Bremen, Germany). Five hundred nanograms of peptides of each sample were loaded onto a trapping cartridge (Acclaim PepMap C18 100 Å, 5 mm × 300 µm i.d., 160454, Thermo Scientific, Bremen, Germany) in a mobile phase of 2% ACN, 0.1% FA at 10 µL/min. After 3 min loading, the trap column was switched in-line to a 50 cm × 75 μm inner diameter EASY-Spray column (ES803, PepMap RSLC, C18, 2 μm, Thermo Scientific, Bremen, Germany) at 250 nL/min. Separation was achieved by mixing A: 0.1% FA and B: 80% ACN, 0.1% FA with the following gradient: 5 min (2.5% B to 10% B), 120 min (10% B to 30% B), 20 min (30% B to 50% B), 5 min (50% B to 99% B), and 10 min (hold 99% B). Subsequently, the column was equilibrated with 2.5% B for 17 min. Data acquisition was controlled by Xcalibur 4.0 and Tune 2.9 software (Thermo Scientific, Bremen, Germany).

The mass spectrometer was operated in the data-dependent (dd) positive acquisition mode alternating between a full scan (*m*/*z* 380-1580) and subsequent HCD MS/MS of the 10 most intense peaks from a full scan (normalized collision energy of 27%). The ESI spray voltage was 1.9 kV. The global settings were as follows: use lock masses best (*m*/*z* 445.12003), lock mass injection Full MS and chrom. peak width (FWHM) of 15 s. The full scan settings were as follows: 70 k resolution (*m*/*z* 200), AGC target 3 × 10^6^, maximum injection time 120 ms; dd settings: minimum AGC target 8 × 10^3^, intensity threshold 7.3 × 10^4^, charge exclusion: unassigned, 1, 8, >8, peptide match preferred, exclude isotopes on, and dynamic exclusion 45 s. The MS2 settings were as follows: microscans 1, resolution 35 k (*m*/*z* 200), AGC target 2 × 10^5^, maximum injection time 110 ms, isolation window 2.0 *m*/*z*, isolation offset 0.0 *m*/*z*, dynamic first mass, and spectrum data type profile.

### 2.4. Data Analysis

The raw data were processed using the Proteome Discoverer 2.5.0.400 software (Thermo Scientific, Bremen, Germany). Protein identification analysis was performed with the data available in the UniProt protein sequence database for the *Homo sapiens* Proteome 2020_05 with 75,069 entries and a common contaminant database from MaxQuant (version 1.6.2.6, Max Planck Institute of Biochemistry, Munich, Germany). Two protein search algorithms were considered: (i) the mass spectrum library search software MSPepSearch, with the NIST human HCD Spectrum Library (1,127,970 spectra and (ii) the Sequest HT tandem mass spectrometry peptide database search program. Both search nodes considered an ion mass tolerance of 10 ppm for precursor ions and 0.02 Da for fragment ions. The maximum allowed missing cleavage sites was set as 2. Cysteine carbamidomethylation was defined as constant modification. Methionine oxidation, asparagine and glutamine deamidation, peptide N-terminus Gln->pyro-Glut, protein N-terminus acetylation, and loss of methionine and Met-loss+Acetyl were defined as variable modifications. Peptide confidence was set to high. The Inferys rescoring node was considered for this analysis. The processing node Percolator was enabled with the following settings: maximum delta Cn 0.05; decoy database search target False Discovery Rate—FDR 1%; validation based on q-value. Protein-label-free quantitation was performed with the Minora feature detector node at the processing step. Precursor ion quantification was performing at the processing step with the following parameters: Peptides: unique plus razor; precursor abundance was based on intensity; normalization mode was based on the total peptide amount; the minimum amount of replicate files that a feature must be detected in to be used was set to 50% in the sample group; the pairwise protein ratio calculation and hypothesis test were based on a *t*-test (background based). The Feature Mapper node from the Proteome Discoverer software was used to create features from unique peptide-specific peaks within a small retention-time and mass range. This was achieved by applying a chromatographic retention time alignment with a maximum shift of 10 min and 10 ppm of mass tolerance allowed for mapping features from different sample files. For feature linking and mapping, the minimum DM vs. control signal to noise (S/N) threshold was set at 5.

### 2.5. Protein Functional Enrichment Analysis

For determination of differentially expressed proteins between the DM and control cases, the following filters were considered additionally: (1) the minimum number of samples that a protein must be detected to be used was set to 50% per experimental group; (2) the use of at least two unique peptides and the *p*-value adjusted using Benjamini–Hochberg correction for the FDR set to ≤0.05; (3) the DM/control considered ratio was set to ≥1.50 for the selection of upregulated proteins and to ≤0.667 for downregulated proteins; (4) at least 50% of samples (minimum 21 out of 40) with protein-related peptides sequenced by MS/MS. Volcano plot analysis was performed with the Proteome Discoverer software after applying the above described filters. The Principal Component Analysis—PCA—was carried out with the ClustVis software (https://biit.cs.ut.ee/clustvis/).

Protein functional enrichment analysis was performed with the WebGestalt (WEB-based Gene SeT AnaLysis Toolkit) [16]. The Over-Representation Analysis (ORA) was performed under the following conditions: minimum/maximum number of genes for a category 5/2000 and significance level *p* ≤ 0.05, with the *p*-values adjusted following the Benjamini–Hochberg methodology. The following parameters were considered for the Gene Set Enrichment Analysis (GSEA) analysis: minimum/maximum number of genes for a category, 5/2000; significance level *p* ≤ 0.05; 1000 permutations. The *t*-statistic value between the two groups was determined for all quantified proteins. For both ORA and GSEA approaches, the following Functional Databases were considered: Gene Ontology (BP—Biological Process, CC—Cellular Component and MF—Molecular Function), Pathway (KEGG—Kyoto Encyclopedia of Genes and Genomes, Panther, Reactome, Wikipathway, and Wikipathway cancer) and Disease (Disgenet, GLAD4U and OMIM).

## 3. Results

### 3.1. Clinical Data

**Figure 1:**
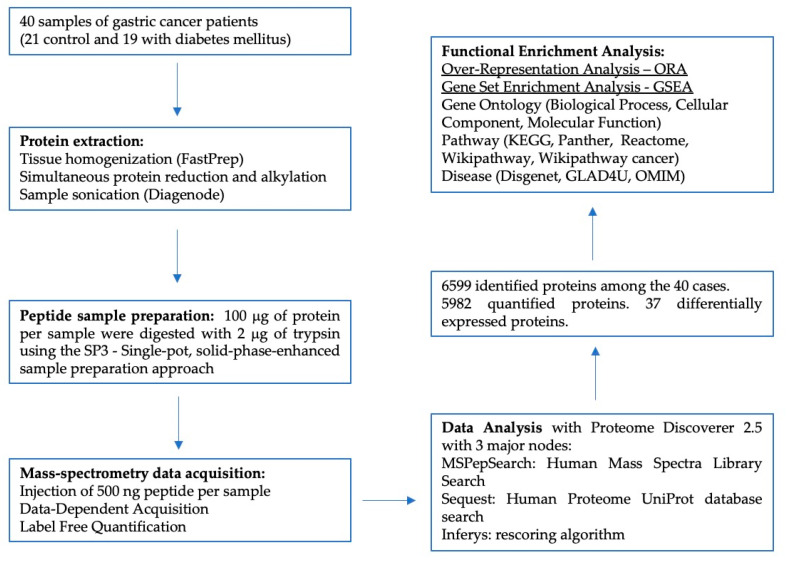
Schematic representation of the proteomics workflow for the analysis of 40 samples of Gastric Cancer (GC) patients with Diabetes Mellitus (DM) and controls without this condition.

The clinicopathological data from the 40 GC patients are listed in Table 1. There were no significant differences in the clinicopathological features of the two groups, including gender, median age of diagnosis, tumor stage, and histological type (*p* > 0.05). A schematic representation of the proteomics workflow performed in this study is depicted in Figure 1.

### 3.2. Proteomics Analysis

A total of 6625 protein groups were identified among the 40 GC samples analyzed. After the removal of common contaminants, the number of identified protein groups decreased to 6599. In total, 5982 protein groups were quantified (Supplementary File S1).

Relative quantification of the protein expression levels was performed using the LFQ—Label Free Quantification approach. A comparison of differentially expressed proteins between diabetes mellitus (DM) and the control (C) was performed (Table 2). Considering the diabetes/control ratio, 37 proteins were found to be differentially expressed in samples from patients with DM; 16 proteins were found to be upregulated and 21 downregulated.

**Figure 2:**
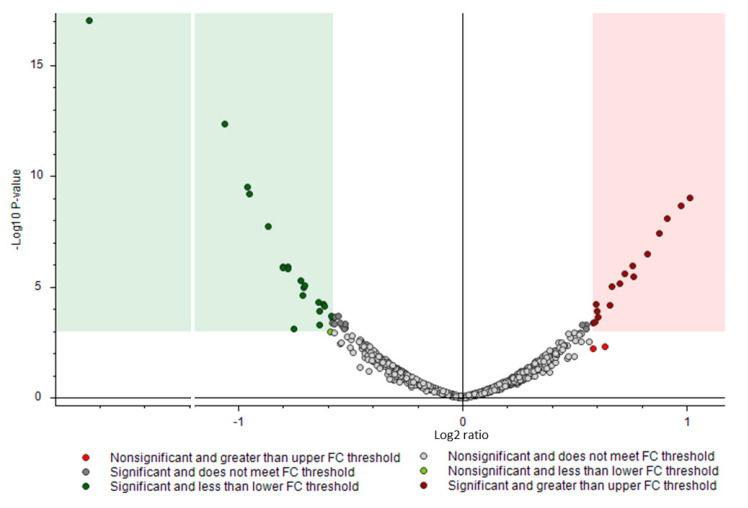
Volcano Plot highlighting the 37 differentially expressed proteins between DM and controls without this condition. The 16 upregulated and 21 downregulated proteins are highlighted in dark red and dark green, respectively. The figure was cropped between a -1.2 and -6.2 Log2 ratio to allow a clearer visualization of the data.

**Figure 3:**
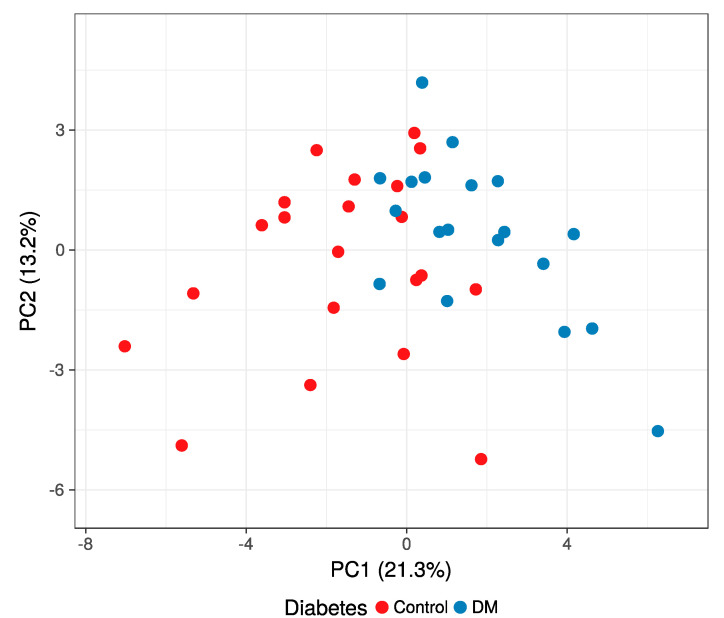
Principal Component Analysis—PCA—using the panel of 37 differentially expressed proteins from the 19 samples of DM patients (blue colored) and 21 controls without this condition (red colored).

The differences between the two groups can be observed at the volcano plot depicted in Figure 2. A Principal Component Analysis—PCA—was performed to compare the similarities between the following clinical factors: DM; Lauren classification; and pT, pN, and M categories (Figure 3 and Supplementary File S2). The filter criteria 1, 2, and 4, described in Section 2.4, were applied for the protein selection. No relevant differences between the assayed clinical pathological features were found. To further explore the data, we additionally applied criterion 3 considering the differentially expressed proteins between DM and C gastric cancer patients and performed a PCA analysis (Figure 3). Most of the samples of DM were separated from the C group.

### 3.3. Functional Enrichment Analysis

Over-Representation Analysis (ORA) was performed for the differentially expressed proteins observed between the DM and control sample groups with the functional enrichment analysis web tool WebGestalt [16] (Table 3). All 6599 identified protein groups, excluding common contaminants, were added as the “Reference Gene List” in the software. The information associated with the proteins for each of the analysed functional categories is presented in Table 3 and Supplementary File S3. The interactive information from the WebGestalt software for the ORA is available in Supplementary File S4. For the upregulated proteins, in the Cellular Component category of Gene Ontology, the term “Extracellular region” provided statistically significant results with 11 associated proteins and an enrichment ratio of 2.76. For downregulated protein expression, the terms “Intestinal Diseases” and “Epithelial Cancers” from the Disease GLAD4U enrichment category were found, with enrichment ratios of 10.68 and 9.64, respectively. Both terms relate to a mutual set of five proteins: transglutaminase 2, polymeric immunoglobulin receptor, claudin 3, villin 1, and mucin 13, with one specific protein for each protein: The protein cadherin 17 is associated with “Intestinal Diseases”, and the protein desmoglein-2 is associated with the “Epithelial Cancers” category. All seven proteins have a major role in GC that is described in Section 4.

**Figure 4:**
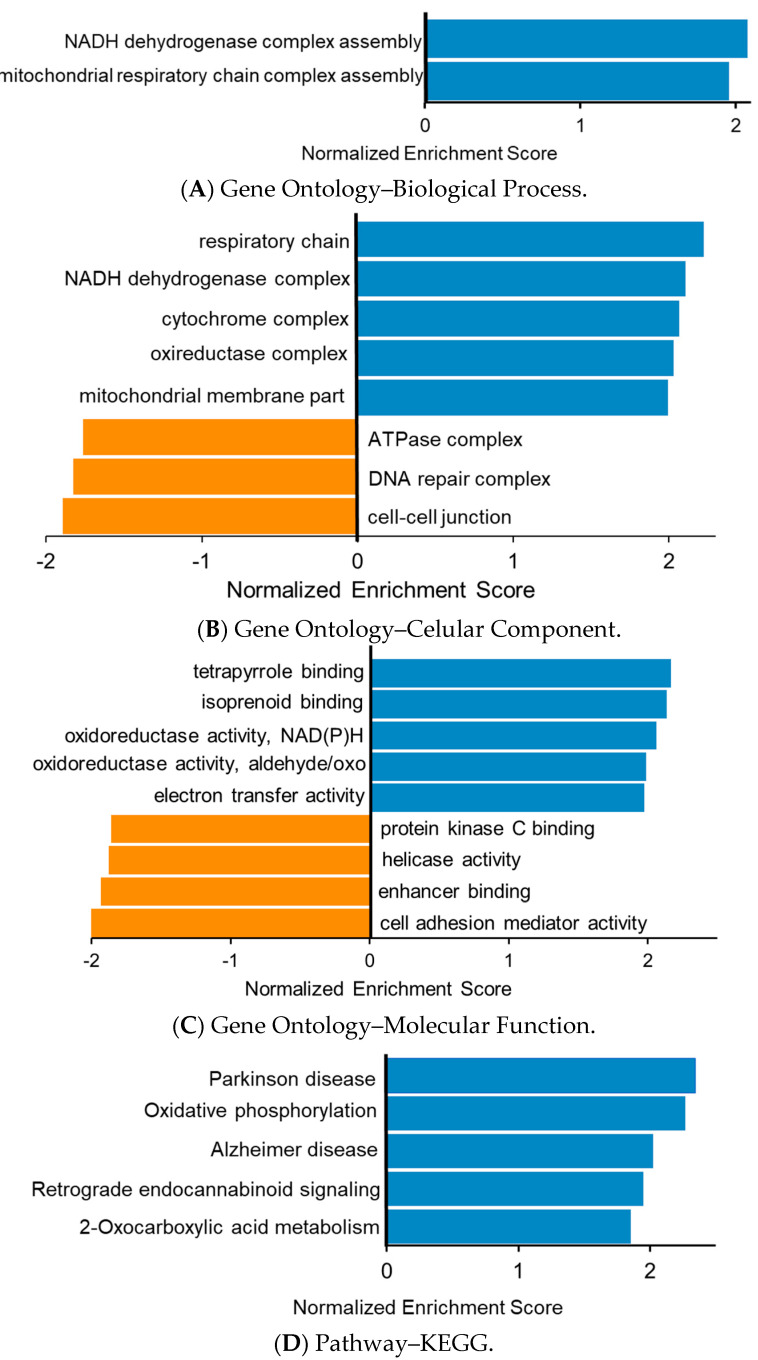

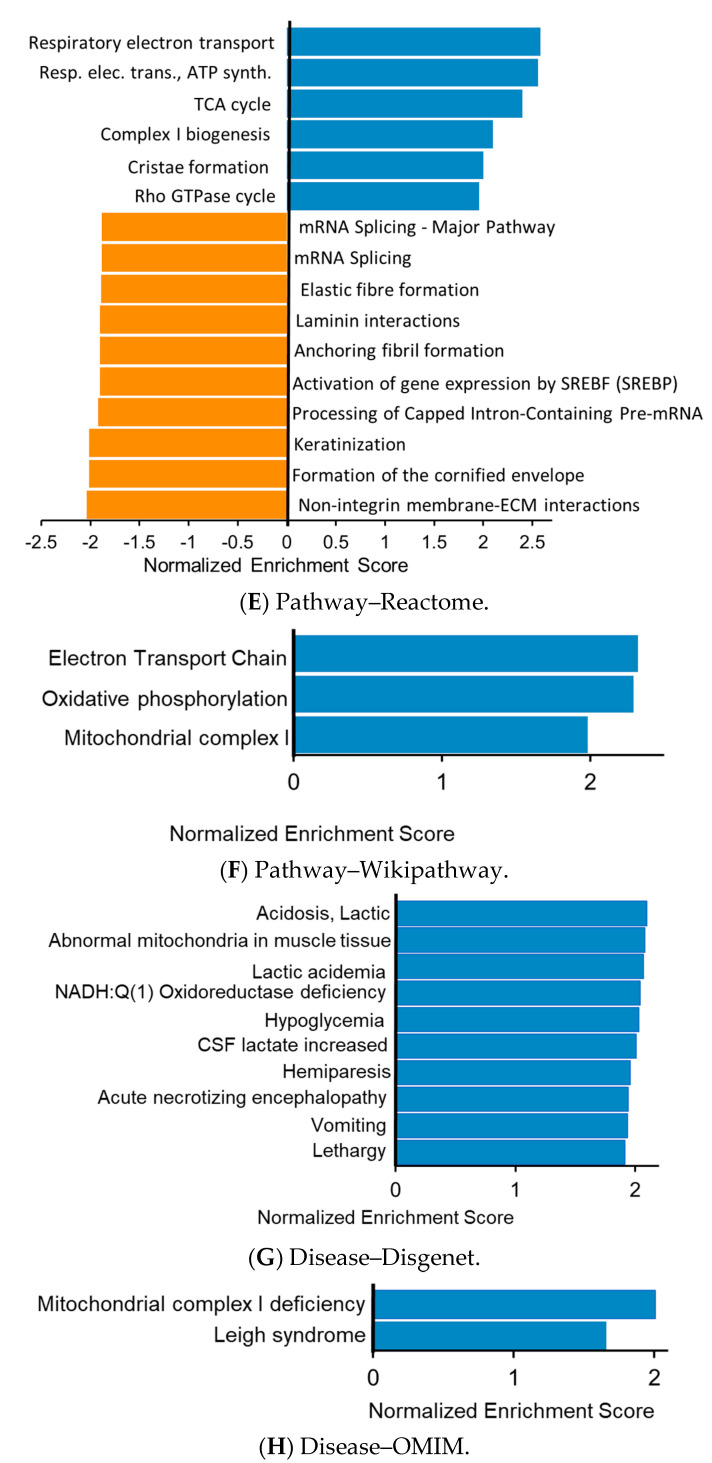
Functional enrichment analysis of the 5982 quantified proteins in the diabetes mellitus condition using WebGestalt (WEB-based Gene SeT AnaLysis Toolkit) with the Gene Set Enrichment Analysis (GSEA) enrichment method. A *p*-value ≤ 0.05 was considered with adjustment using the Benjamini–Hochberg approach. The Gene Ontology functional database was considered for the BP (**A**), CC (**B**), and MF (**C**) categories. The following Pathways were used: KEGG (**D**), Reactome (**E**), and Wikipathway (**F**). Two Disease functional databases were also used: Disgenet (**G**) and OMIM (**H**). The upregulated pathways are depicted in blue, and the downregulated proteins are displayed in orange. The proteins associated with each category can be found in Supplementary Table S3.

To extract further biological and clinical information on the obtained data, we also performed a GSEA approach for the diabetes/control group comparison (Figure 4). This approach focuses on groups of proteins that share common biological functions, pathways, or diseases. Like ORA, only the statistically significant results are shown for a *p*-value ≤ 0.05. The information for the proteins associated with each category and the associated enrichment plots are provided in Supplementary File S5. The interactive information with the WebGestalt software for GSEA is provided in Supplementary File S6. For the GO functional database, we observed upregulation of the protein sets, with a high Normalized Enriched Score (NES) indicating mitochondrial respiratory chain and oxidoreductase activity. Also relevant are the decreases in three sets related to the cellular component category: “ATPase complex”, “DNA repair complex”, and “cell–cell junction”. The pathway analysis involved this information with some new data, such as increases in the “Rho GTPase cycle” and decreases in “mRNA splicing” gene sets. In the disease functional databases Disgenet and OMIM, we observed an increase in acid lactic acidosis and mitochondrial deficiency. Importantly, the Pathway–KEGG method also found high NES values for Parkinson’s and Alzheimer’s diseases.

## 4. Discussion

The intricate relationship between diabetes and cancer remains an important challenge to be addressed. In this study, we performed a comprehensive mass spectrometry-based proteomics study that included 40 samples of GC patients, including 19 with diabetes and 21 control cases. With more than 6000 proteins characterized, we performed a differential protein expression analysis to compare GC samples from the diabetic and non-diabetic patients. In addition, we performed a functional enrichment analysis, including Over-Representation Analysis (ORA) and Gene Set Enrichment Analysis (GSEA).

One of the most relevant pathways observed by the GSEA for the Reactome database was the “Respiratory electron transport”, which had a normalized enrichment score of 2.58. Forty-six out of 78 proteins from this pathway were enriched in diabetic patients. Among these proteins, different subunits of NAD:ubiquinone oxidoreductase (A1, S6, AB1, B3, and A8, among others) and NAD dehydrogenase subunits 3, 4, and 5 (complex I) were observed (Supplementary File S5 and File S6). Other similarly related pathways were also found to be enriched, including “Respiratory electron transport, ATP synthesis by chemiosmotic coupling, and heat production by uncoupling proteins”, “The citric acid (TCA) cycle and respiratory electron transport”, and “Complex I biogenesis”, highlighting the role of mitochondrial uncoupling in DM etiopathogenesis [17]. This study also found upregulation in proteins related to RhoGTPase activation, a pathway that was previously described [18]. We also found a negative association with ATPase complex proteins, presenting a normalized enrichment score of −1.76. This negative correlation was already previously described [19]. Our results agree with several studies highlighting mitochondrial impairment in diabetes [20,21,22]. Diabetes-induced mitochondrial dysfunction is characterized by a shift in energetic metabolism leading to the synthesis of ATP due to the oxidation of fatty acids and not the metabolism of carbohydrates. In addition, a possible excessive accumulation of Ca^2+^ in the mitochondrial matrix membrane will lead to a decrease in the mitochondrial membrane potential and impairment of ATP synthesis. Another consequence is an increase of Reactive Oxygen Species (ROS) production. These and other consequences of DM and mitochondrial dysfunction were recently reviewed [23].

Meaningful associations with well-documented diabetes-associated diseases were also observed. We found 59 proteins associated with Parkinson’s disease in KEGG pathway enrichment. Sergi et al. [24] recently reviewed the correlation between this condition and diabetes. This GSEA analysis also found enrichment in 54 proteins of Alzheimer’s disease, another well-described diabetes-related form of dementia [25].

In the ORA, we found two downregulated disease pathways, “Intestinal Disease” and “Epithelial Cancers”, in DM patients with GC, with 10.7 and 9.6 enrichment ratios, respectively. This result has particular relevance since the five shared proteins together with one protein specifically associated with each of the gene sets—seven in total (Table 3)—were already reported as being associated with GC and gastric pre-malignant lesions, as follows: (1) the down-regulation of claudin-3, a tight junction protein, was associated with GC progression [26]; (2) the polymeric immunoglobulin receptor protein, a major player of the mucosal immune system that mediates epithelial transcytosis of immunoglobulins, was observed to be higher in gastric intestinal metaplasia compared to in normal tissues and cancer [27]; (3) cadherin-17, a transmembrane glycoprotein and member of the cadherin family with an important role in tumorigenesis, was found to be over-expressed in GC [28]; (4) villin-1, an actin-binding protein, was observed to be significantly lower in GC compared to non-neoplastic mucosa [29]; (5) GC patients whose tumors featured high expressions of Transglutaminase-2 (protein-glutamine gamma-glutamyltransferase 2), an acyltransferase enzyme that also serves as a G protein for several transmembrane receptors and acts as a co-receptor for integrin β1 and β3 integrins [30], were observed to have a worse prognosis than those with a low expression of this enzyme [31]; (6) mucin-13, a glycoprotein mainly expressed in the digestive tract, was found to be overexpressed in intestinal-type GC [32]; (7) desmoglein-2, a major component of the desmosomes, is a cell adhesion molecule with functional similarities to E-cadherin and was described to be abnormally expressed in GC [33]. Taken together, these seven downregulated proteins in DM patients form a panel of candidate targets to be addressed in future studies to better understand the interactions between these diseases.

In addition, it is noteworthy that some of the deregulated pathways are associated with cancer. We observed an important negative correlation with the DNA repair complex [34], featuring six proteins integrating with this group, as well as a decrease in the cell–cell junction, including 58 proteins with a pivotal role in cancer progression [35]. Our results add new data to a previous study [12] in which a clear association between GC and DM was not found.

One limitation of this study is the lack of validation for some of the selected targets using a complementary approach, such as Western Blot or Immunofluorescence. The PCA analysis also showed only a limited correlation between the studied clinical settings. In a future study, further information, such as transcriptomics/genomics data and additional specific disease settings of the patients, will be included. However, it is noteworthy that both the GSEA and ORA of differently expressed proteins provided here agree, in great proportion, with previous observations in different disease settings, thus validating the results of this study.

To summarize, in this study, we performed a comprehensive proteomics analysis on a total of 40 GC patients to understand the relevance of DM conditions in this disease. The developed pipeline included protein extraction; mass spectrometry data acquisition; analysis of differentially expressed proteins; and functional analysis of gene ontology, pathway, and disease categories. Upregulated proteins in the GC samples from diabetic patients showed a strong fold-enrichment associated with respiratory electron transport and alcohol metabolic biological processes, while downregulated proteins were associated with epithelial cancers, intestinal diseases, and cell–cell junction cellular components. We also observed the enrichment of proteins previously described in well-documented diabetes-associated diseases and detected seven proteins downregulated in DM patients with potential clinical relevance, meriting investigation in future studies to further understand the correlation between DM and GC.

## Figures and Tables

**Table 1:** Clinicopathological data from GC patients with DM and without DM.

	Non-DM Patients	DM Patients
Patients (N)	21	19
Gender (male: female)	7:14	7:12
Median age (range)	75 (57–85)	75 (58–87)
Laurén classification (*n*—%)		
Intestinal	13 (61.9)	10 (52.6)
Diffuse	1 (4.8)	1 (5.3)
Mixed	7 (33.3)	6 (31.6)
Indeterminate	0 (0)	2 (10.5)
pT category (N—%)		
pT1a, pT1b	1 (4.8)	2 (10.5)
pT2	2 (9.5)	1 (5.3)
pT3	13 (61.9)	12 (63.1)
pT4a, pT4b	5 (23.8)	4 (21.1)
pN category (N—%)		
pN0	6 (28.6)	9 (47.4)
pN ≥ 1	15 (71.4)	10 (52.6)
M category (N—%)		
M0	20 (95)	16 (84.2)
(p)M1	1 (4.8)	3 (15.8)

GC: Gastric Cancer; DM: Diabetes Mellitus.

**Table 2:** List of Differentially Expressed Proteins (DEP) between Diabetes mellitus (DM) and Control (C) gastric cancer patients. A total of 37 proteins were detected following the criteria defined in Section 2.4. In total, 16 proteins were over-expressed and 21 under-expressed in DM patients. Further details can be found in Supplementary File S1.

Protein Name	Accession No.	Gene Symbol	Fold-Change (DM/C)	Unique Peptides
Immunoglobulin lambda variable 3–27	P01718	IGLV3-27	2.023	4
Desmin	P17661	DES	1.968	42
Gastricsin	P20142	PGC	1.885	3
Protein S100-P	P25815	S100P	1.837	5
Galectin-10	Q05315	CLC	1.771	7
Aldehyde dehydrogenase, dimeric NADP-preferring	P30838	ALDH3A1	1.701	21
Caveolae-associated protein 3	E9PIE3	CAVIN3	1.691	8
Gastrokine-2	Q86XP6	GKN2	1.654	6
Eosinophil peroxidase	P11678	EPX	1.629	15
Marginal zone B- and B1-cell-specific protein	Q8WU39	MZB1	1.586	11
Annexin A10	Q9UJ72	ANXA10	1.578	16
Protein S100-A8	P05109	S100A8	1.523	12
Immunoglobulin kappa variable 4-1	P06312	IGKV4-1	1.517	2
Carboxymethylenebutenolidase homolog	Q96DG6	CMBL	1.510	10
High mobility group protein HMG-I/HMG-Y	P17096	HMGA1	1.507	4
Neutrophil collagenase	P22894	MMP8	1.500	11
Protein-glutamine gamma-glutamyltransferase 2	P21980	TGM2	0.667	32
Type-1 angiotensin II receptor-associated protein	Q6RW13	AGTRAP	0.664	2
Glycerol-3-phosphate dehydrogenase [NAD(+)], cytoplasm.	P21695	GPD1	0.654	14
Transcription factor BTF3	P20290	BTF3	0.651	7
Monoglyceride lipase	Q99685	MGLL	0.643	7
Metal cation symporter ZIP14	Q15043	SLC39A14	0.642	4
Desmoglein-2	Q14126	DSG2	0.642	28
Polymeric immunoglobulin receptor	P01833	PIGR	0.615	54
Fatty acid-binding protein, liver	P07148	FABP1	0.613	9
Claudin-3	O15551	CLDN3	0.610	4
Myosin-14	Q7Z406	MYH14	0.606	54
Complement C1q subcomponent subunit A	P02745	C1QA	0.595	3
Cellular nucleic acid-binding protein	P62633	CNBP	0.584	5
Villin-1	P09327	VIL1	0.584	35
Protein POF1B	Q8WVV4	POF1B	0.575	19
CD59 glycoprotein (Fragment)	A0A2U3TZL5	CD59	0.574	4
Estradiol 17-beta-dehydrogenase 2	P37059	HSD17B2	0.549	12
Cadherin-17	Q12864	CDH17	0.518	21
Mucin-13	Q9H3R2	MUC13	0.515	6
Carcinoembryonic antigen-related cell adhesion molecule 5	A0A024R0K5	CEACAM5	0.480	8
YTH domain-containing family protein 1	Q9BYJ9	YTHDF1	0.010	2

**Table 3:** Functional enrichment analysis of the 37 differentially expressed proteins (DEP) (+, upregulated; −, downregulated) in DM conditions using WebGestalt (WEB-based Gene SeT AnaLysis Toolkit) with the Over-Representation Analysis (ORA) enrichment method.

DEP	Functional Database	GENE Set	Description	Protein Name
+	GO—CC	GO:0005576	Extracellular region	S100 calcium binding protein A8 eosinophil peroxidase desmin progastricsin matrix metallopeptidase 8 S100 calcium binding protein P aldehyde dehydrogenase 3 family member A1 Galectin-10 gastrokine 2 marginal zone B and B1 cell specific protein carboxymethylenebutenolidase homolog
−	Disease—GLAD4U	PA444632	Intestinal Diseases	transglutaminase 2 polymeric immunoglobulin receptor claudin 3 villin 1 cadherin 17 mucin 13, cell surface associated
−	Disease—GLAD4U	PA447242	Epithelial Cancers	transglutaminase 2 desmoglein 2 polymeric immunoglobulin receptor claudin 3 villin 1 mucin 13, cell surface associated

A *p*-value ≤ 0.05 was considered, with all 6599 proteins identified, excluding common contaminants, used as a reference list. The *p* values were adjusted using the Benjamini–Hochberg approach. The Gene Ontology (GO) functional database was considered for Biological Process (BP), Cellular Component (CC), and Molecular Function (MF) categories. The following Pathway databases were considered: Kyoto Encyclopedia of Genes and Genomes (KEGG), Panther, Reactome, Wikipathway, and Wikipathway cancer. Three Disease functional databases were also considered: Disgenet, GLAD4U, and OMIM.

## Data Availability

The mass spectrometry proteomics data were deposited to the ProteomeXchange Consortium via the Proteomics Identification Database (PRIDE) [36], partner repository with the dataset identifier PXD022915, and project DOI: 10.6019/PXD022915.

## Supplementary Materials

The following are available online at https://www.mdpi.com/2077-0383/10/3/407/s1, Supplementary File S1: Protein Identification and differential DM/control protein expression lists; Supplementary File S2: Principal Component Analysis (PCA) of DM, Lauren classification, pT, pN, and M categories; Supplementary File S3: Functional Enrichment Analysis of differentially expressed proteins—Over-Representation Analysis (ORA); Supplementary File S4: WebGestalt information of the ORA; Supplementary File S5: Functional Enrichment Analysis of differentially expressed proteins—Gene Set Enrichment Analysis (GSEA); Supplementary File S6: WebGestalt information of the GSEA.

## Abbreviations

GC	Gastric Cancer
DM	Diabetes mellitus
MS	Mass spectrometry
ORA	Over-Representation Analysis
GSEA	Gene Set Enrichment Analysis
