# Supplementary material for: Proteomics Analysis of Gastric Cancer Patients with Diabetes Mellitus
Source: J Clin Med. 2021 Jan 21;10(3):407. doi: 10.3390/jcm10030407 (PMC7866049; doi:10.3390/jcm10030407)
Supplement: Supplementary file 1 [file jcm-10-00407-s001.zip › sup/Supplementary_File_2.docx]

**Supplementary File 2**

2A

2B

2C

2D

2E

**Scheme 2.** Principal Component Analysis – PCA considering the clinical conditions described in Table 1: Diabetes – DM and control samples (**2A**), Laurén Classification – Diffuse, Intermediate, Intestinal and Mixed samples (**2B**), pT category -T1, T2, T3 and T4 (**2C**), pN category – pN0 and pN>=1 (**2D**) and M category – M0 and M1 (**2E**).
