# Supplementary figures and images for: Proteomics Analysis of Gastric Cancer Patients with Diabetes Mellitus
Source: J Clin Med. 2021 Jan 21;10(3):407. doi: 10.3390/jcm10030407 (PMC7866049; doi:10.3390/jcm10030407)

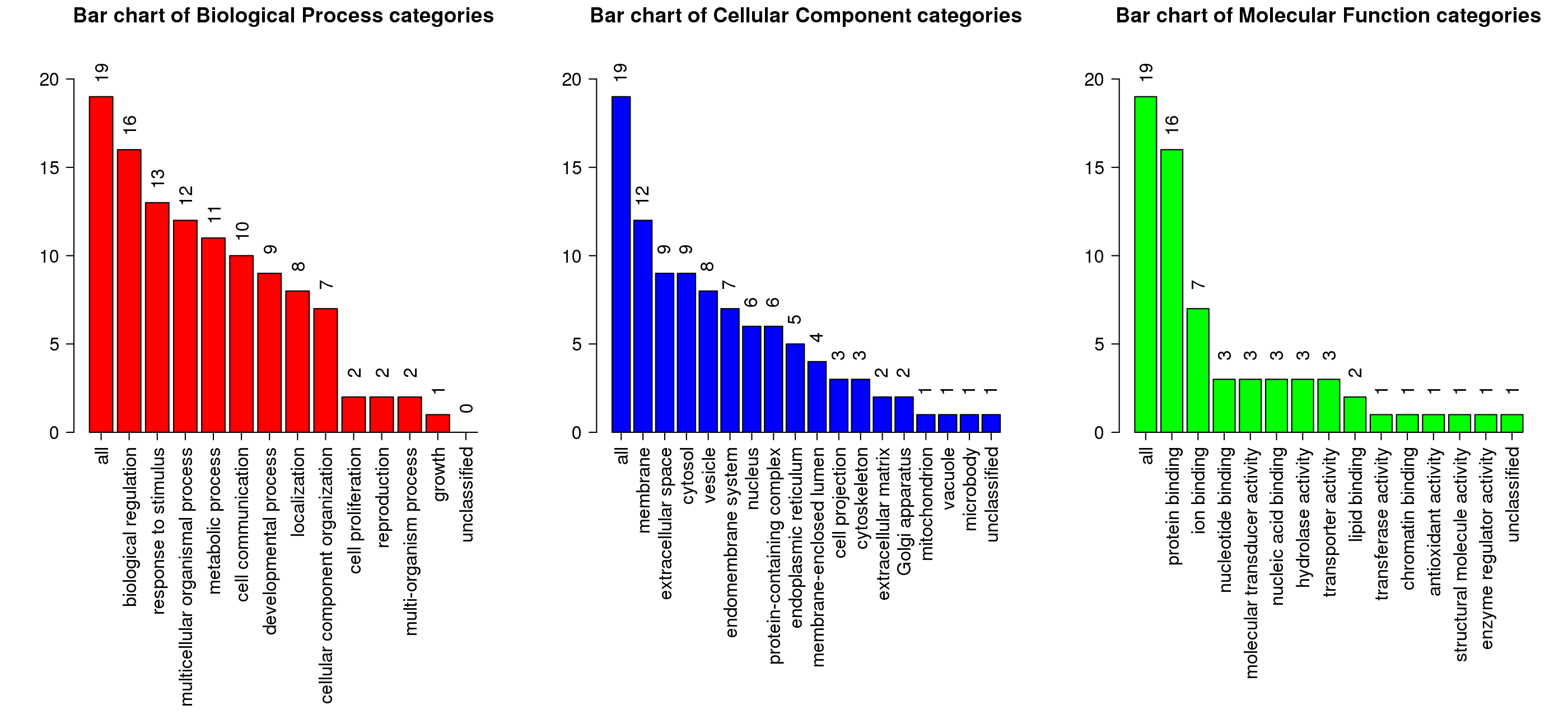

Supplement: Supplementary file 1 [file jcm-10-00407-s001.zip › sup/Supplementary_File_4/ORA_Webgestalt/ORA_down_Disease_GLAD4U/goslim_summary_wg_result1609843757.png]

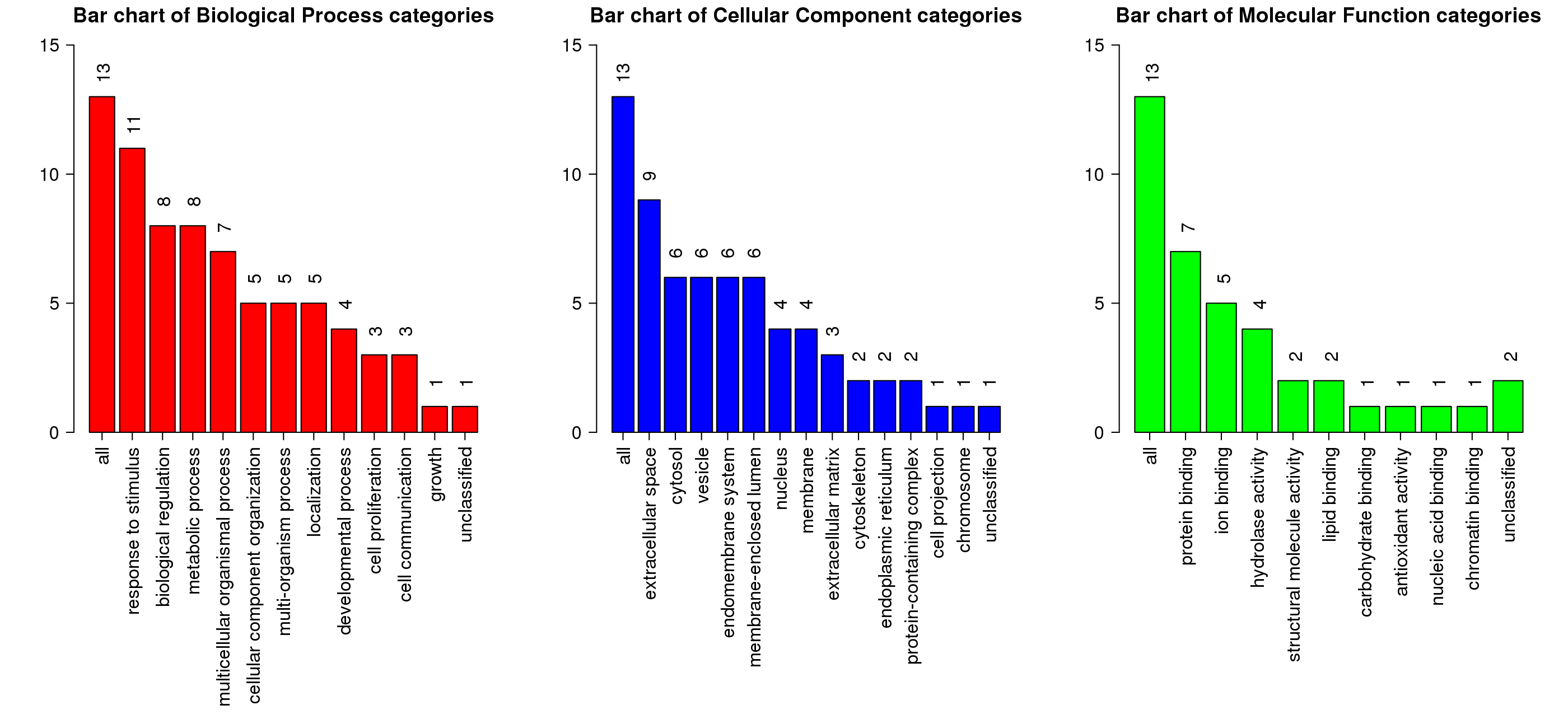

Supplement: Supplementary file 1 [file jcm-10-00407-s001.zip › sup/Supplementary_File_4/ORA_Webgestalt/ORA_up_GO_CC/goslim_summary_wg_result1609844545.png]

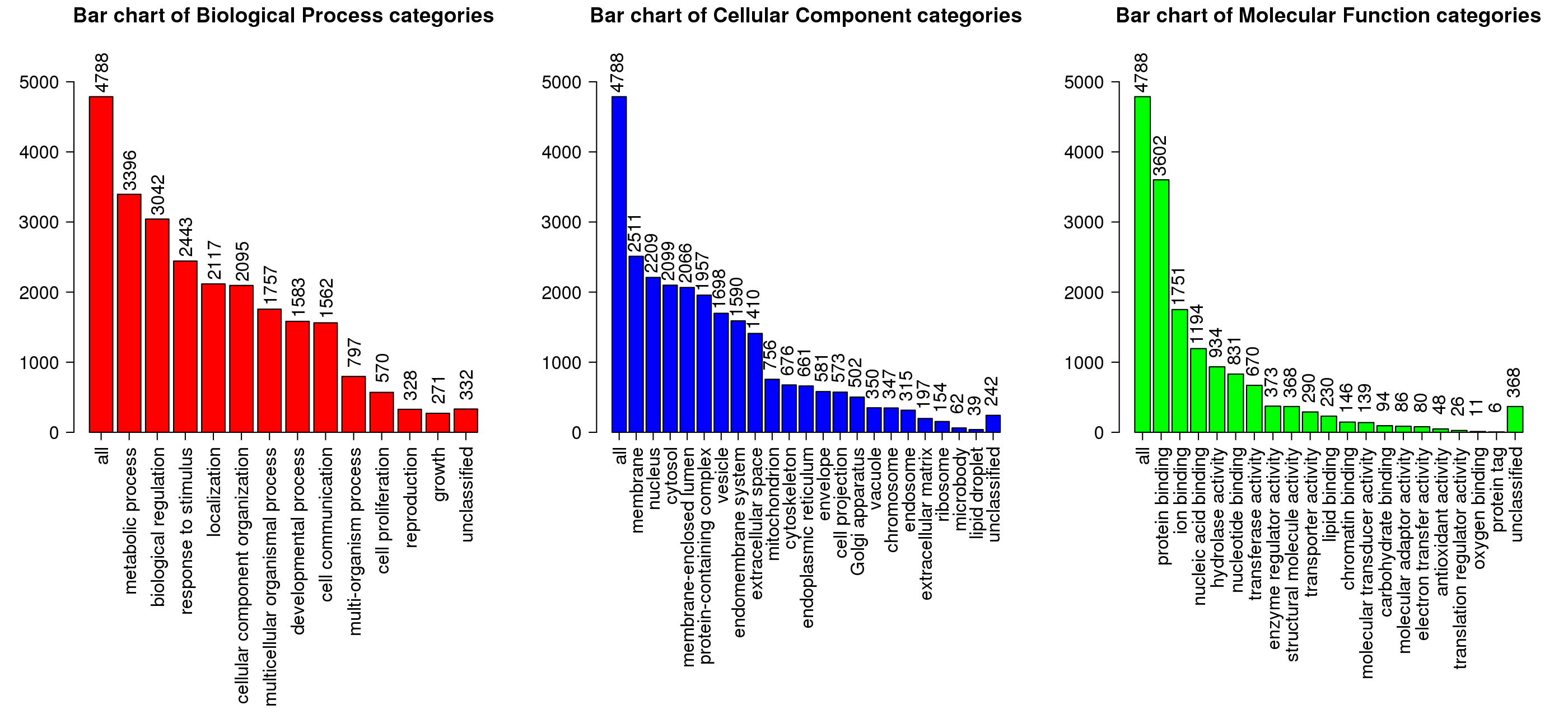

Supplement: Supplementary file 1 [file jcm-10-00407-s001.zip › sup/Supplementary_File_6/GSEA_Webgestalt/GSEA_Disease_Disgenet/goslim_summary_wg_result1604400295.png]

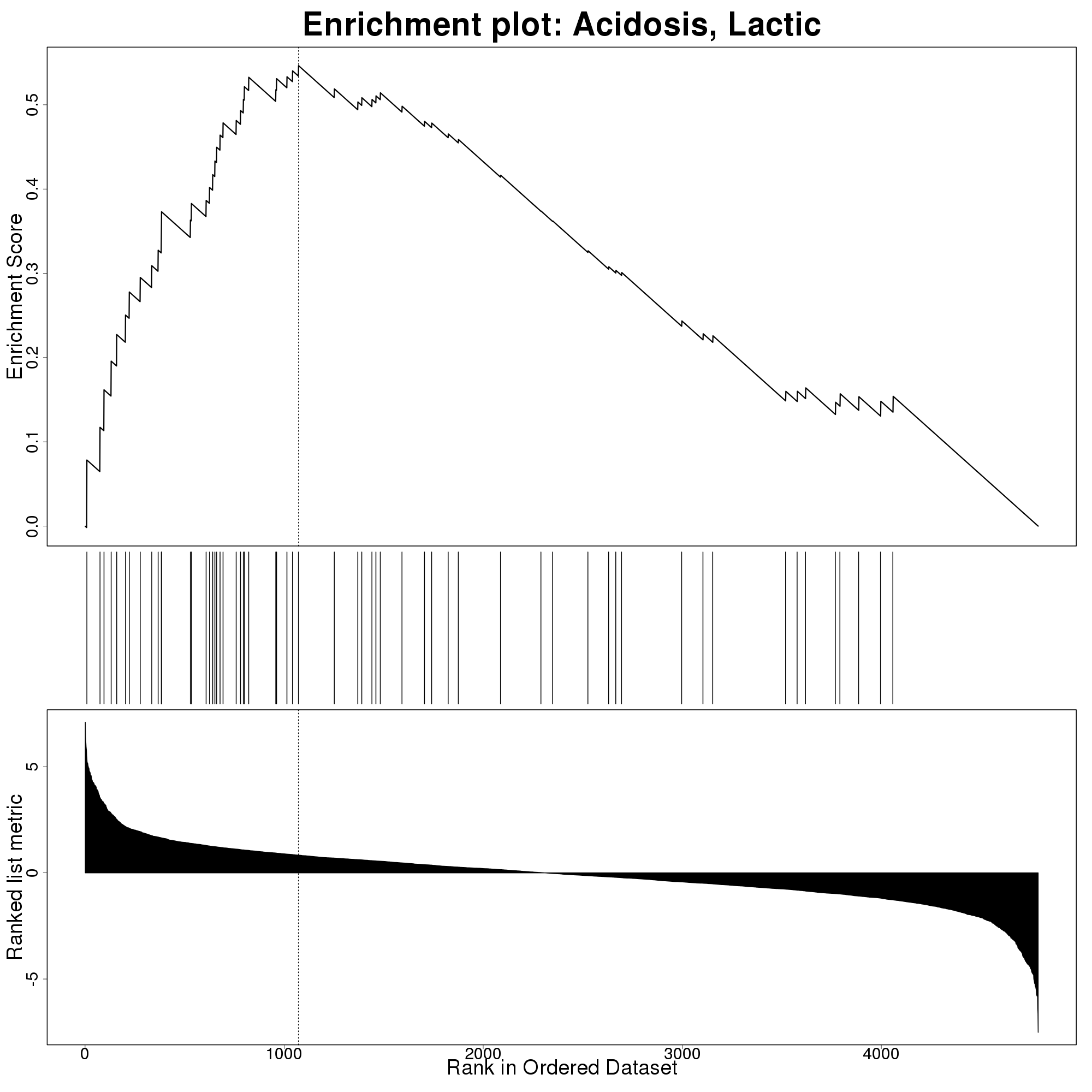

Supplement: Supplementary file 1 [file jcm-10-00407-s001.zip › sup/Supplementary_File_6/GSEA_Webgestalt/GSEA_Disease_Disgenet/Project_wg_result1604400295_GSEA/C0001125.png]

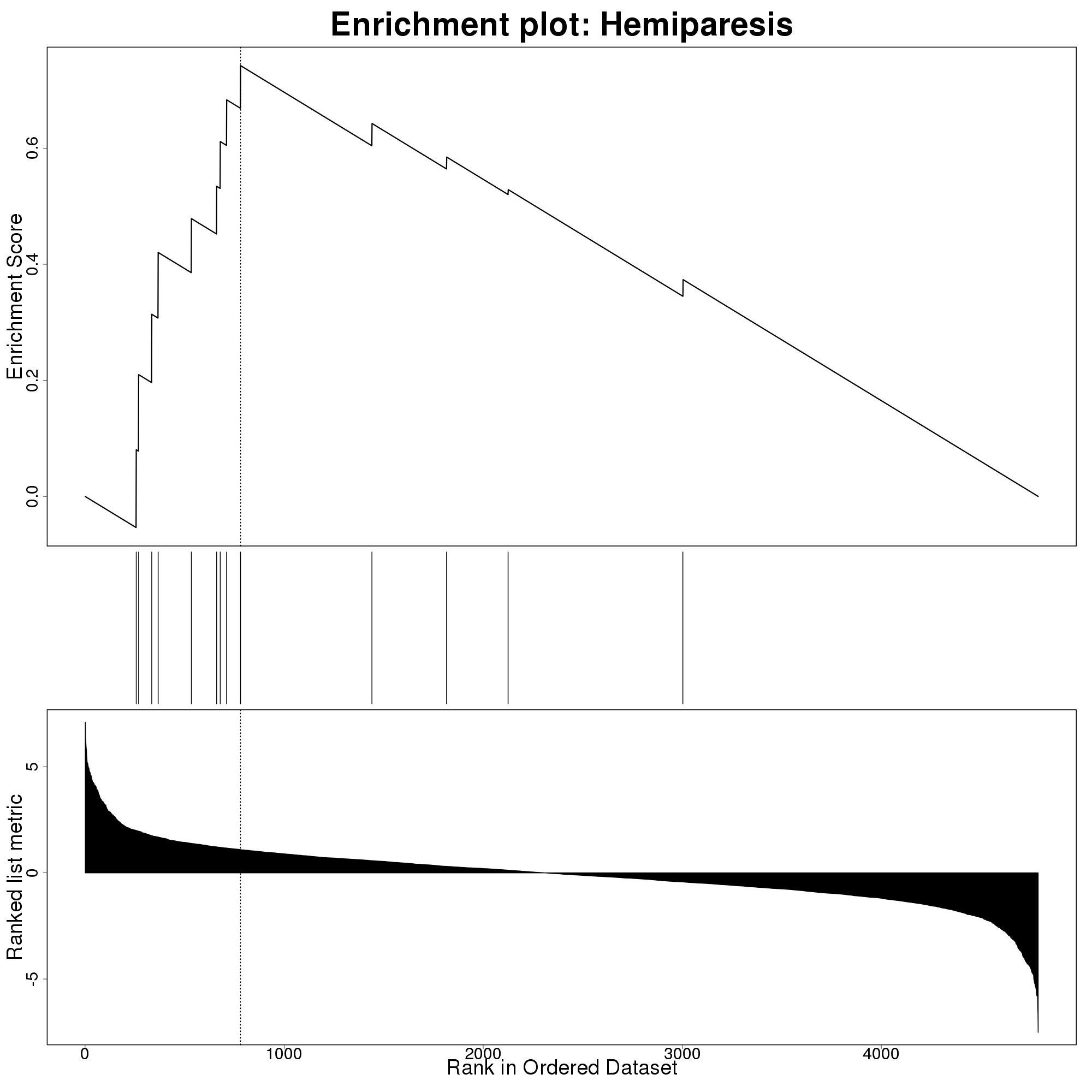

Supplement: Supplementary file 1 [file jcm-10-00407-s001.zip › sup/Supplementary_File_6/GSEA_Webgestalt/GSEA_Disease_Disgenet/Project_wg_result1604400295_GSEA/C0018989.png]

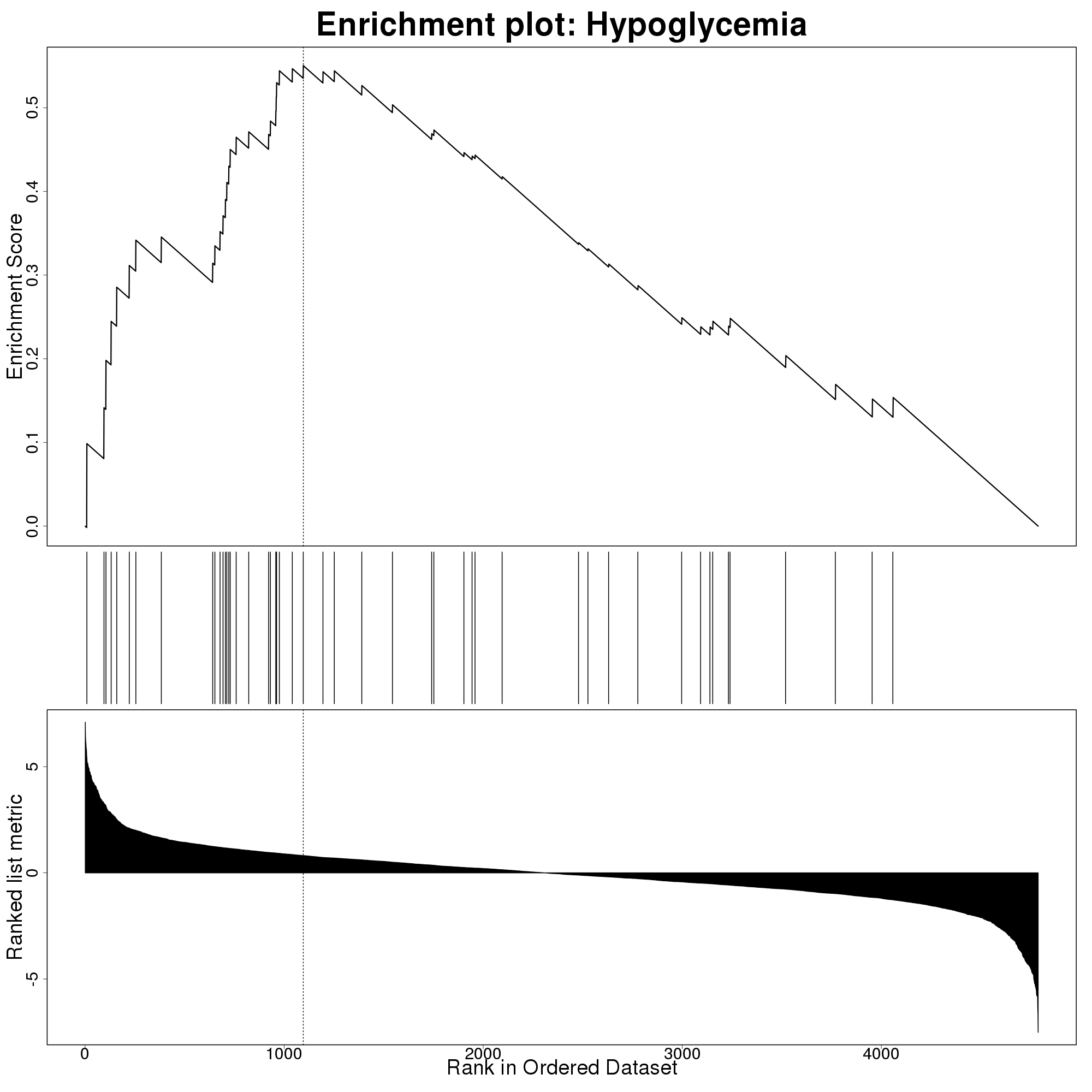

Supplement: Supplementary file 1 [file jcm-10-00407-s001.zip › sup/Supplementary_File_6/GSEA_Webgestalt/GSEA_Disease_Disgenet/Project_wg_result1604400295_GSEA/C0020615.png]

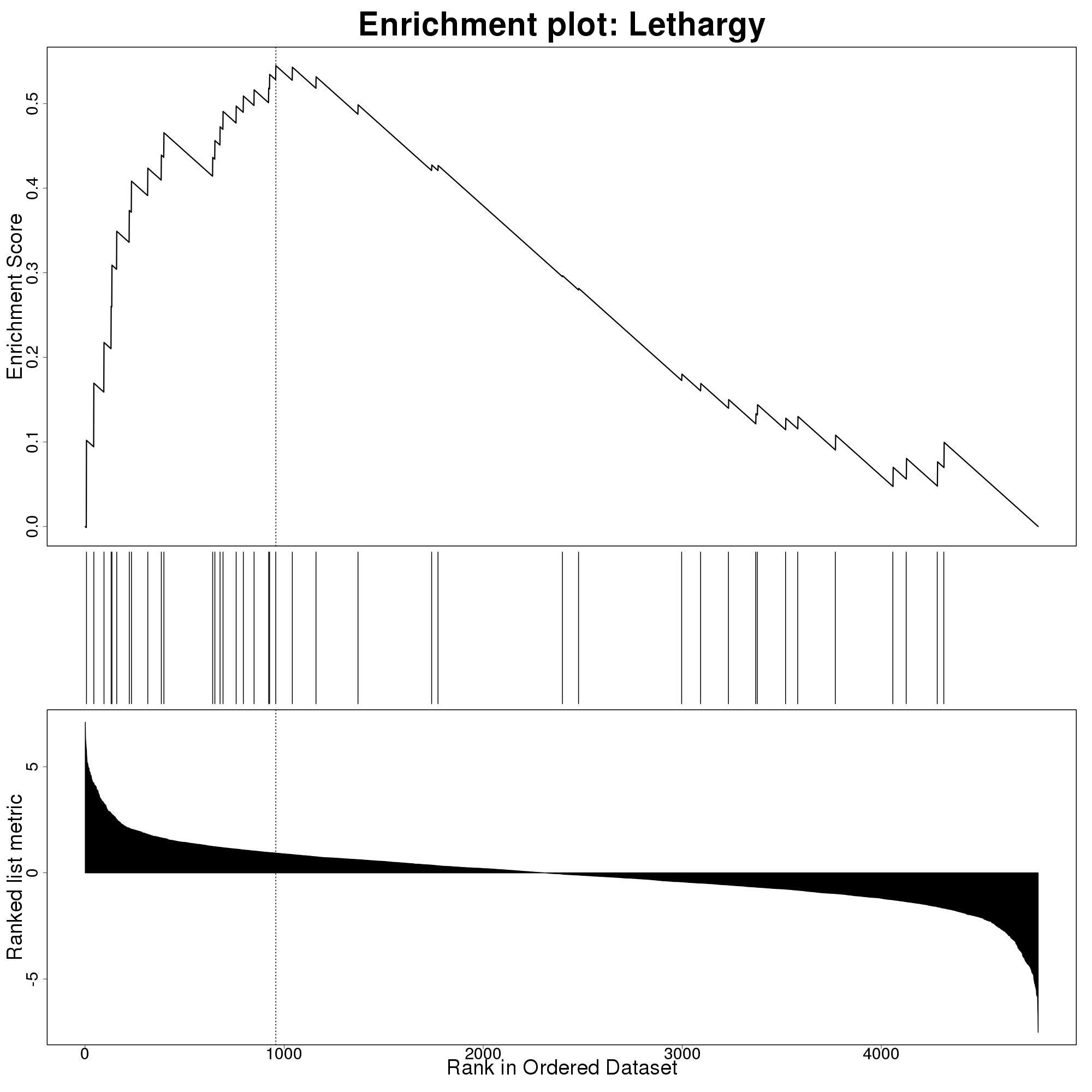

Supplement: Supplementary file 1 [file jcm-10-00407-s001.zip › sup/Supplementary_File_6/GSEA_Webgestalt/GSEA_Disease_Disgenet/Project_wg_result1604400295_GSEA/C0023380.png]

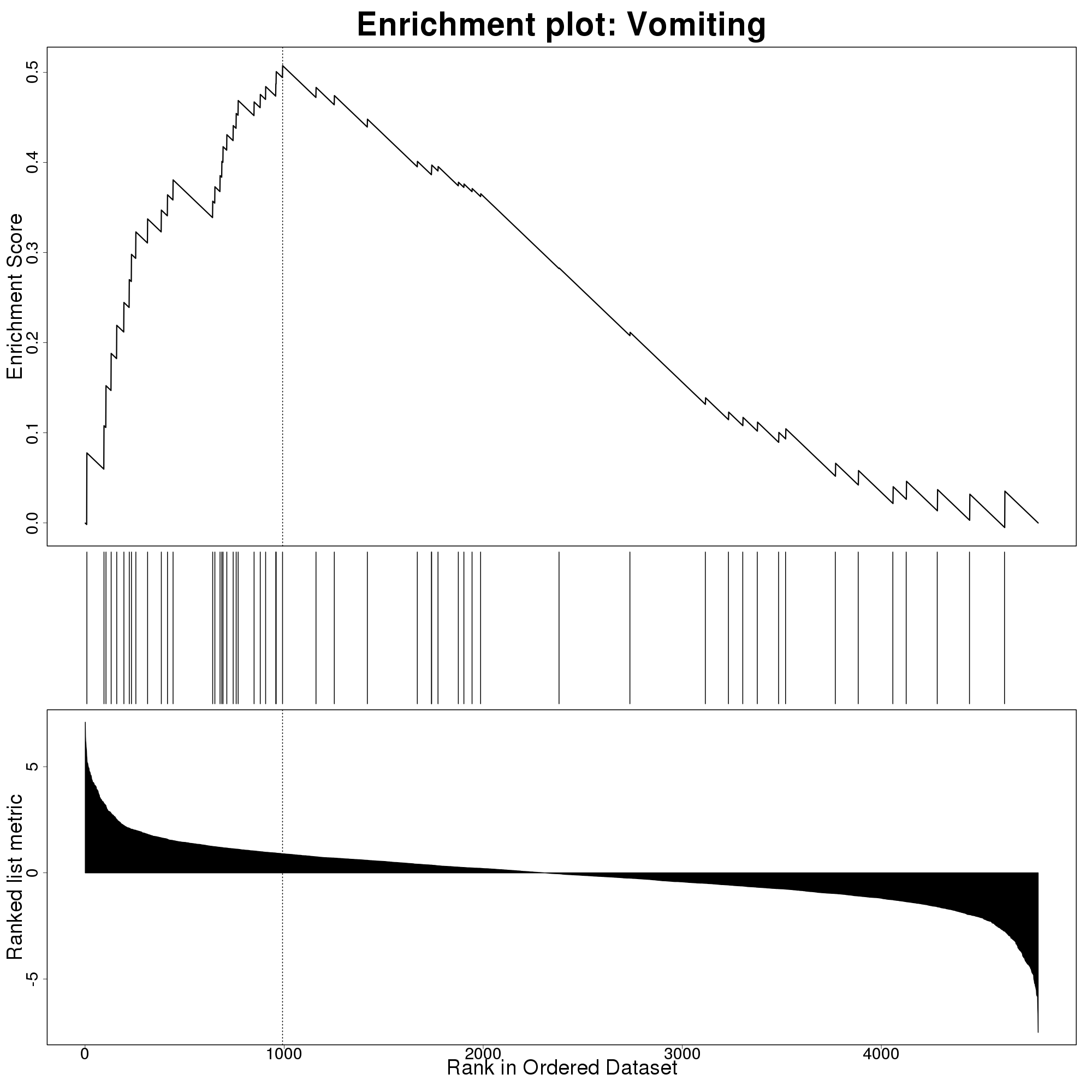

Supplement: Supplementary file 1 [file jcm-10-00407-s001.zip › sup/Supplementary_File_6/GSEA_Webgestalt/GSEA_Disease_Disgenet/Project_wg_result1604400295_GSEA/C0042963.png]

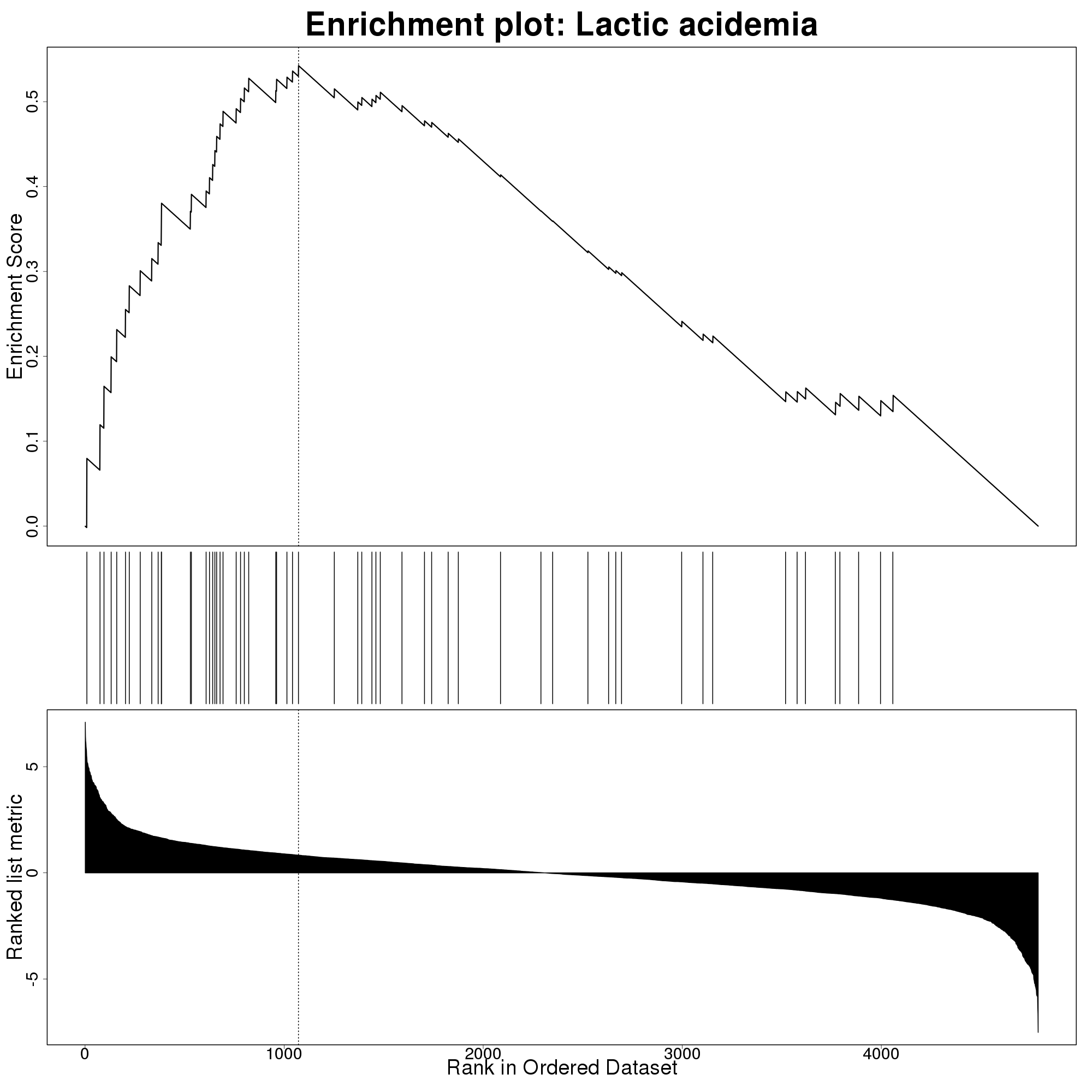

Supplement: Supplementary file 1 [file jcm-10-00407-s001.zip › sup/Supplementary_File_6/GSEA_Webgestalt/GSEA_Disease_Disgenet/Project_wg_result1604400295_GSEA/C0347959.png]

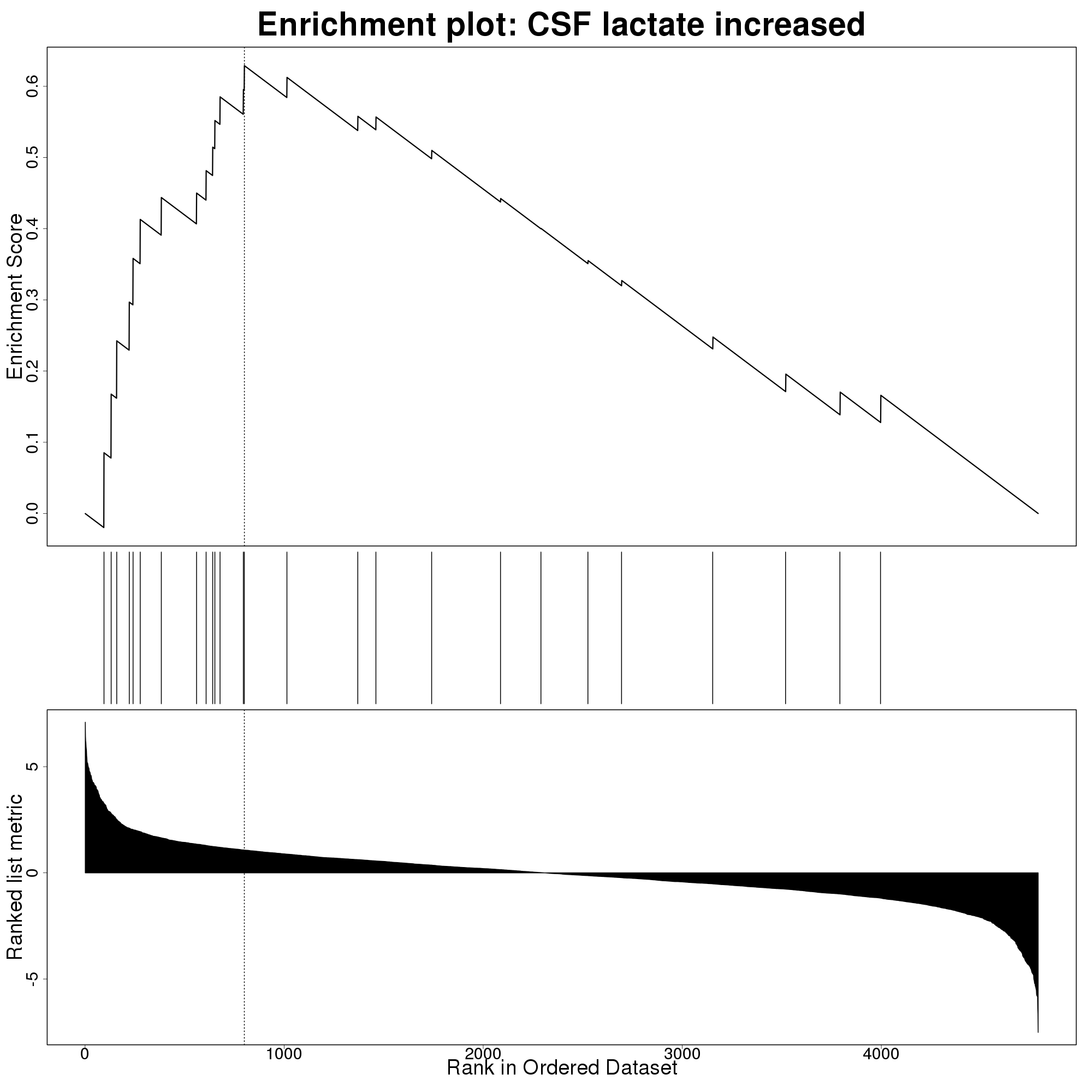

Supplement: Supplementary file 1 [file jcm-10-00407-s001.zip › sup/Supplementary_File_6/GSEA_Webgestalt/GSEA_Disease_Disgenet/Project_wg_result1604400295_GSEA/C1167918.png]

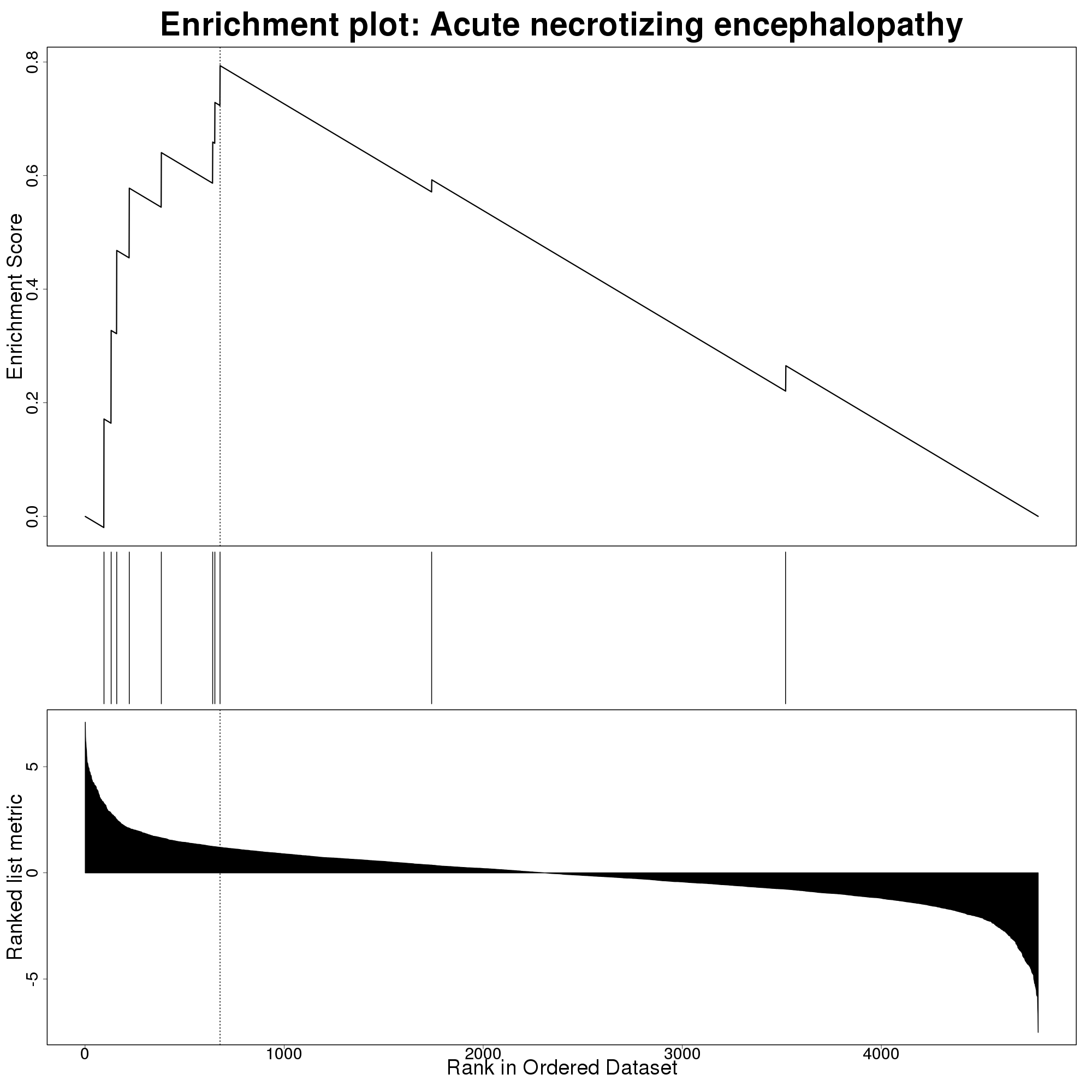

Supplement: Supplementary file 1 [file jcm-10-00407-s001.zip › sup/Supplementary_File_6/GSEA_Webgestalt/GSEA_Disease_Disgenet/Project_wg_result1604400295_GSEA/C1855020.png]

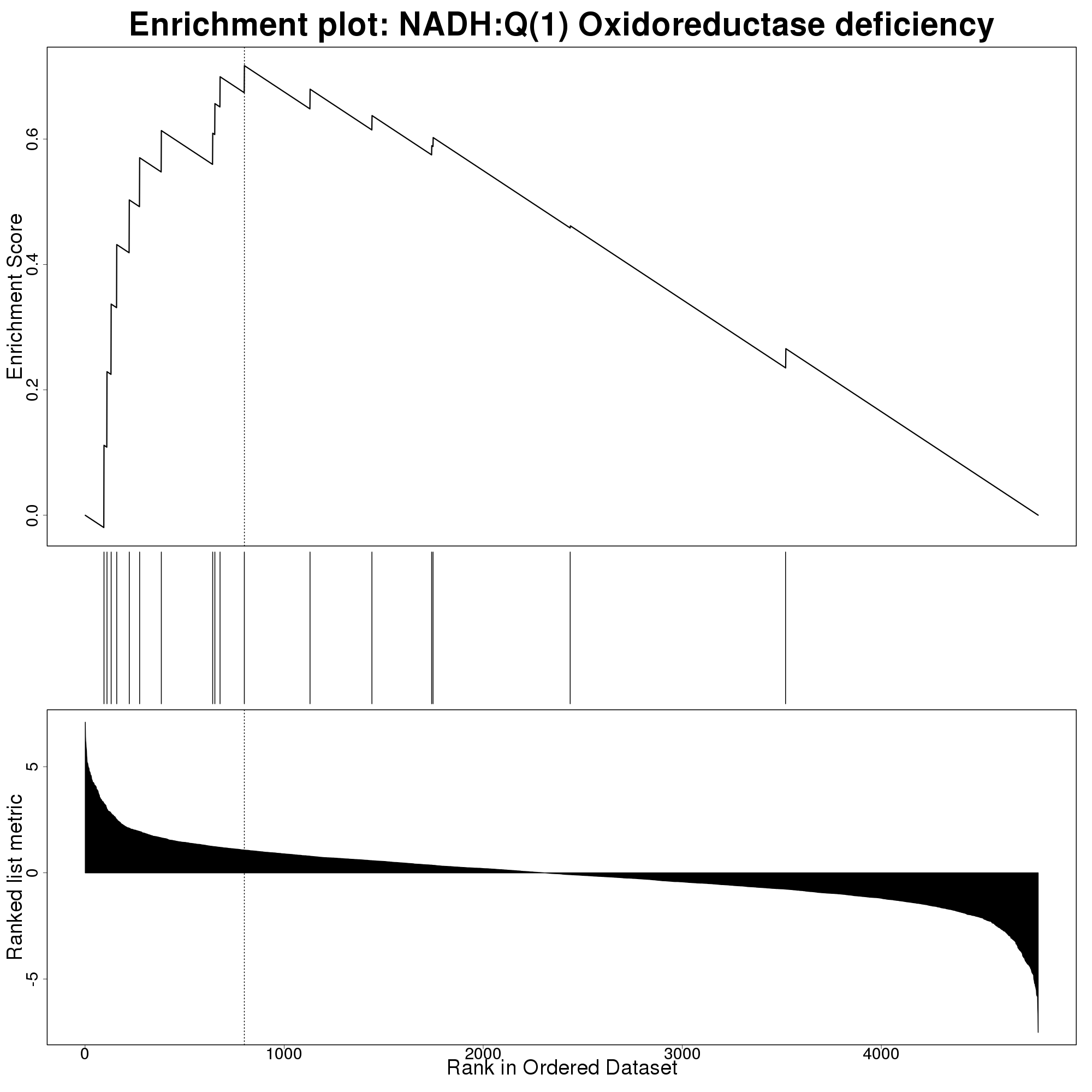

Supplement: Supplementary file 1 [file jcm-10-00407-s001.zip › sup/Supplementary_File_6/GSEA_Webgestalt/GSEA_Disease_Disgenet/Project_wg_result1604400295_GSEA/C2936907.png]

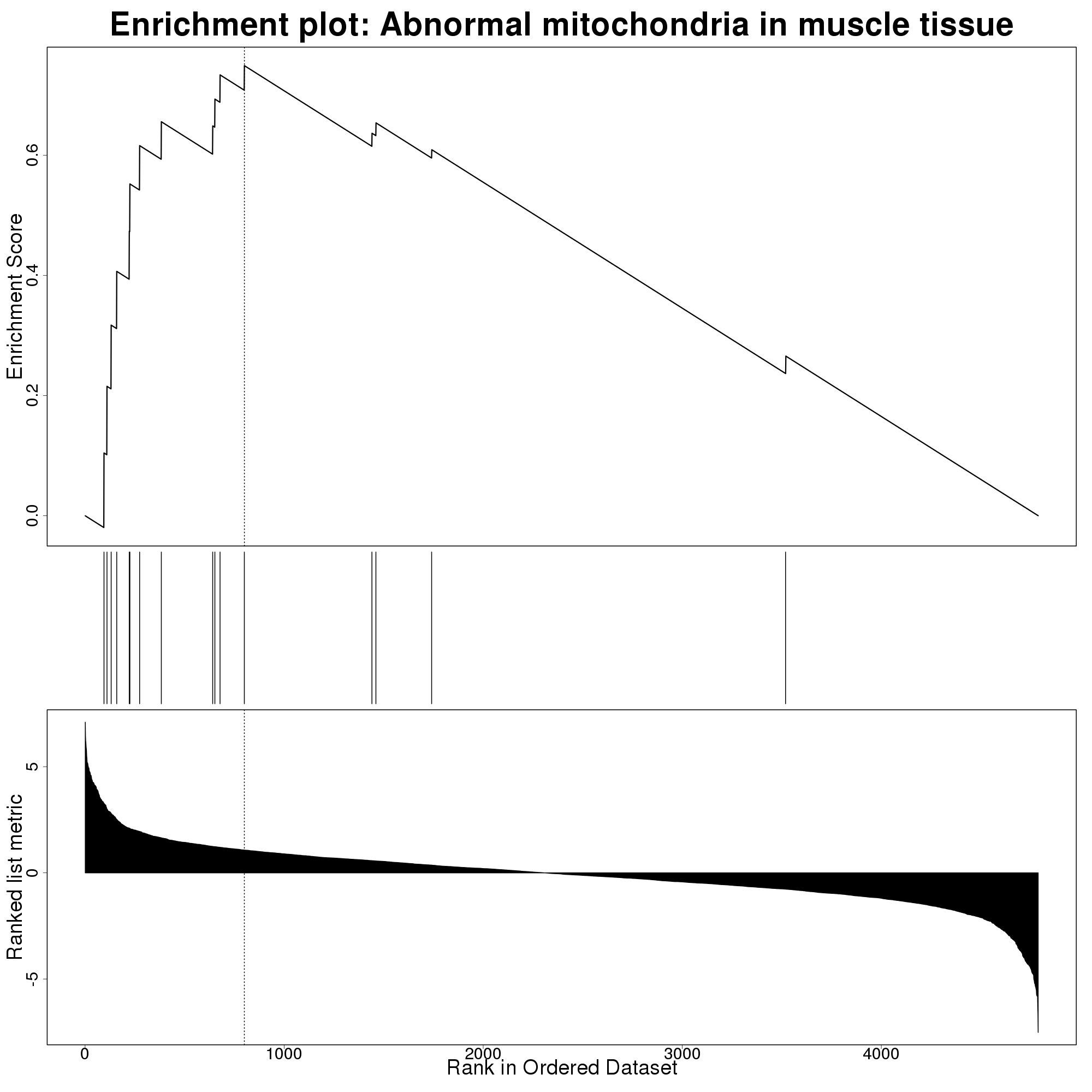

Supplement: Supplementary file 1 [file jcm-10-00407-s001.zip › sup/Supplementary_File_6/GSEA_Webgestalt/GSEA_Disease_Disgenet/Project_wg_result1604400295_GSEA/C4021546.png]

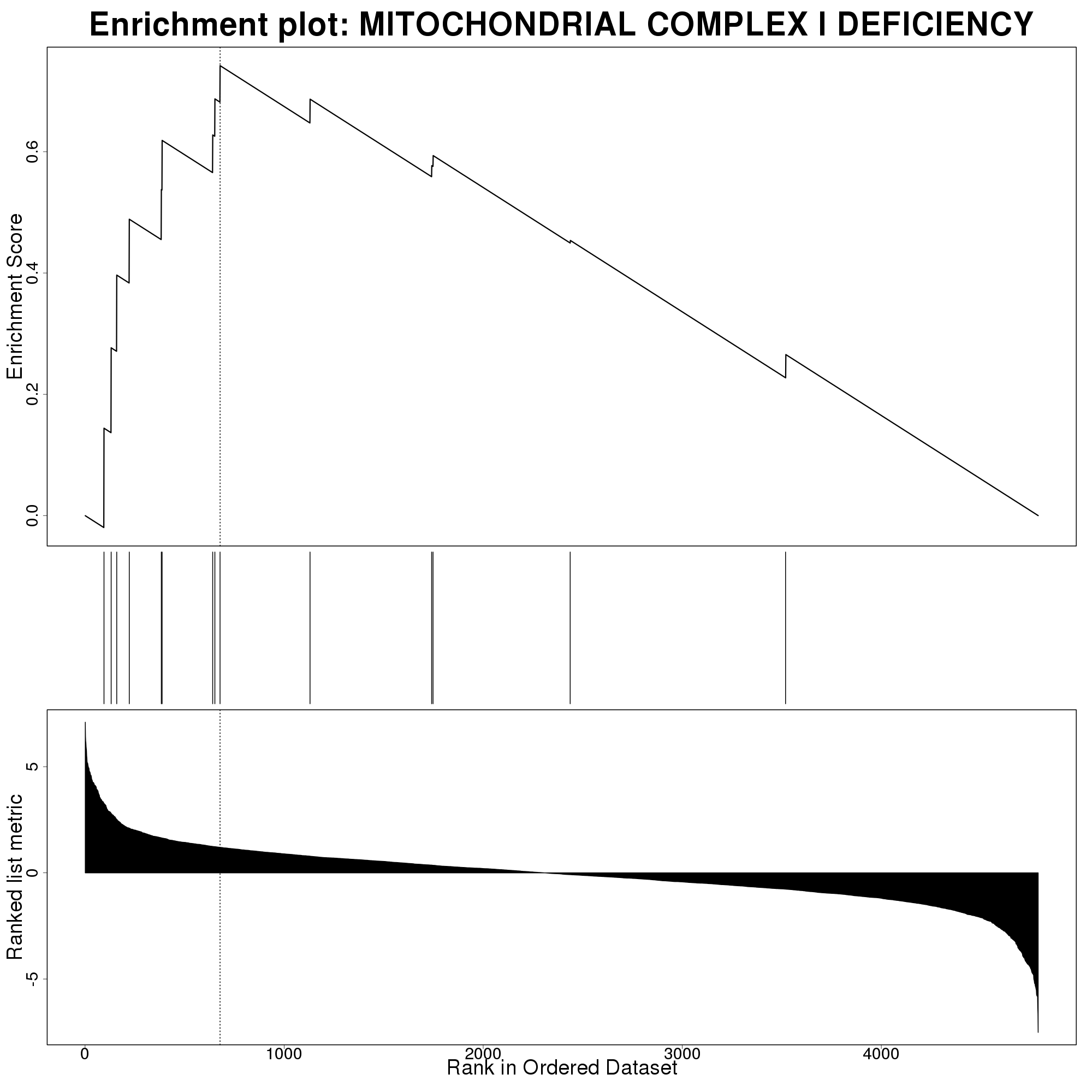

Supplement: Supplementary file 1 [file jcm-10-00407-s001.zip › sup/Supplementary_File_6/GSEA_Webgestalt/GSEA_Disease_OMIM/Project_wg_result1604400314_GSEA/252010.png]

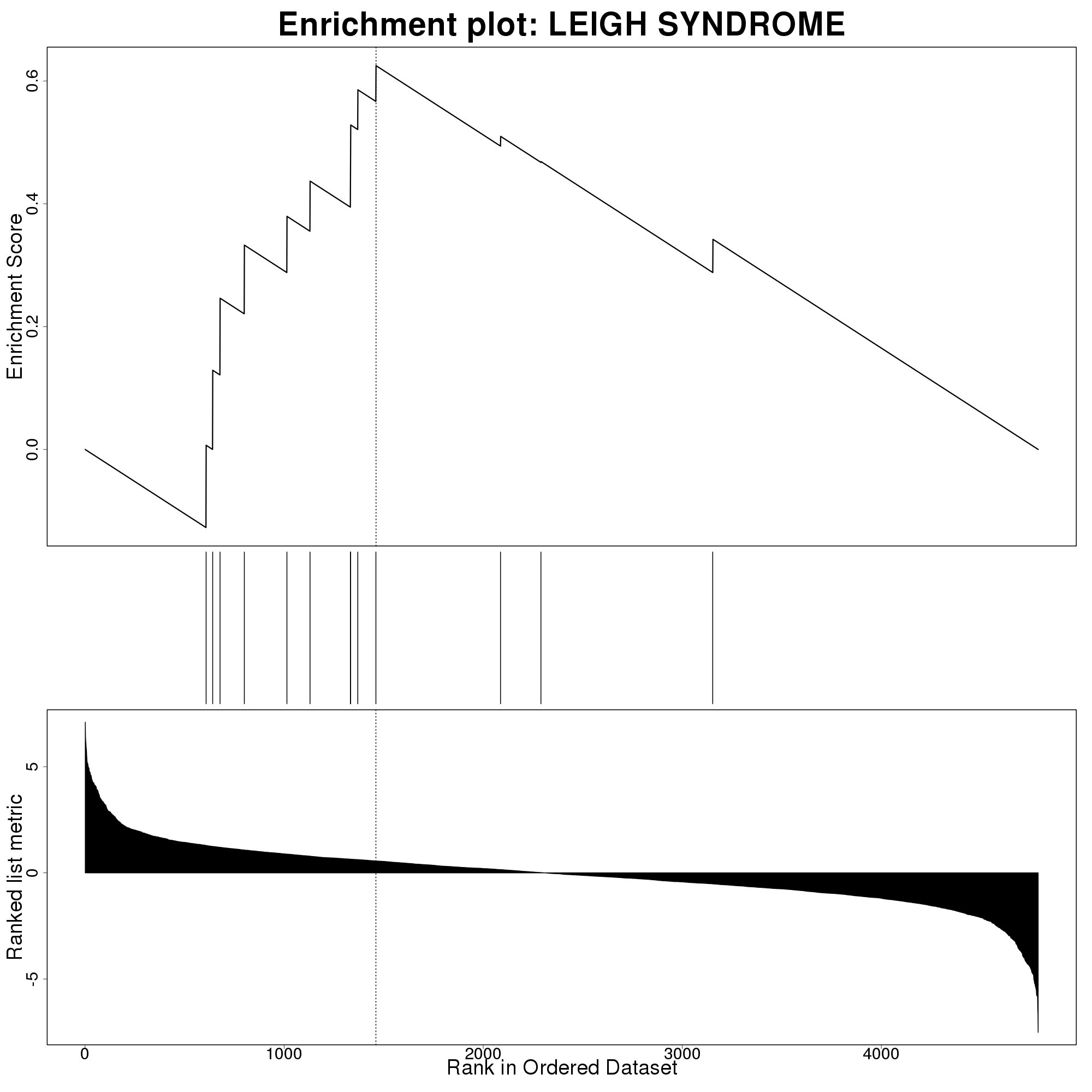

Supplement: Supplementary file 1 [file jcm-10-00407-s001.zip › sup/Supplementary_File_6/GSEA_Webgestalt/GSEA_Disease_OMIM/Project_wg_result1604400314_GSEA/256000.png]

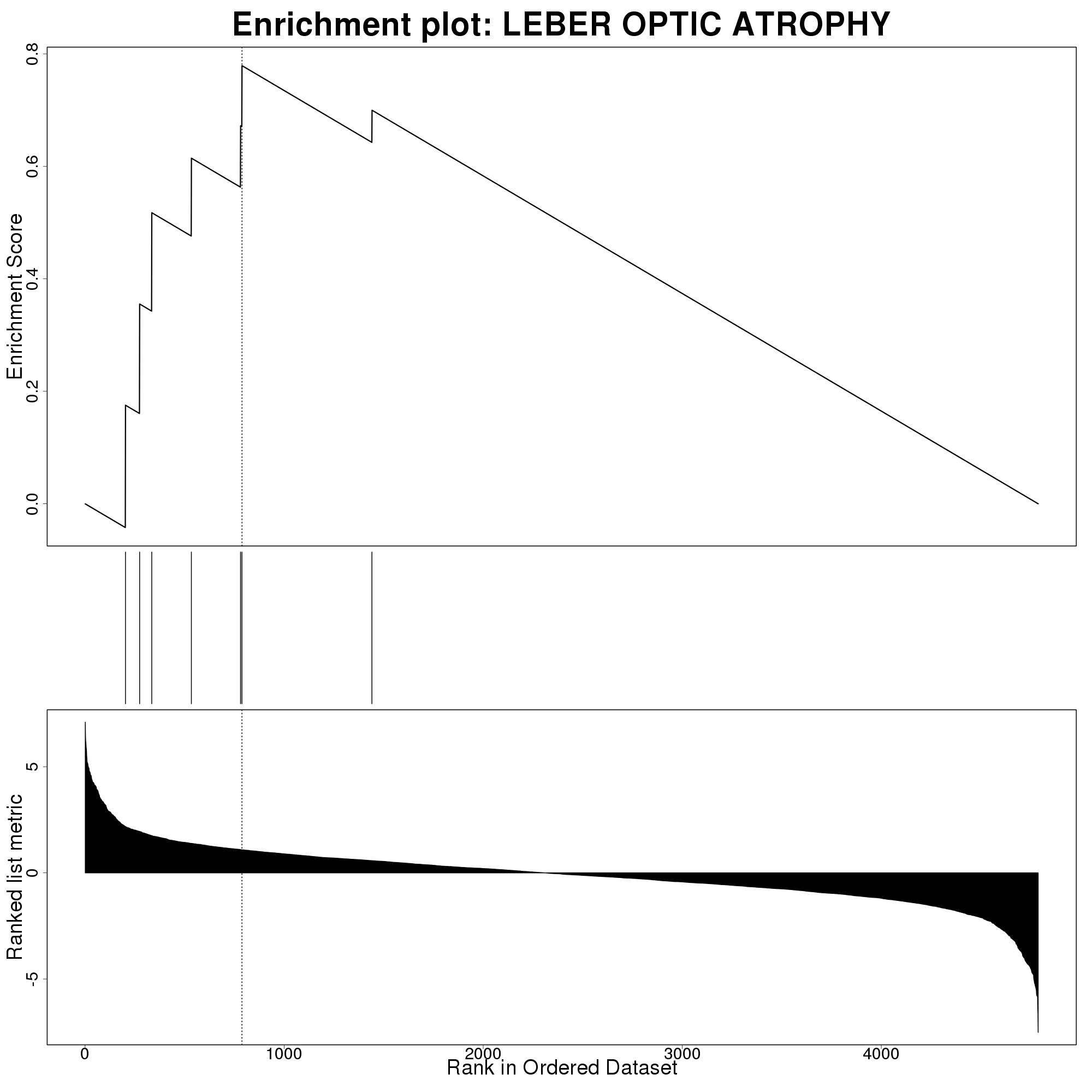

Supplement: Supplementary file 1 [file jcm-10-00407-s001.zip › sup/Supplementary_File_6/GSEA_Webgestalt/GSEA_Disease_OMIM/Project_wg_result1604400314_GSEA/535000.png]

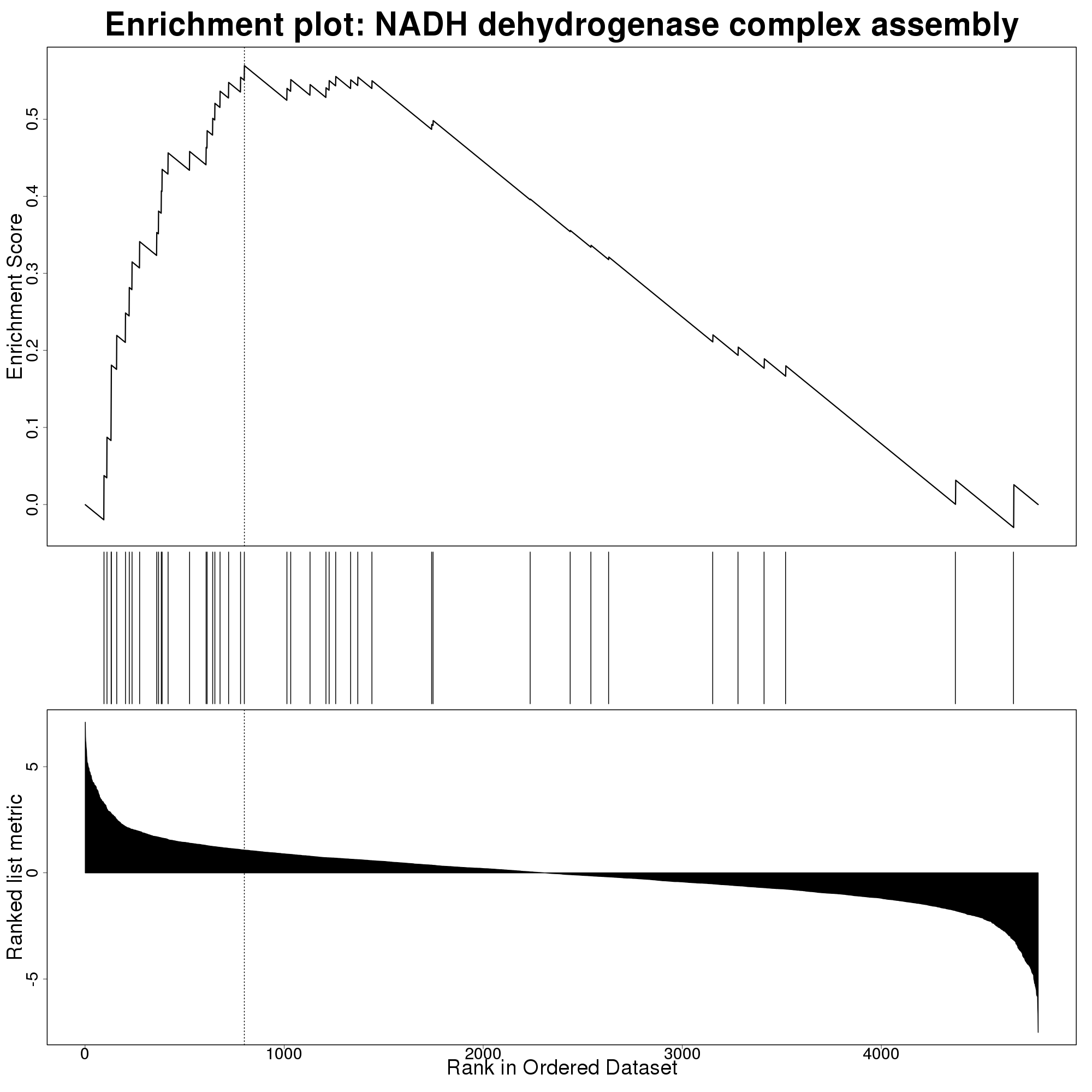

Supplement: Supplementary file 1 [file jcm-10-00407-s001.zip › sup/Supplementary_File_6/GSEA_Webgestalt/GSEA_GO_BP/Project_wg_result1604400201_GSEA/GO_0010257.png]

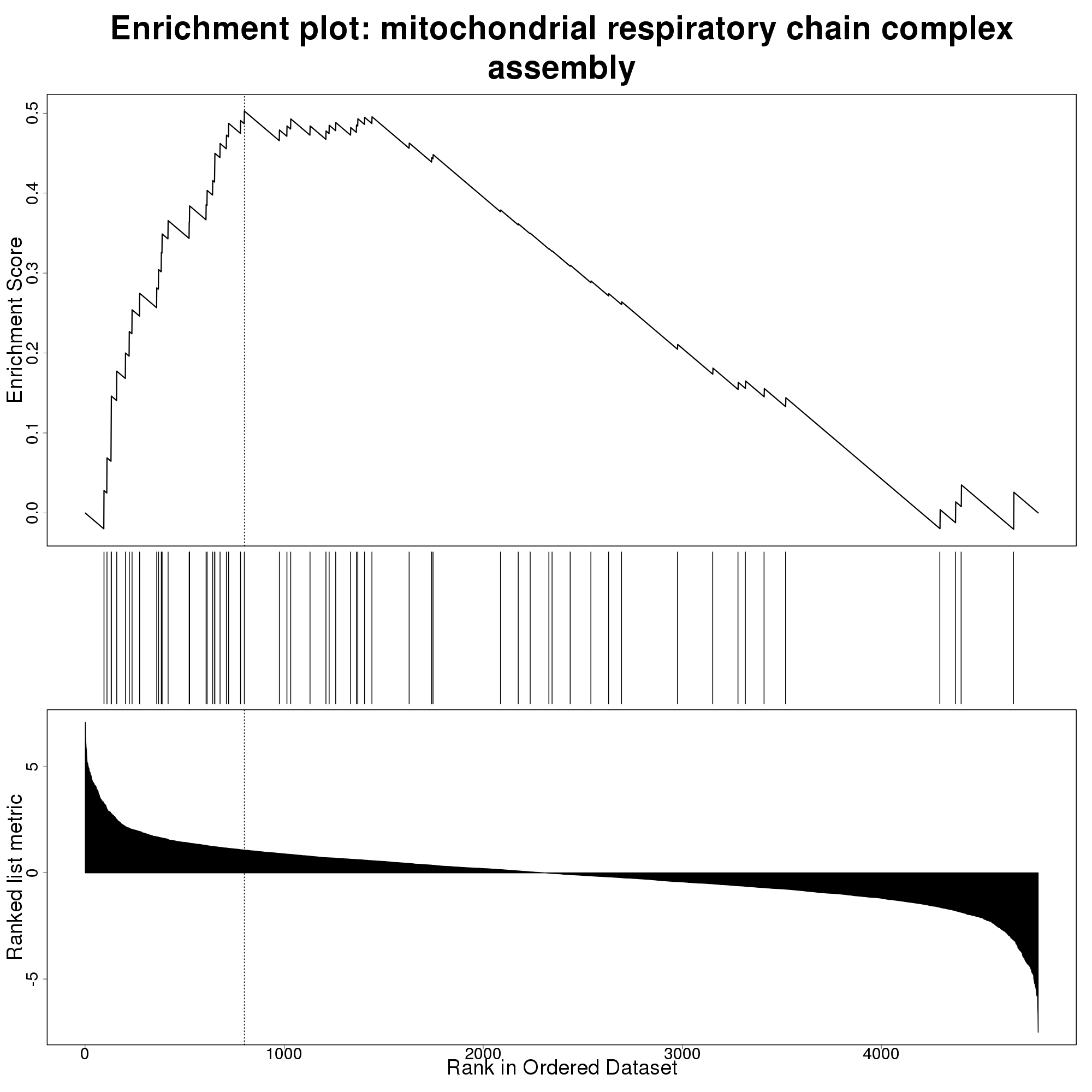

Supplement: Supplementary file 1 [file jcm-10-00407-s001.zip › sup/Supplementary_File_6/GSEA_Webgestalt/GSEA_GO_BP/Project_wg_result1604400201_GSEA/GO_0033108.png]

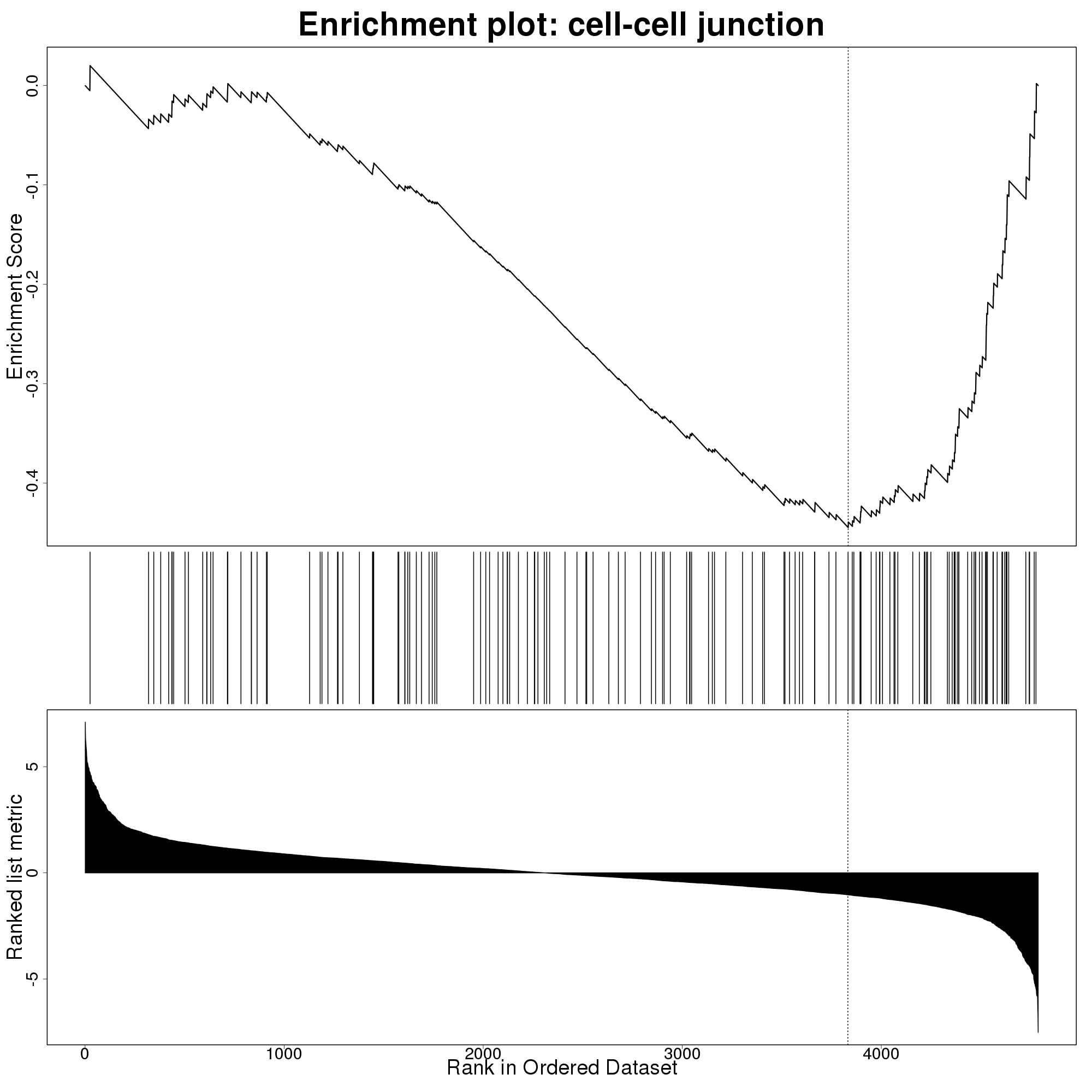

Supplement: Supplementary file 1 [file jcm-10-00407-s001.zip › sup/Supplementary_File_6/GSEA_Webgestalt/GSEA_GO_CC/Project_wg_result1604400208_GSEA/GO_0005911.png]

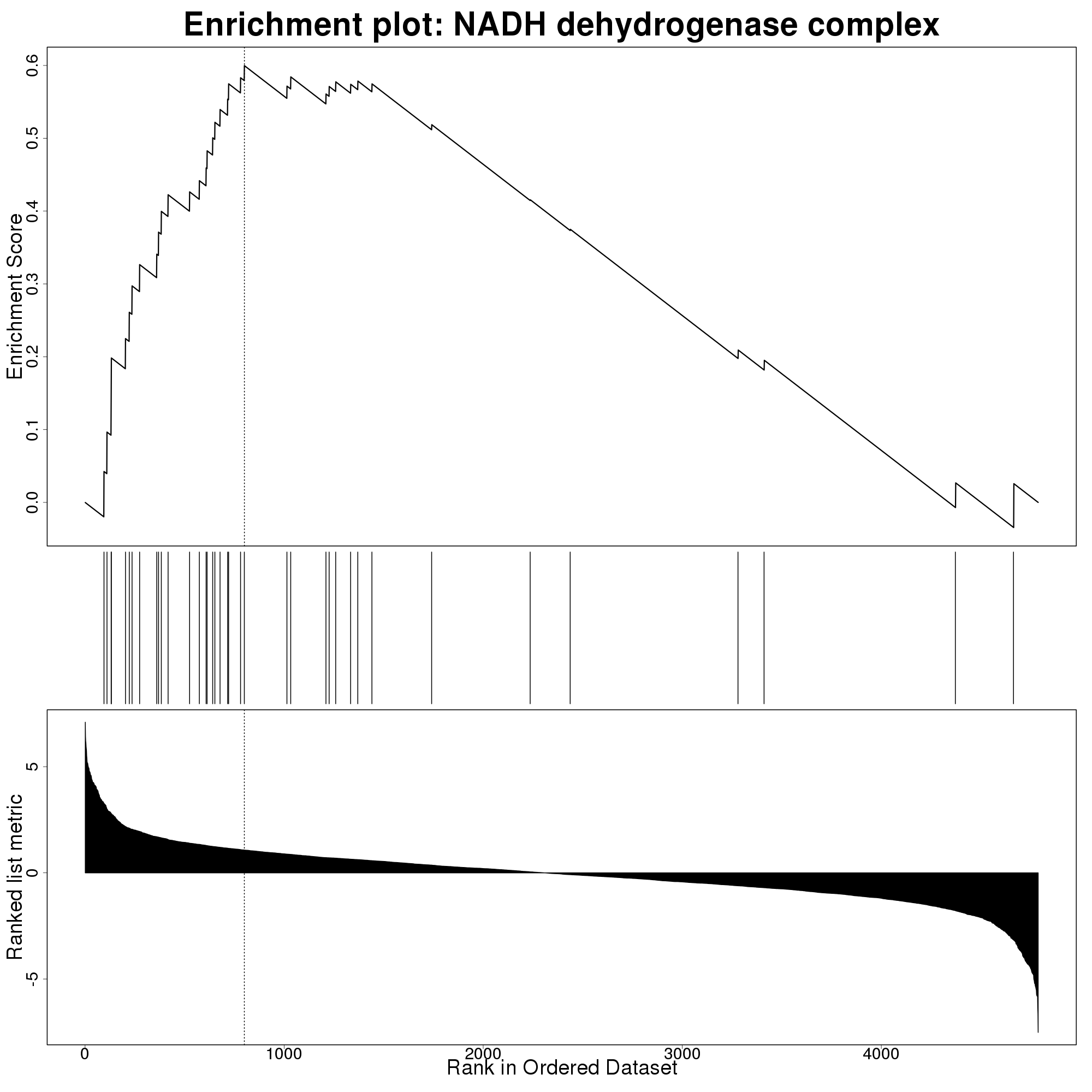

Supplement: Supplementary file 1 [file jcm-10-00407-s001.zip › sup/Supplementary_File_6/GSEA_Webgestalt/GSEA_GO_CC/Project_wg_result1604400208_GSEA/GO_0030964.png]

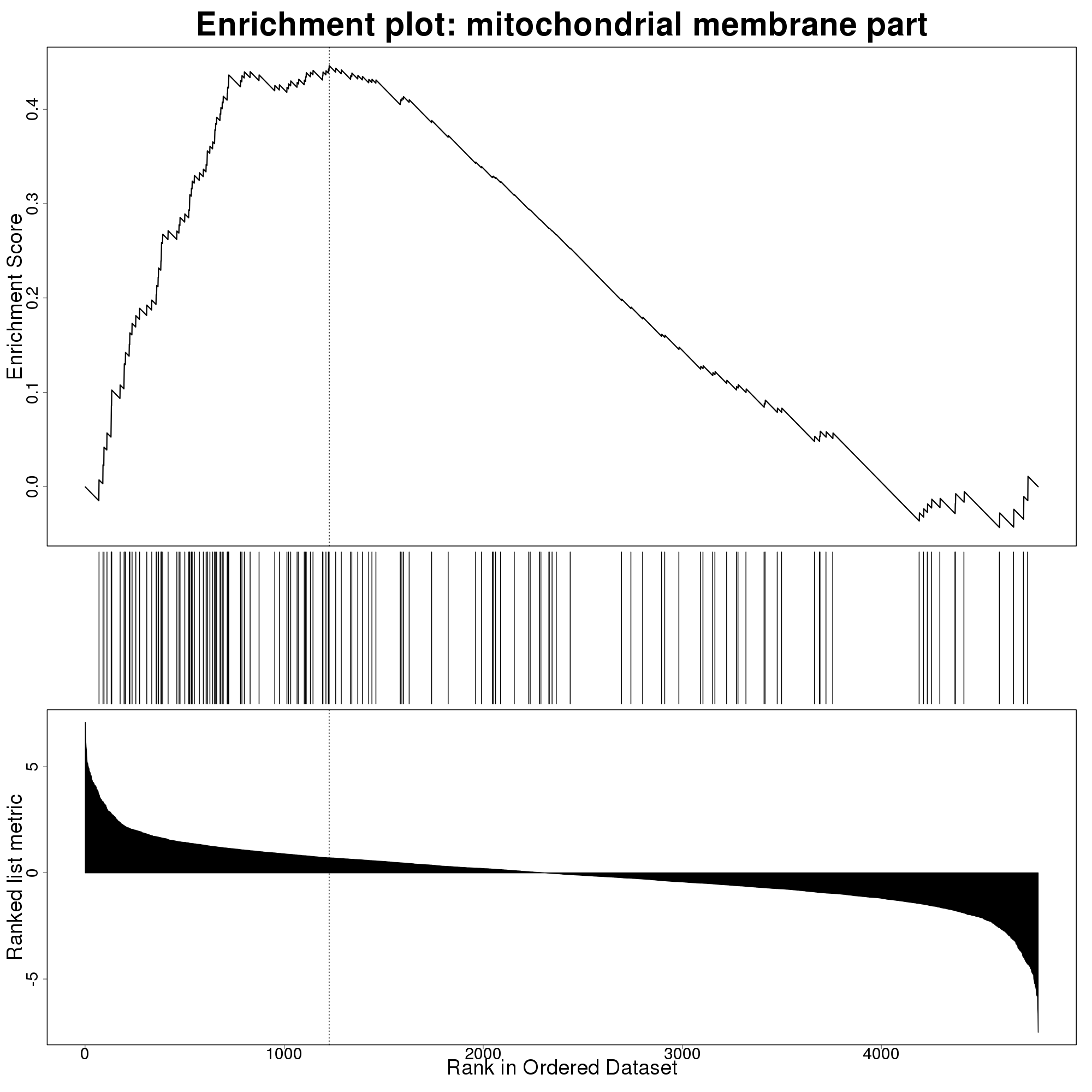

Supplement: Supplementary file 1 [file jcm-10-00407-s001.zip › sup/Supplementary_File_6/GSEA_Webgestalt/GSEA_GO_CC/Project_wg_result1604400208_GSEA/GO_0044455.png]

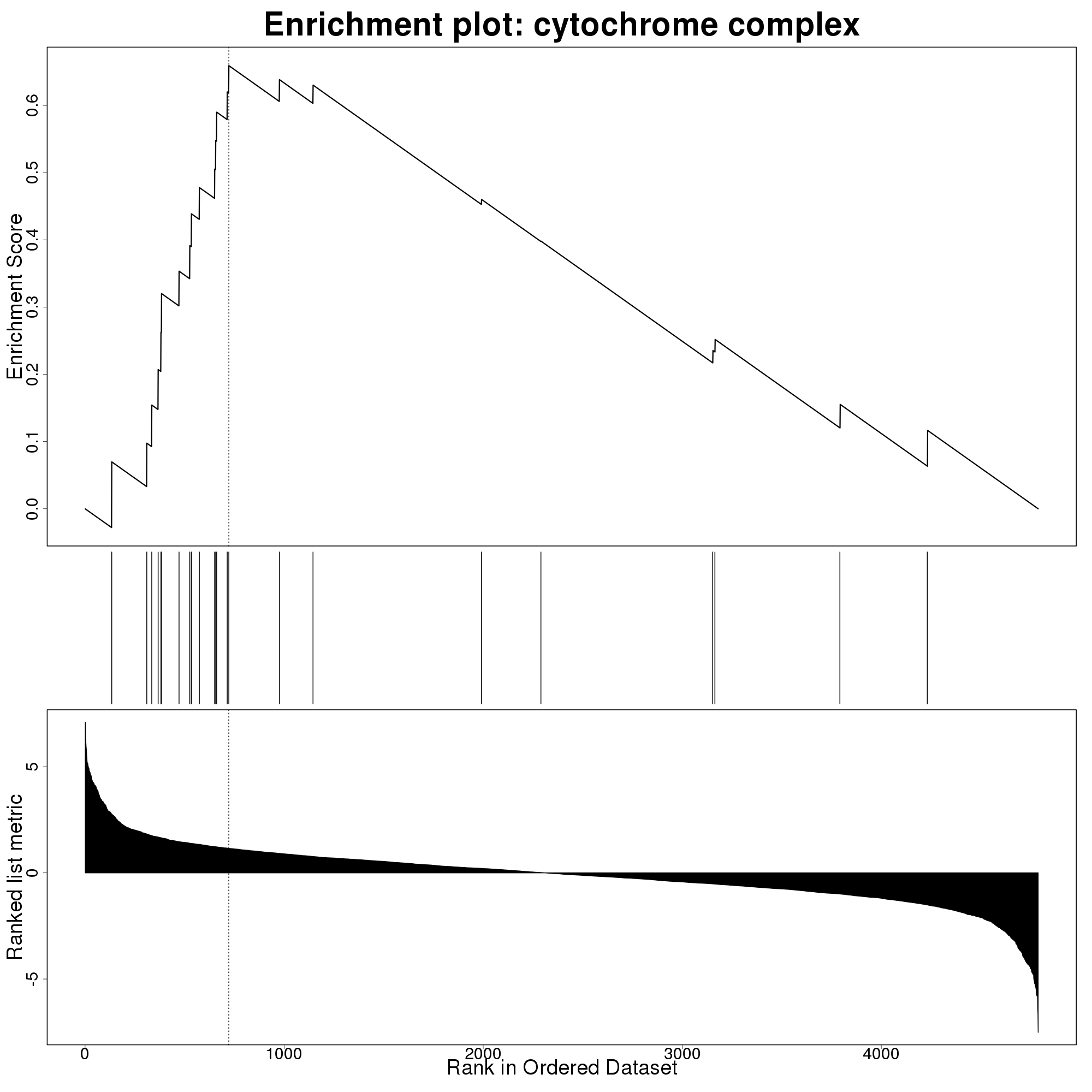

Supplement: Supplementary file 1 [file jcm-10-00407-s001.zip › sup/Supplementary_File_6/GSEA_Webgestalt/GSEA_GO_CC/Project_wg_result1604400208_GSEA/GO_0070069.png]

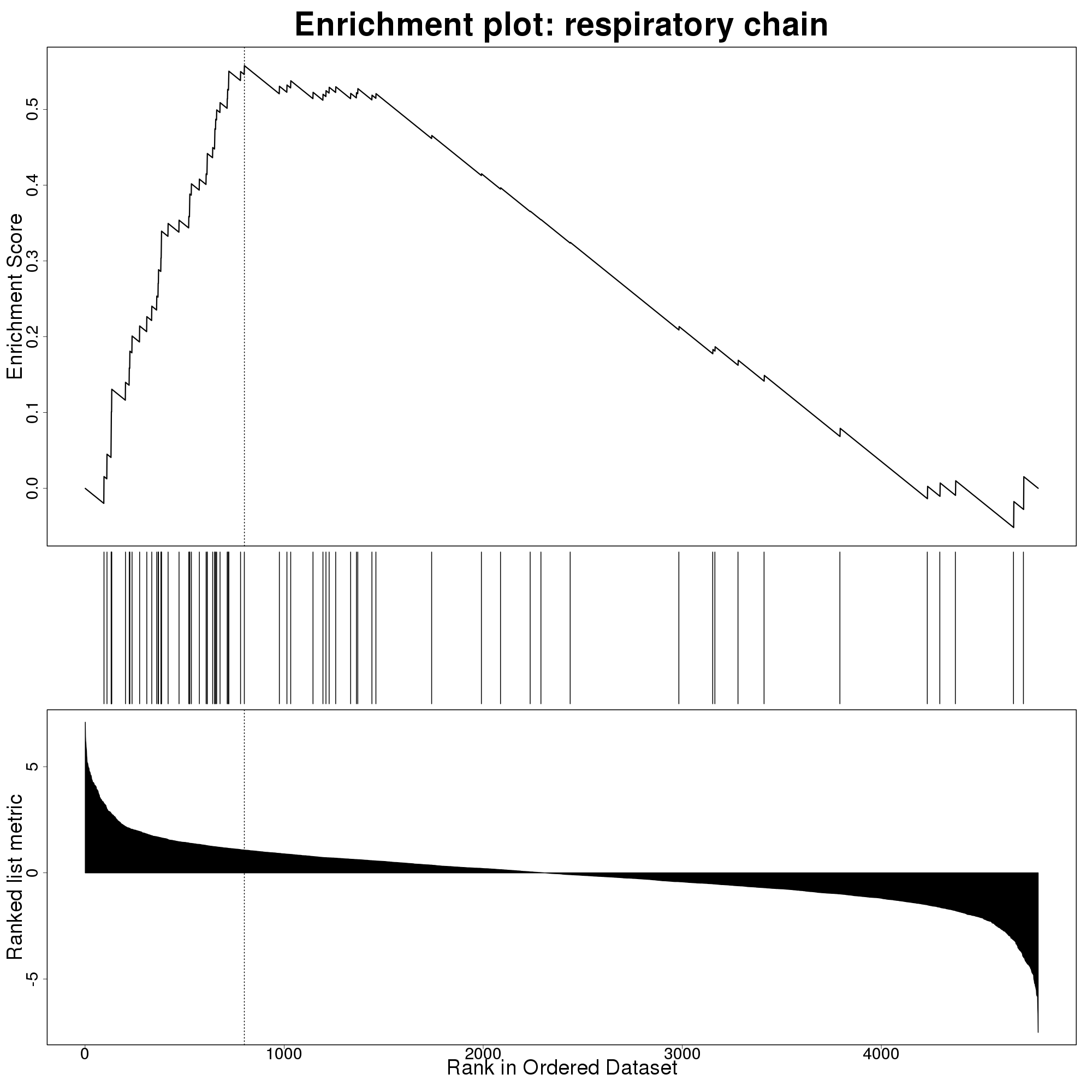

Supplement: Supplementary file 1 [file jcm-10-00407-s001.zip › sup/Supplementary_File_6/GSEA_Webgestalt/GSEA_GO_CC/Project_wg_result1604400208_GSEA/GO_0070469.png]

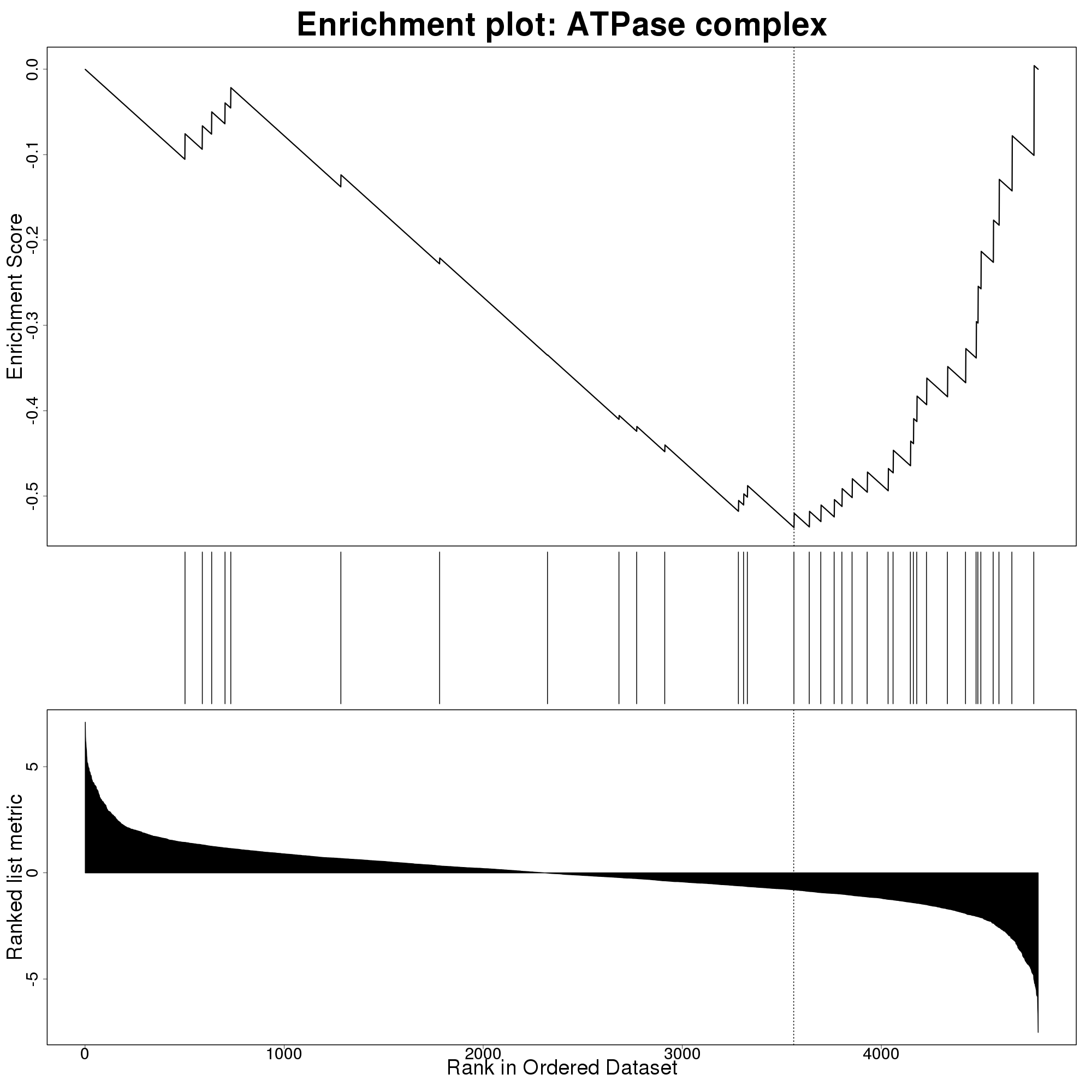

Supplement: Supplementary file 1 [file jcm-10-00407-s001.zip › sup/Supplementary_File_6/GSEA_Webgestalt/GSEA_GO_CC/Project_wg_result1604400208_GSEA/GO_1904949.png]

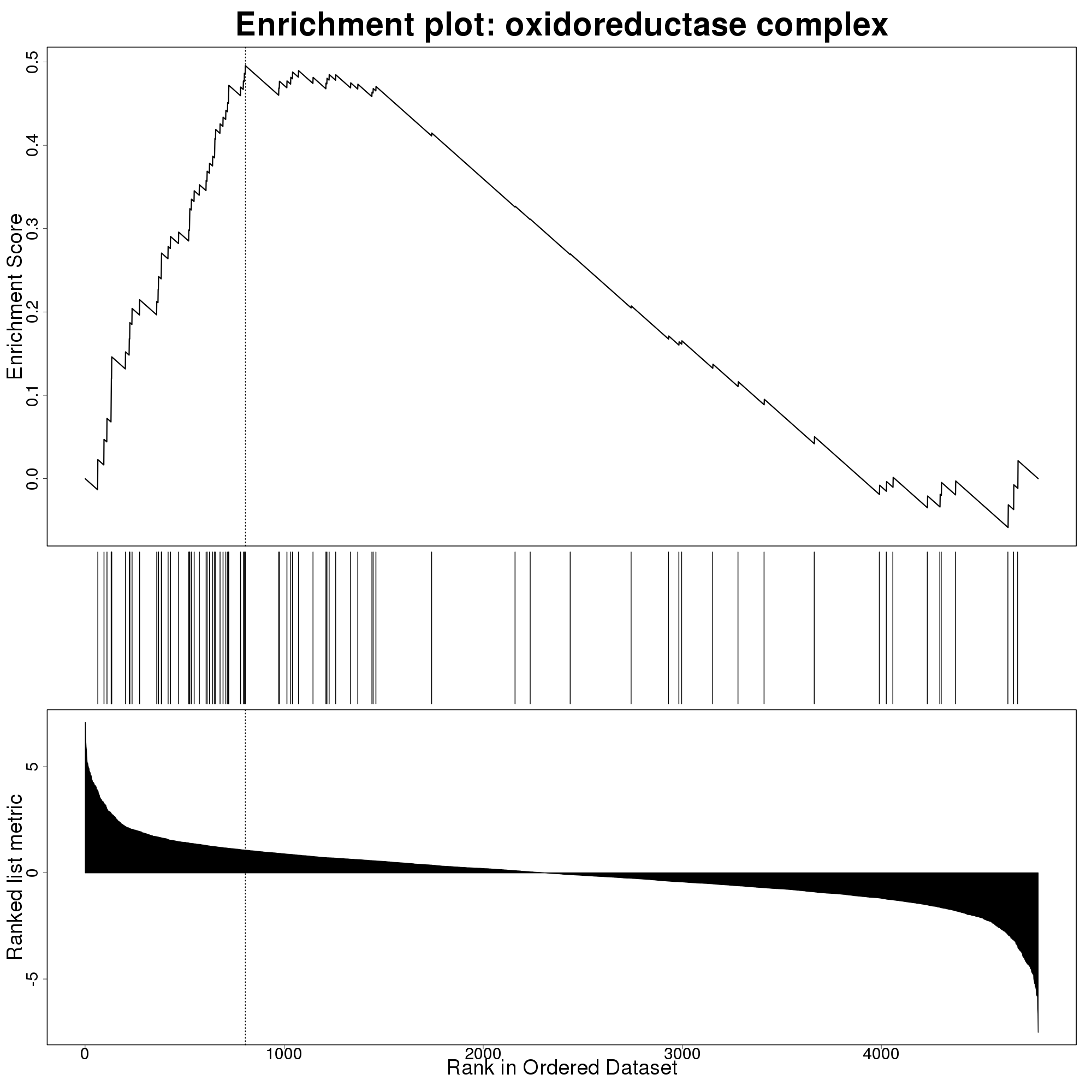

Supplement: Supplementary file 1 [file jcm-10-00407-s001.zip › sup/Supplementary_File_6/GSEA_Webgestalt/GSEA_GO_CC/Project_wg_result1604400208_GSEA/GO_1990204.png]

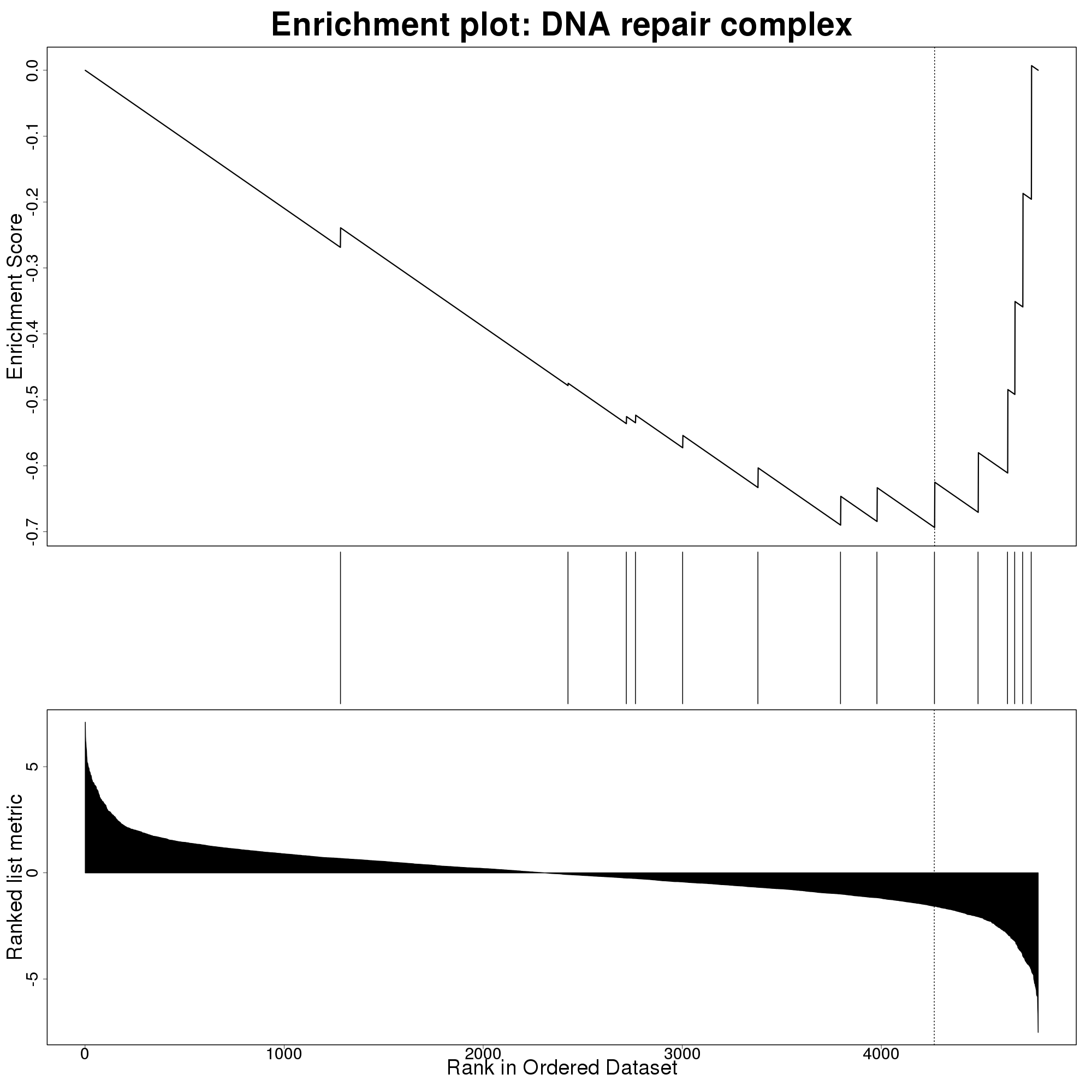

Supplement: Supplementary file 1 [file jcm-10-00407-s001.zip › sup/Supplementary_File_6/GSEA_Webgestalt/GSEA_GO_CC/Project_wg_result1604400208_GSEA/GO_1990391.png]

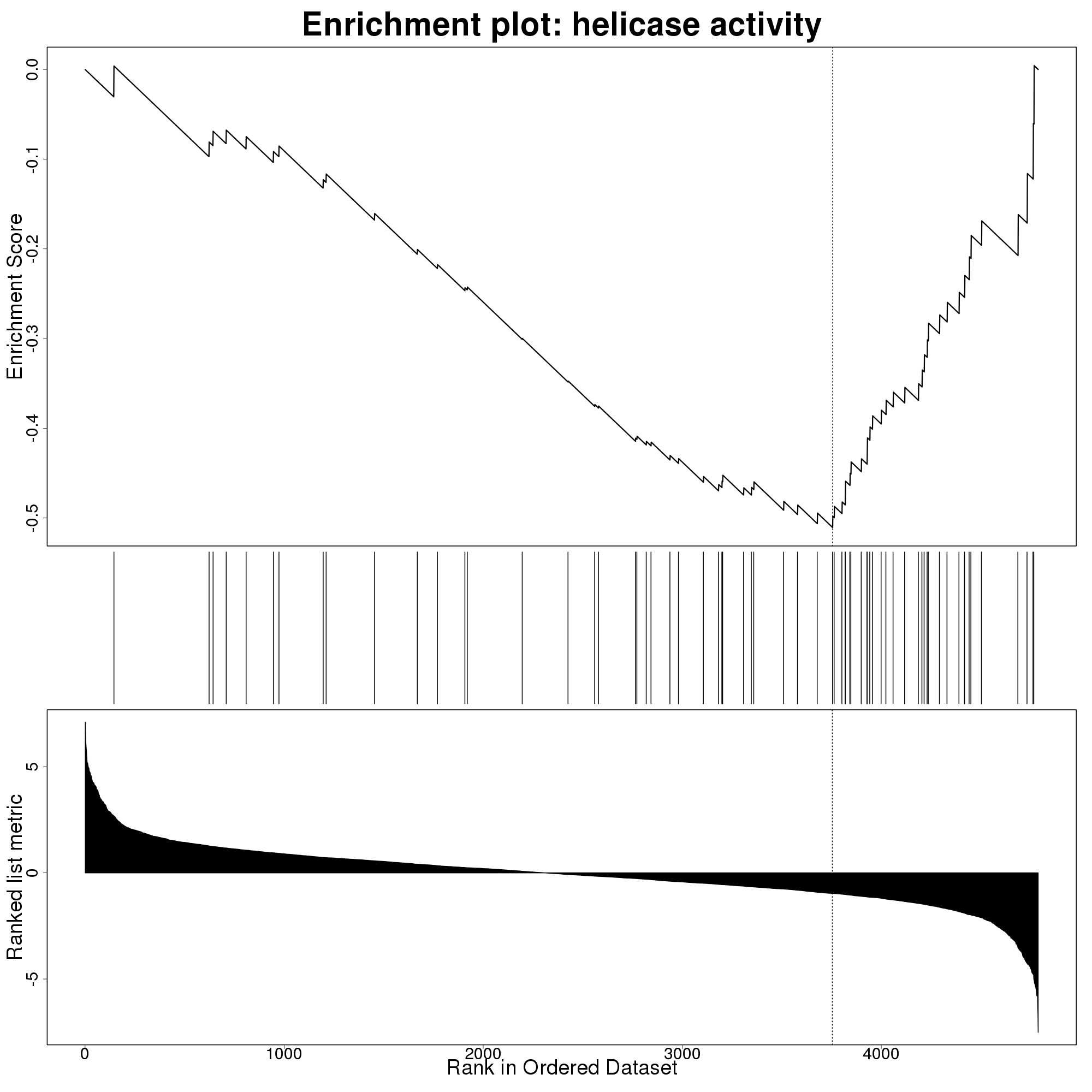

Supplement: Supplementary file 1 [file jcm-10-00407-s001.zip › sup/Supplementary_File_6/GSEA_Webgestalt/GSEA_GO_MF/Project_wg_result1604400218_GSEA/GO_0004386.png]

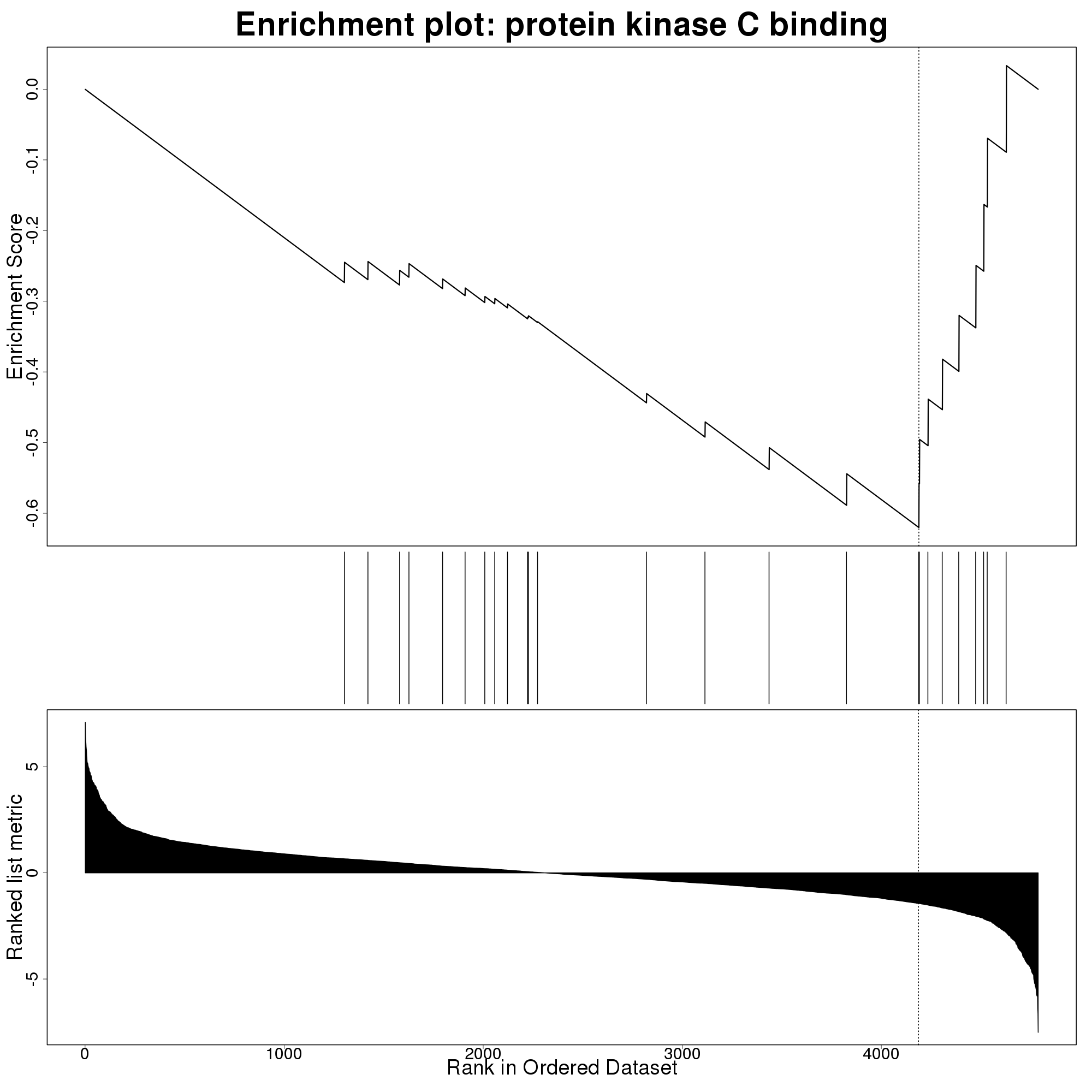

Supplement: Supplementary file 1 [file jcm-10-00407-s001.zip › sup/Supplementary_File_6/GSEA_Webgestalt/GSEA_GO_MF/Project_wg_result1604400218_GSEA/GO_0005080.png]

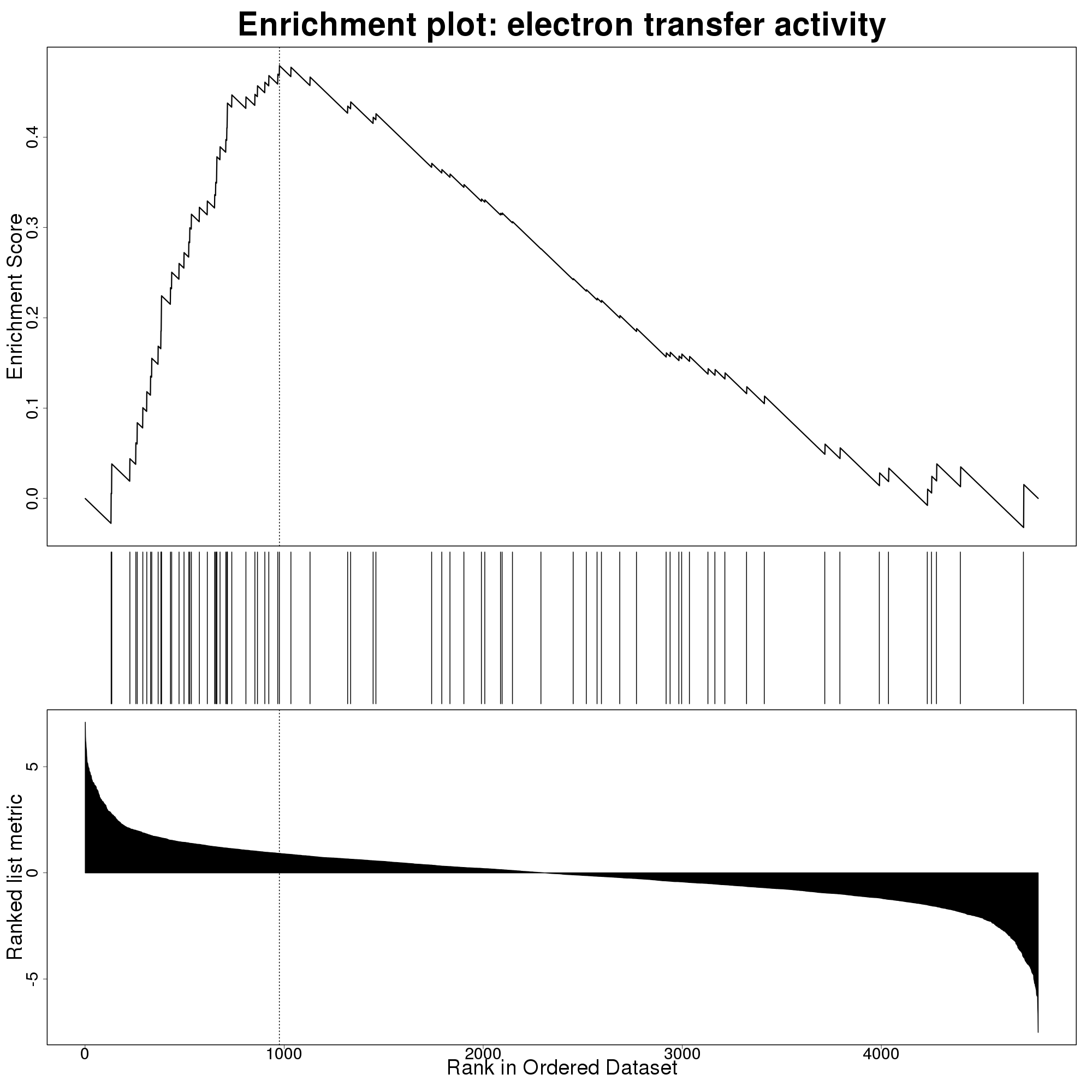

Supplement: Supplementary file 1 [file jcm-10-00407-s001.zip › sup/Supplementary_File_6/GSEA_Webgestalt/GSEA_GO_MF/Project_wg_result1604400218_GSEA/GO_0009055.png]

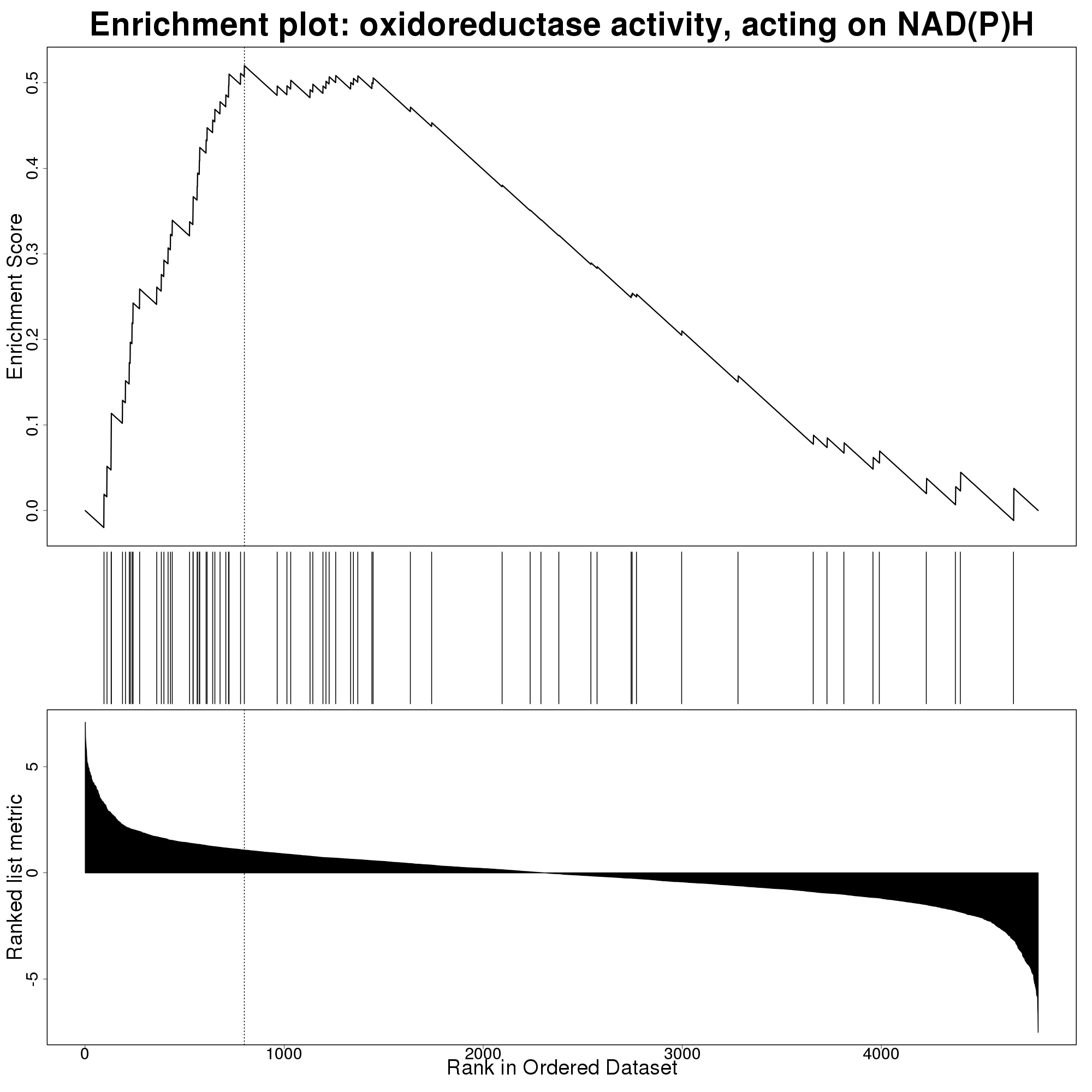

Supplement: Supplementary file 1 [file jcm-10-00407-s001.zip › sup/Supplementary_File_6/GSEA_Webgestalt/GSEA_GO_MF/Project_wg_result1604400218_GSEA/GO_0016651.png]

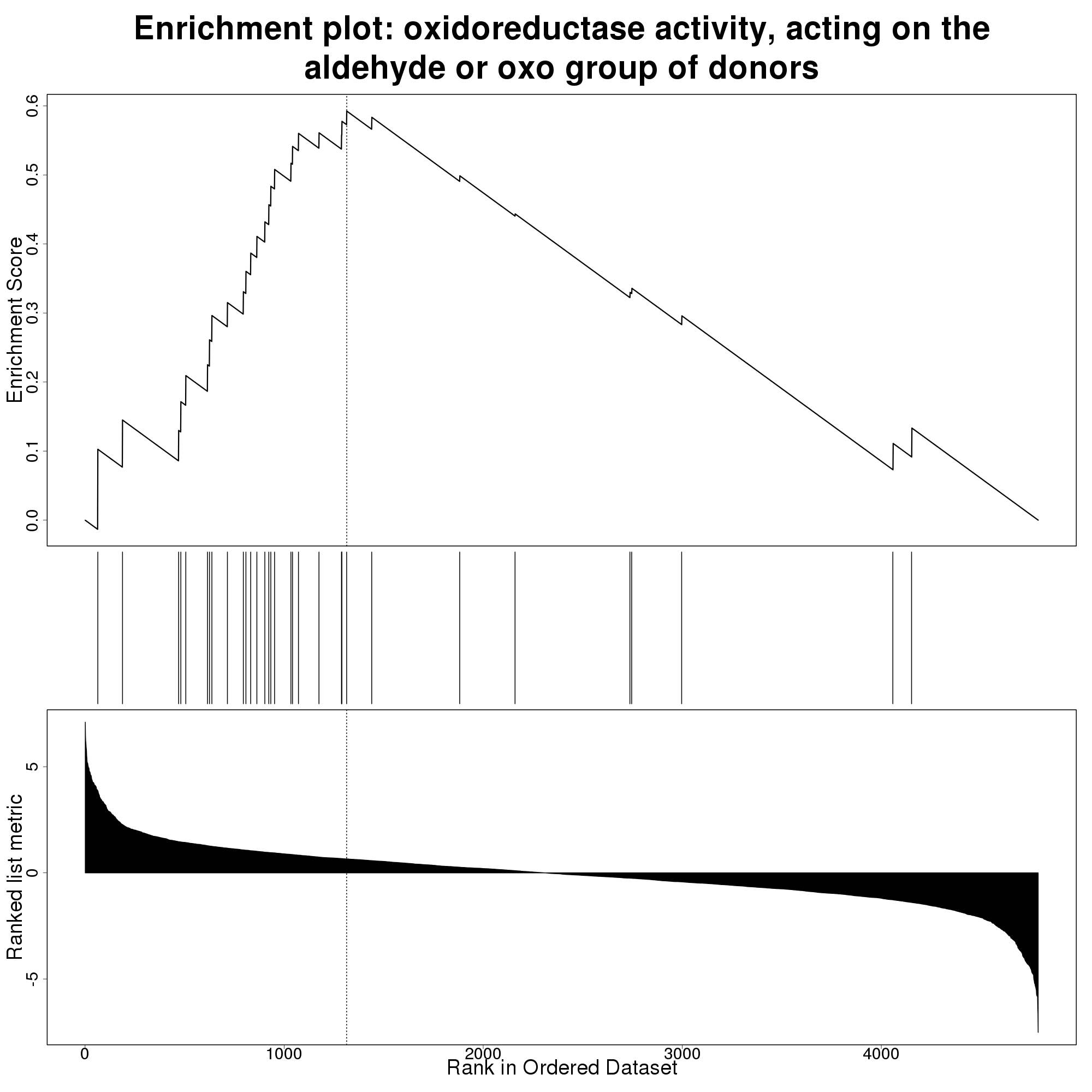

Supplement: Supplementary file 1 [file jcm-10-00407-s001.zip › sup/Supplementary_File_6/GSEA_Webgestalt/GSEA_GO_MF/Project_wg_result1604400218_GSEA/GO_0016903.png]

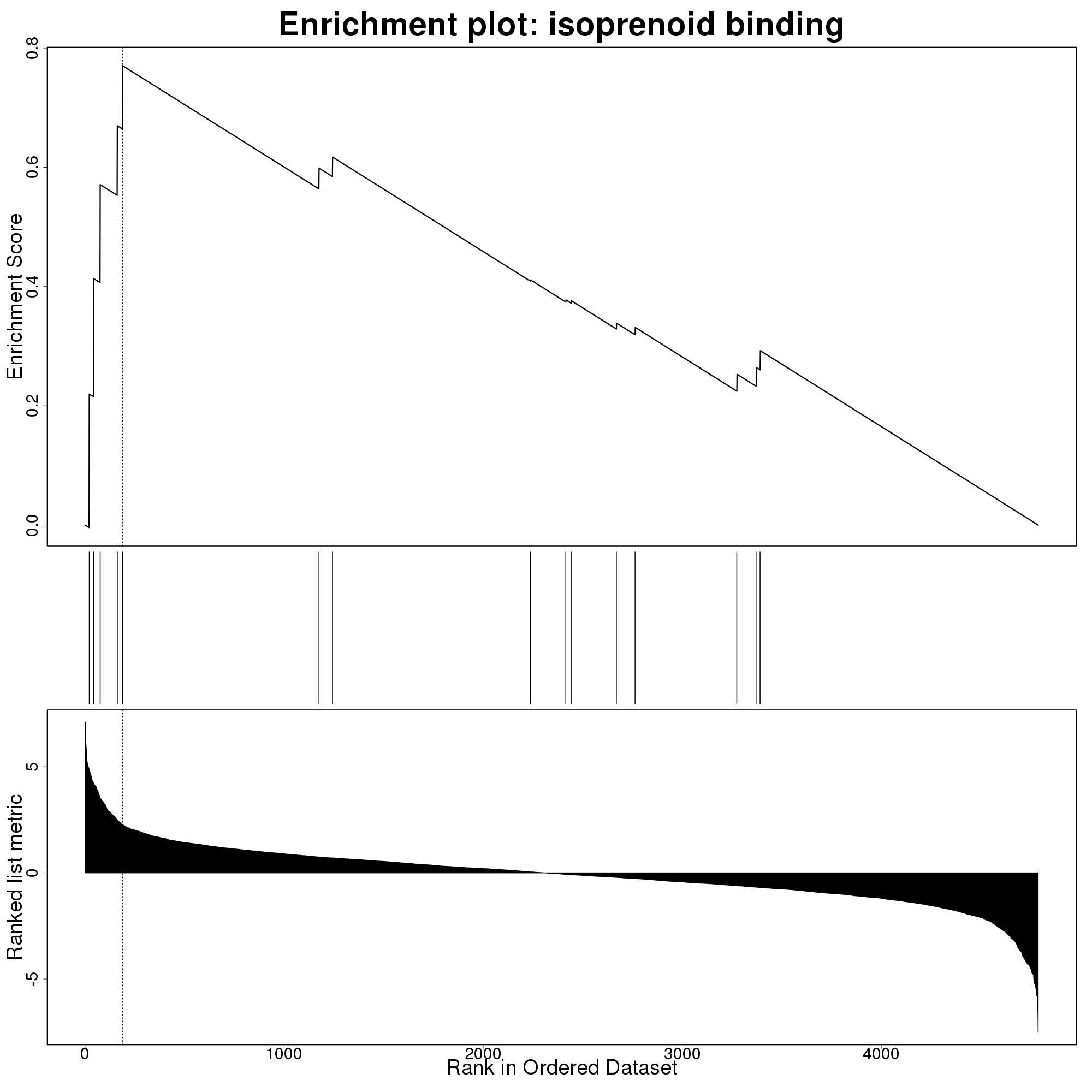

Supplement: Supplementary file 1 [file jcm-10-00407-s001.zip › sup/Supplementary_File_6/GSEA_Webgestalt/GSEA_GO_MF/Project_wg_result1604400218_GSEA/GO_0019840.png]

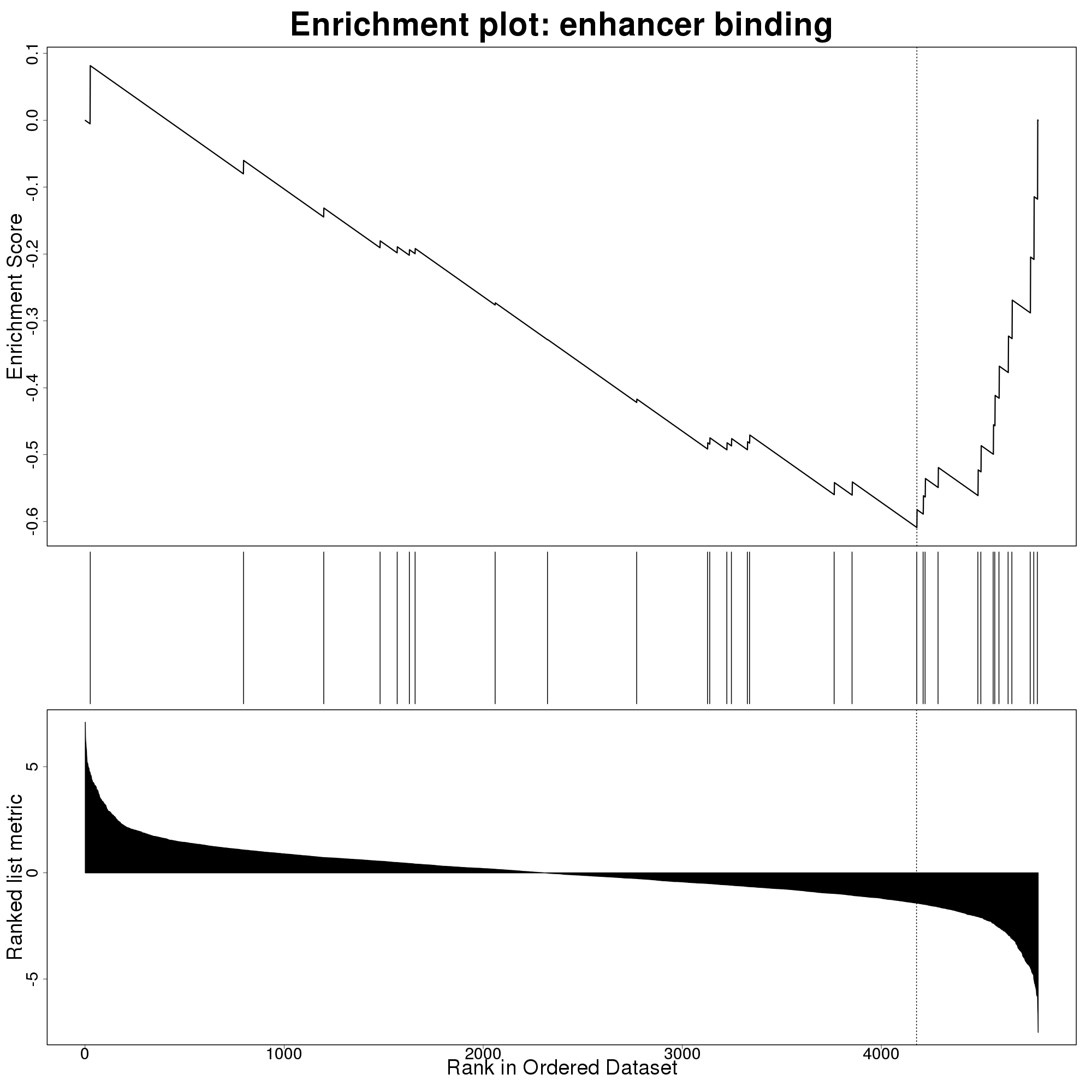

Supplement: Supplementary file 1 [file jcm-10-00407-s001.zip › sup/Supplementary_File_6/GSEA_Webgestalt/GSEA_GO_MF/Project_wg_result1604400218_GSEA/GO_0035326.png]

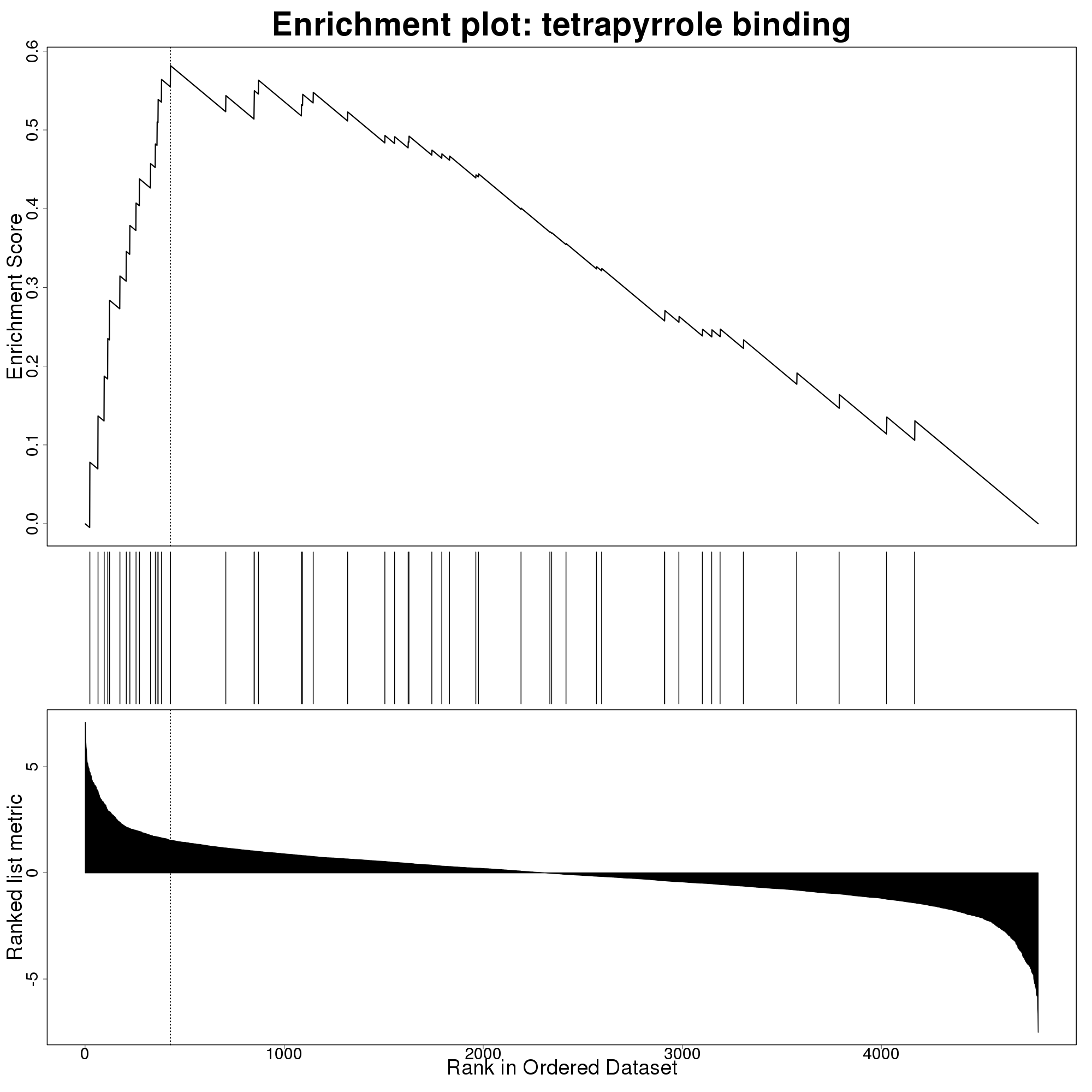

Supplement: Supplementary file 1 [file jcm-10-00407-s001.zip › sup/Supplementary_File_6/GSEA_Webgestalt/GSEA_GO_MF/Project_wg_result1604400218_GSEA/GO_0046906.png]

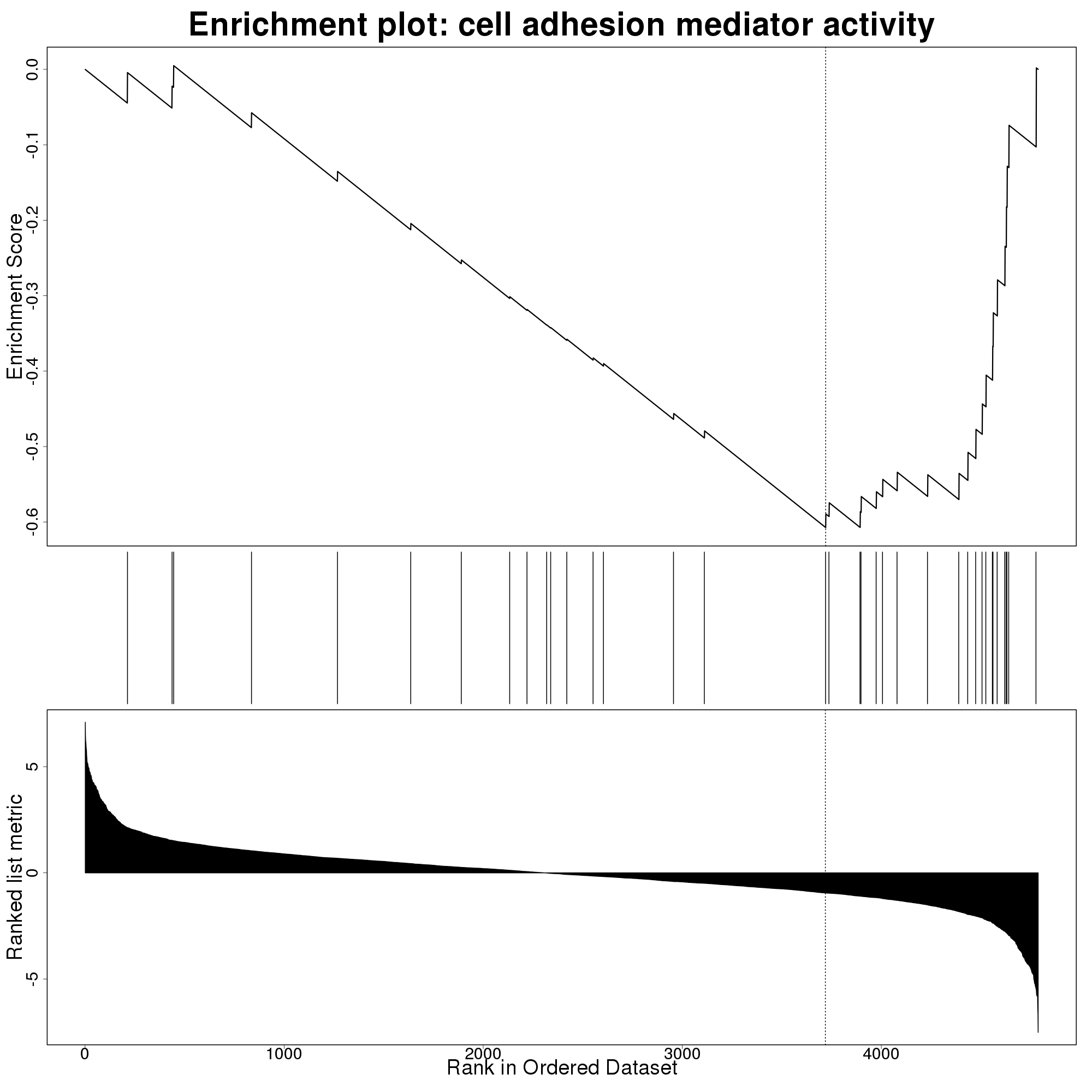

Supplement: Supplementary file 1 [file jcm-10-00407-s001.zip › sup/Supplementary_File_6/GSEA_Webgestalt/GSEA_GO_MF/Project_wg_result1604400218_GSEA/GO_0098631.png]

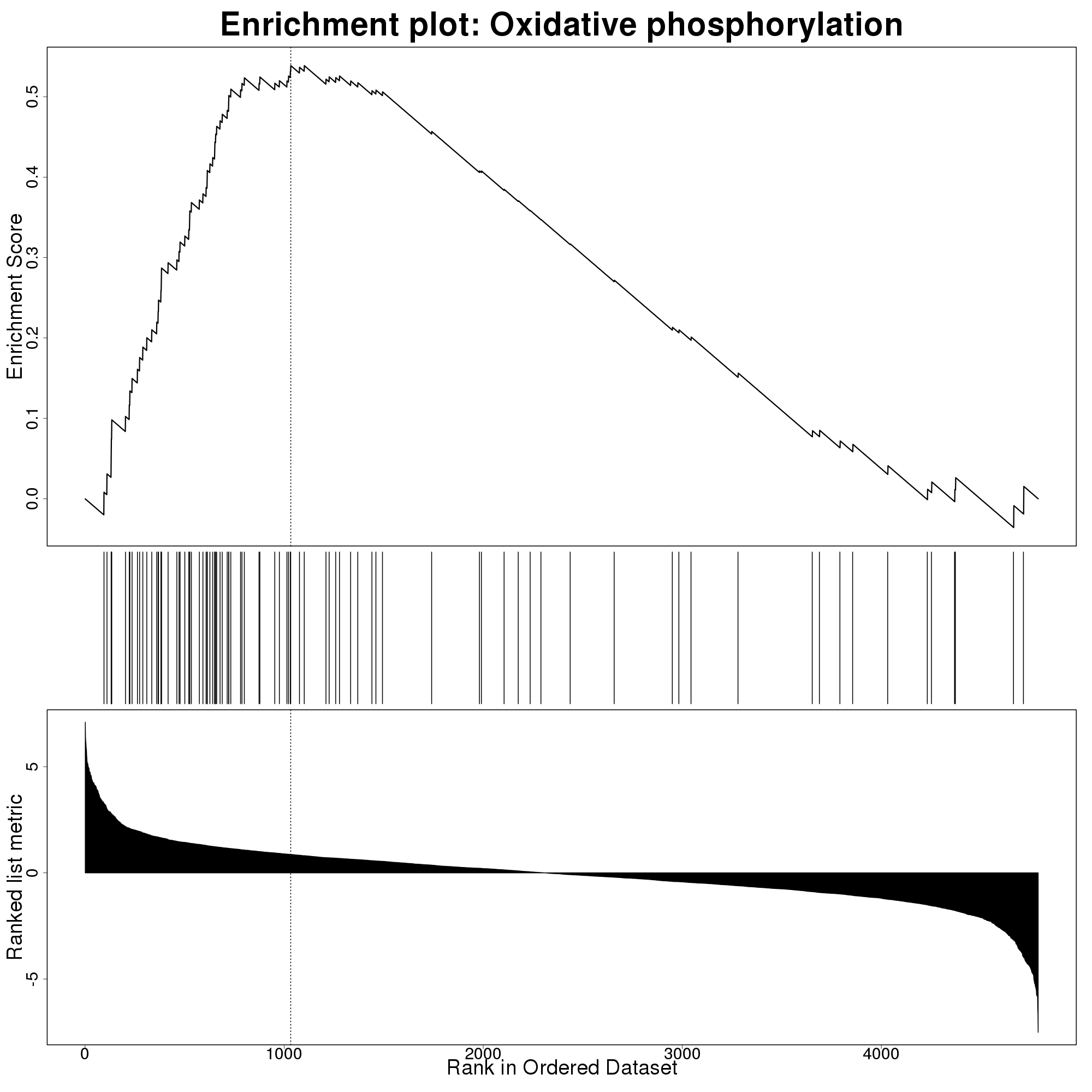

Supplement: Supplementary file 1 [file jcm-10-00407-s001.zip › sup/Supplementary_File_6/GSEA_Webgestalt/GSEA_Pathway_KEGG/Project_wg_result1604400229_GSEA/hsa00190.png]

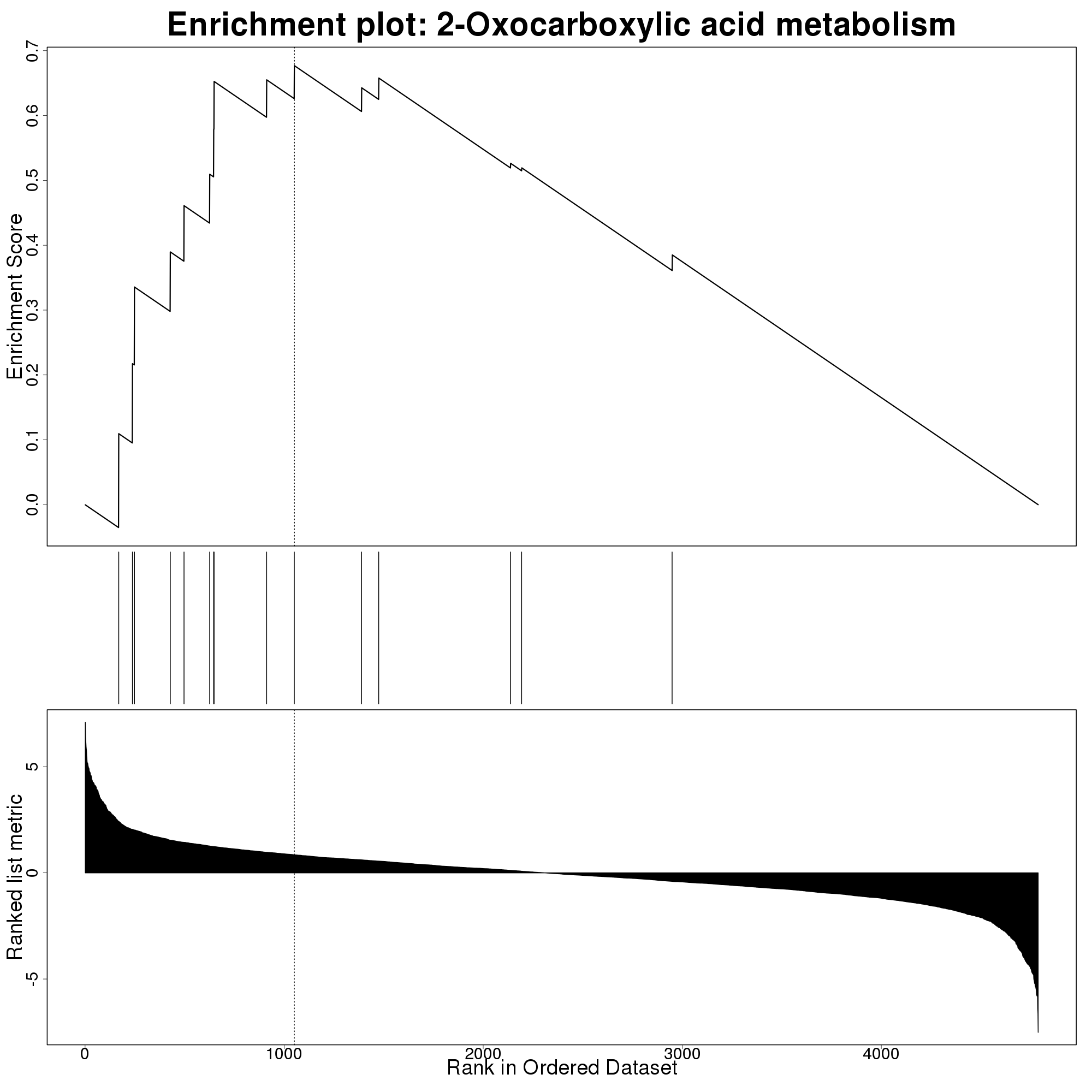

Supplement: Supplementary file 1 [file jcm-10-00407-s001.zip › sup/Supplementary_File_6/GSEA_Webgestalt/GSEA_Pathway_KEGG/Project_wg_result1604400229_GSEA/hsa01210.png]

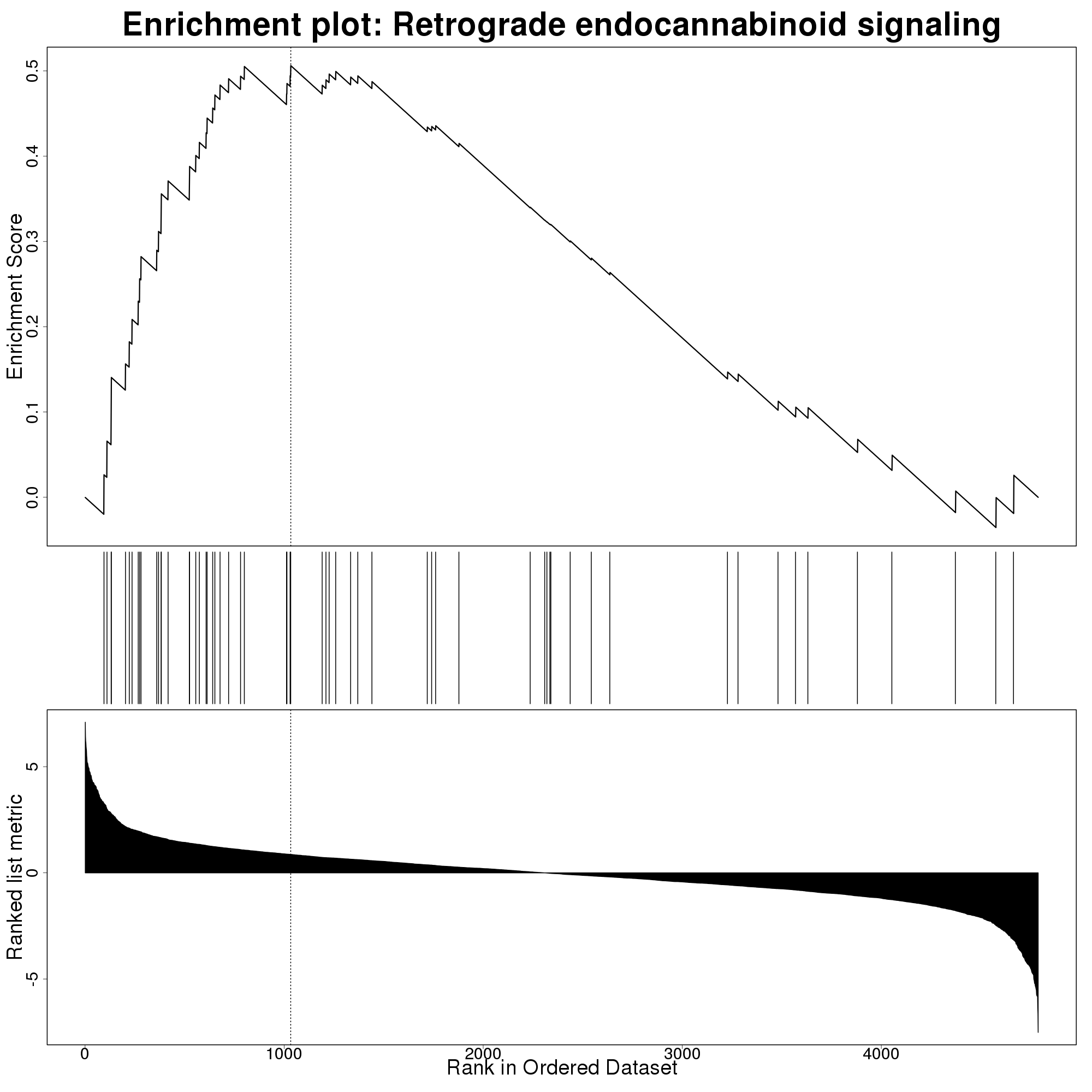

Supplement: Supplementary file 1 [file jcm-10-00407-s001.zip › sup/Supplementary_File_6/GSEA_Webgestalt/GSEA_Pathway_KEGG/Project_wg_result1604400229_GSEA/hsa04723.png]

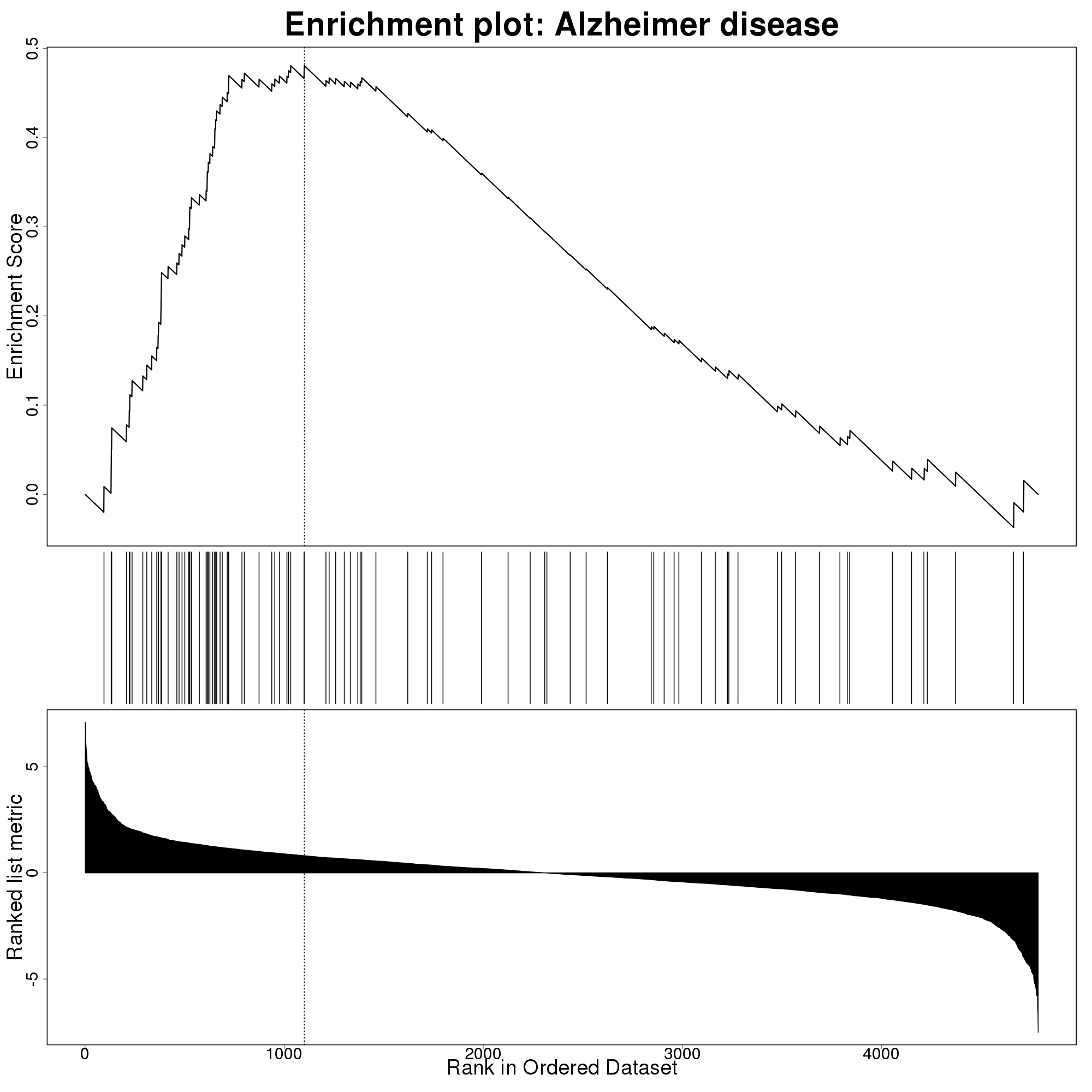

Supplement: Supplementary file 1 [file jcm-10-00407-s001.zip › sup/Supplementary_File_6/GSEA_Webgestalt/GSEA_Pathway_KEGG/Project_wg_result1604400229_GSEA/hsa05010.png]

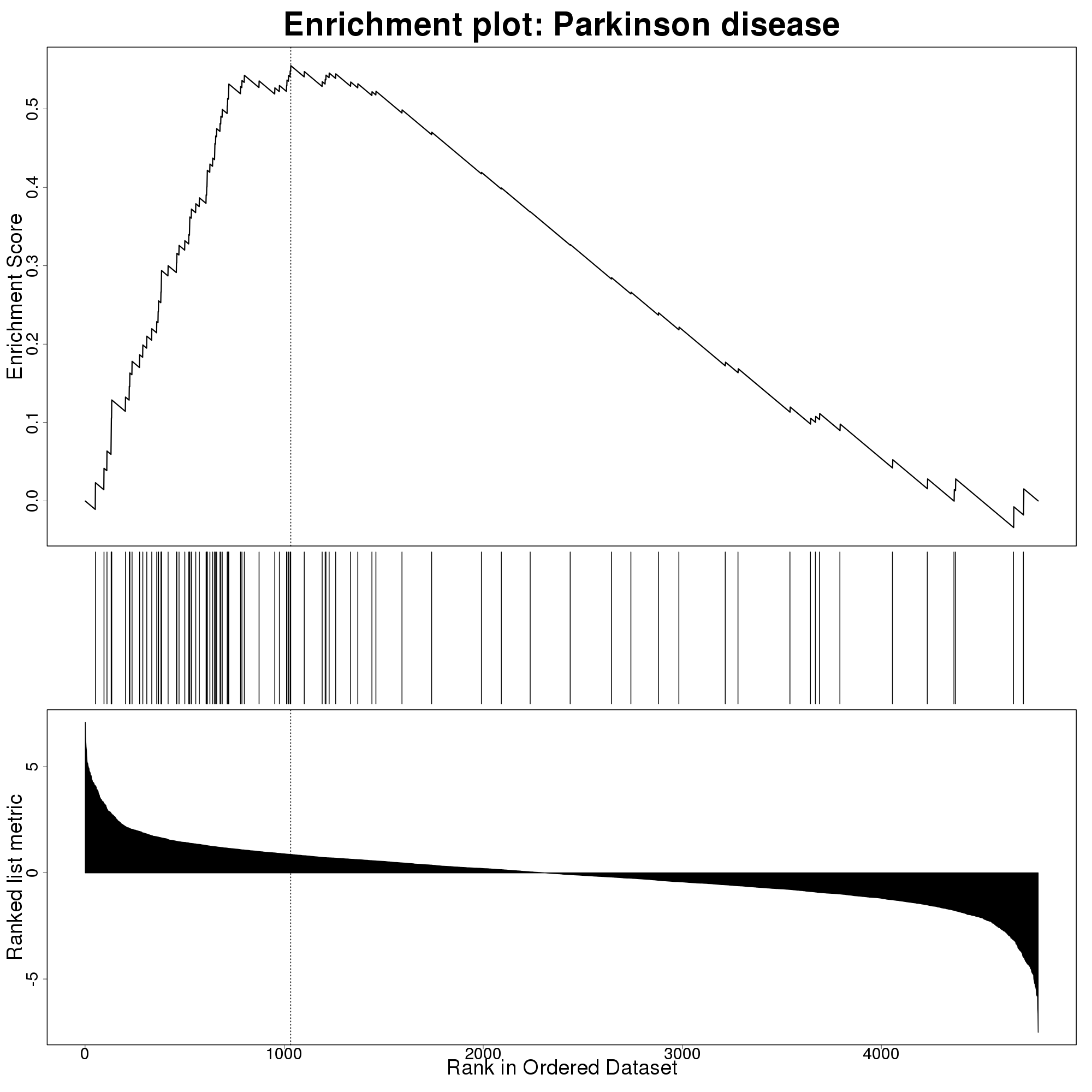

Supplement: Supplementary file 1 [file jcm-10-00407-s001.zip › sup/Supplementary_File_6/GSEA_Webgestalt/GSEA_Pathway_KEGG/Project_wg_result1604400229_GSEA/hsa05012.png]

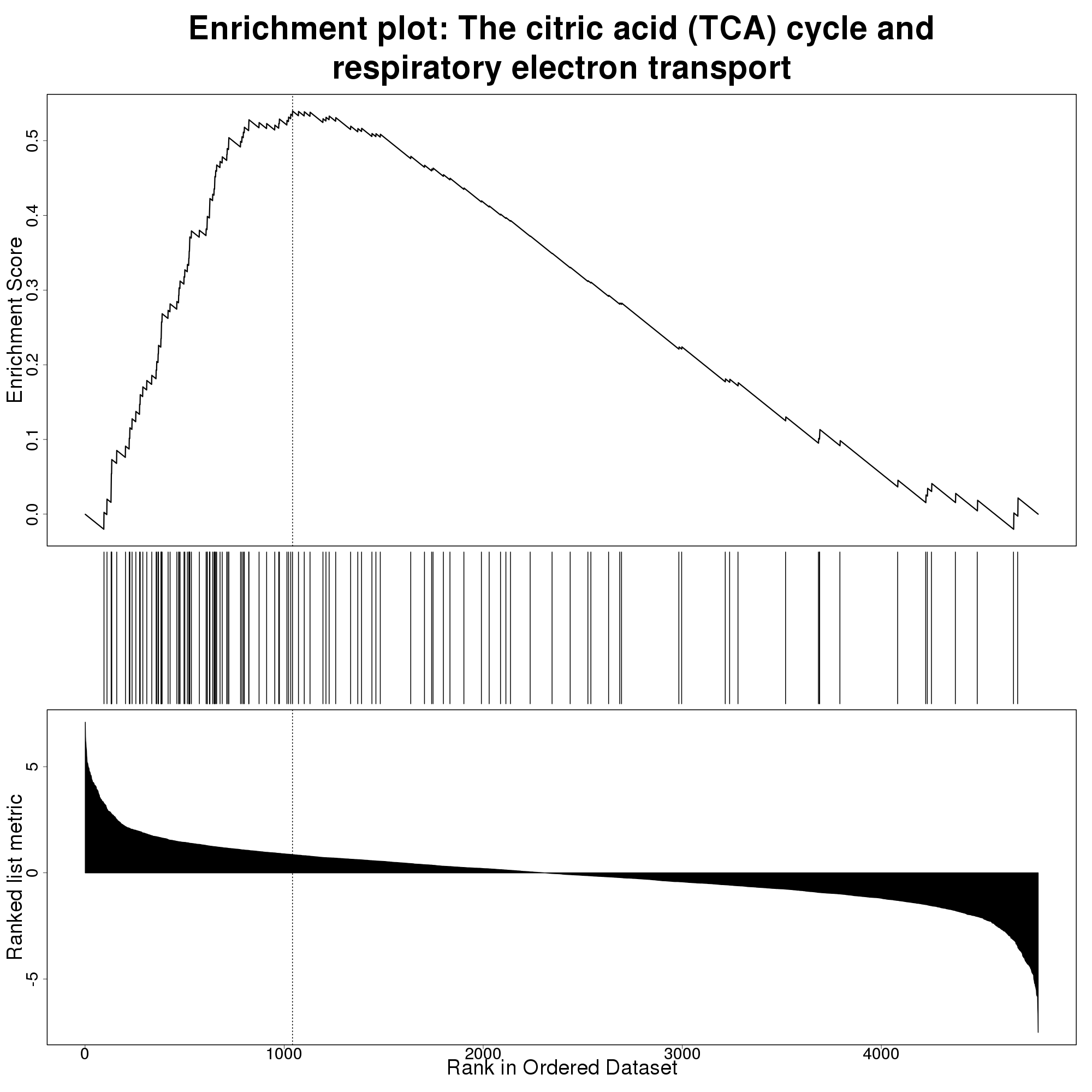

Supplement: Supplementary file 1 [file jcm-10-00407-s001.zip › sup/Supplementary_File_6/GSEA_Webgestalt/GSEA_Pathway_Reactome/Project_wg_result1604400246_GSEA/R_HSA_1428517.png]

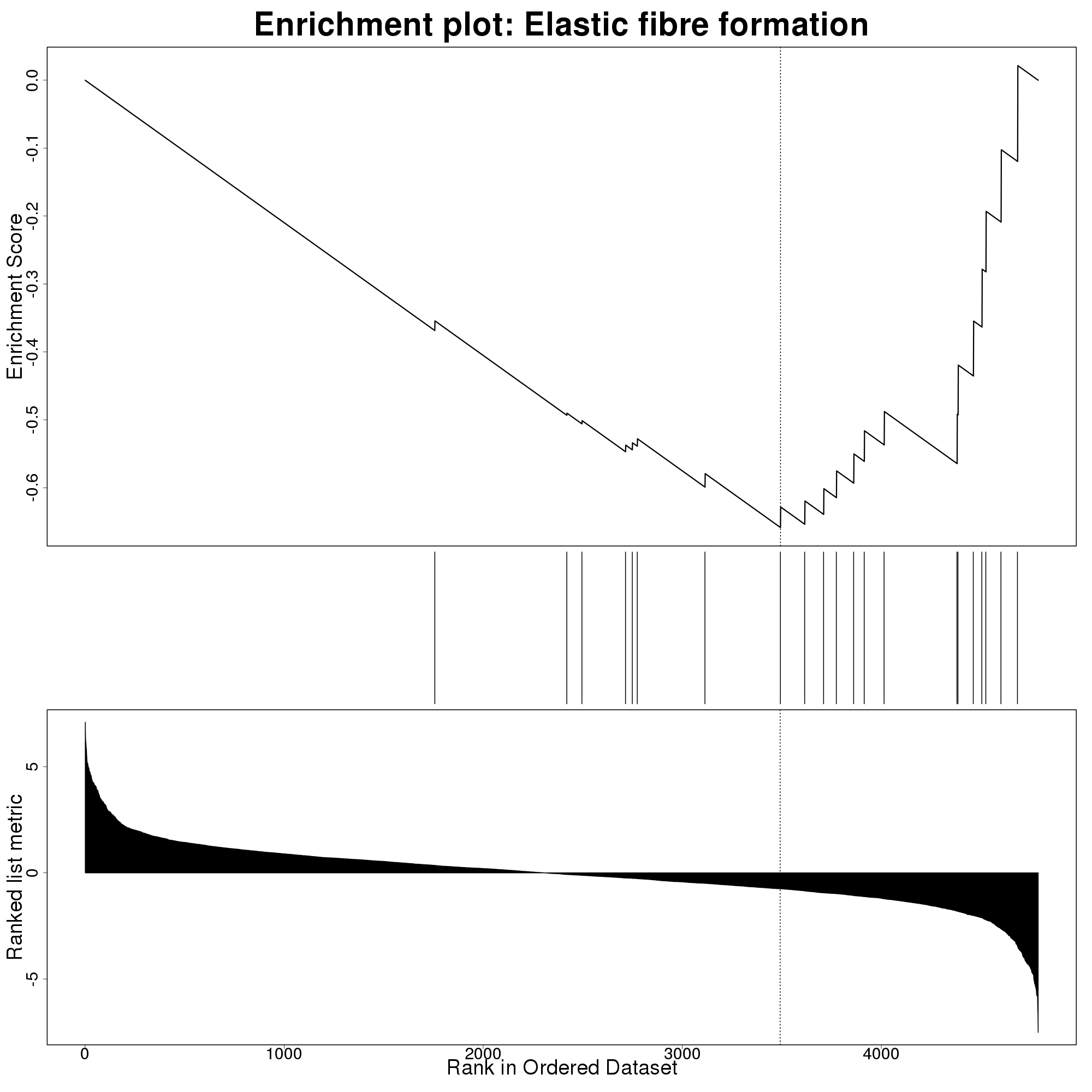

Supplement: Supplementary file 1 [file jcm-10-00407-s001.zip › sup/Supplementary_File_6/GSEA_Webgestalt/GSEA_Pathway_Reactome/Project_wg_result1604400246_GSEA/R_HSA_1566948.png]

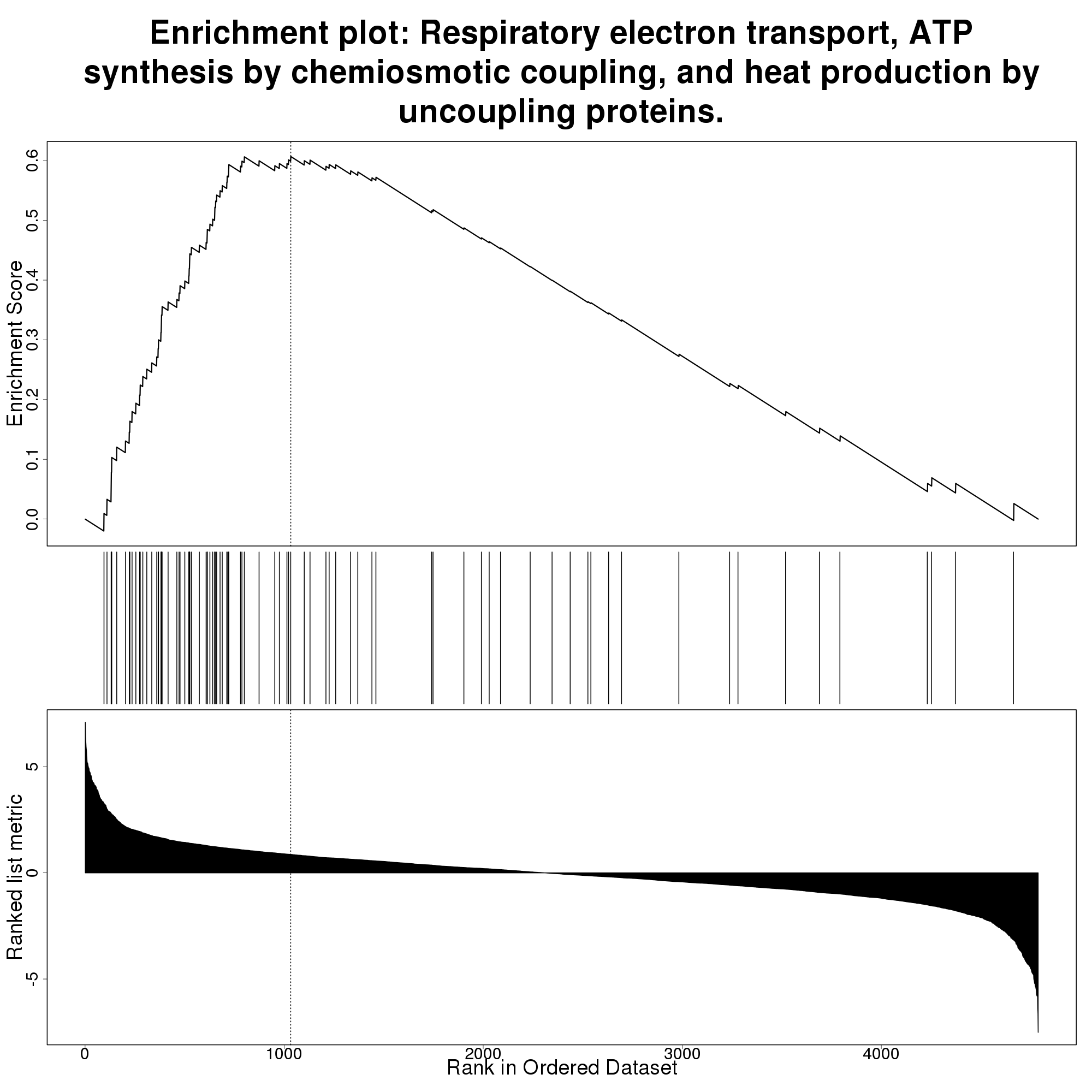

Supplement: Supplementary file 1 [file jcm-10-00407-s001.zip › sup/Supplementary_File_6/GSEA_Webgestalt/GSEA_Pathway_Reactome/Project_wg_result1604400246_GSEA/R_HSA_163200.png]

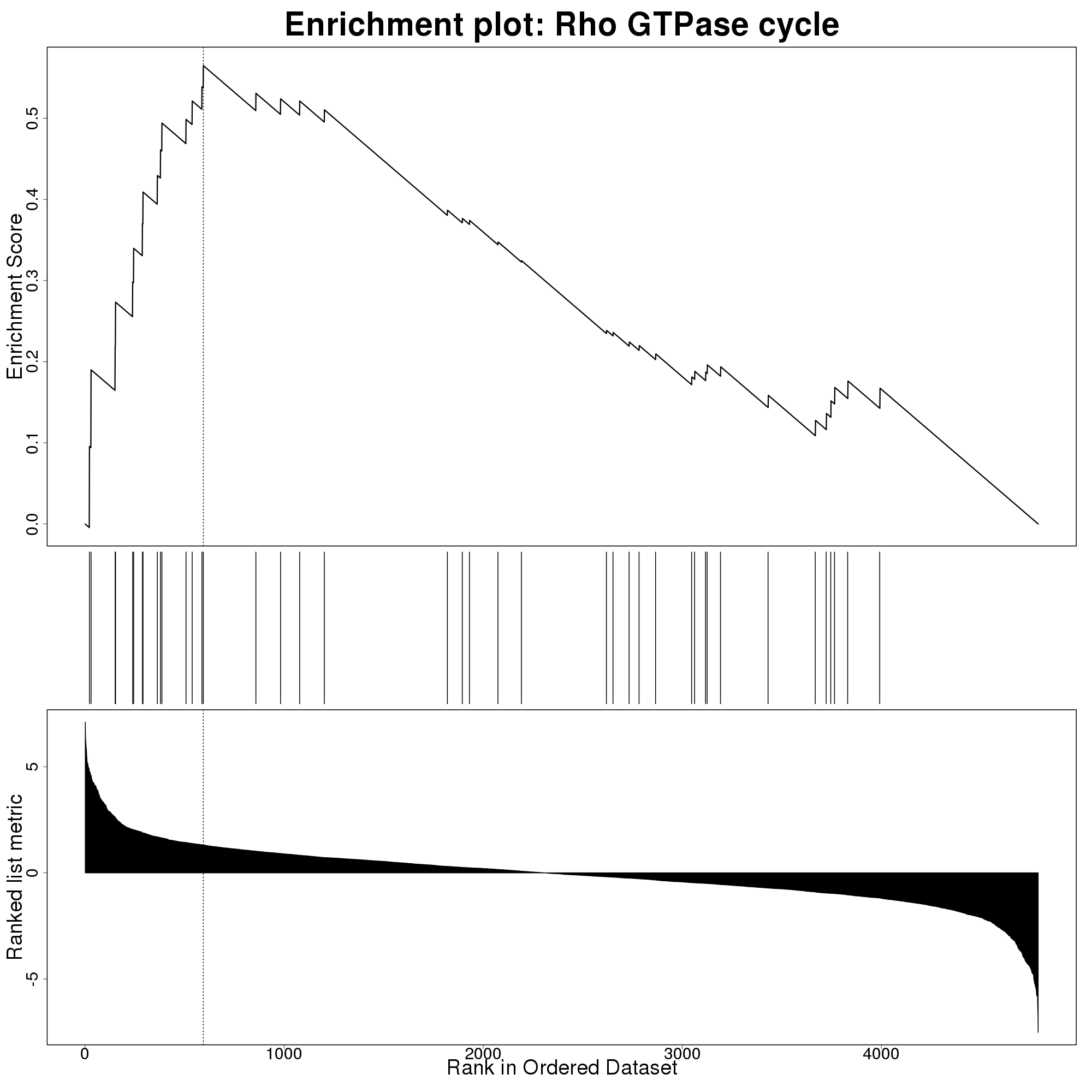

Supplement: Supplementary file 1 [file jcm-10-00407-s001.zip › sup/Supplementary_File_6/GSEA_Webgestalt/GSEA_Pathway_Reactome/Project_wg_result1604400246_GSEA/R_HSA_194840.png]

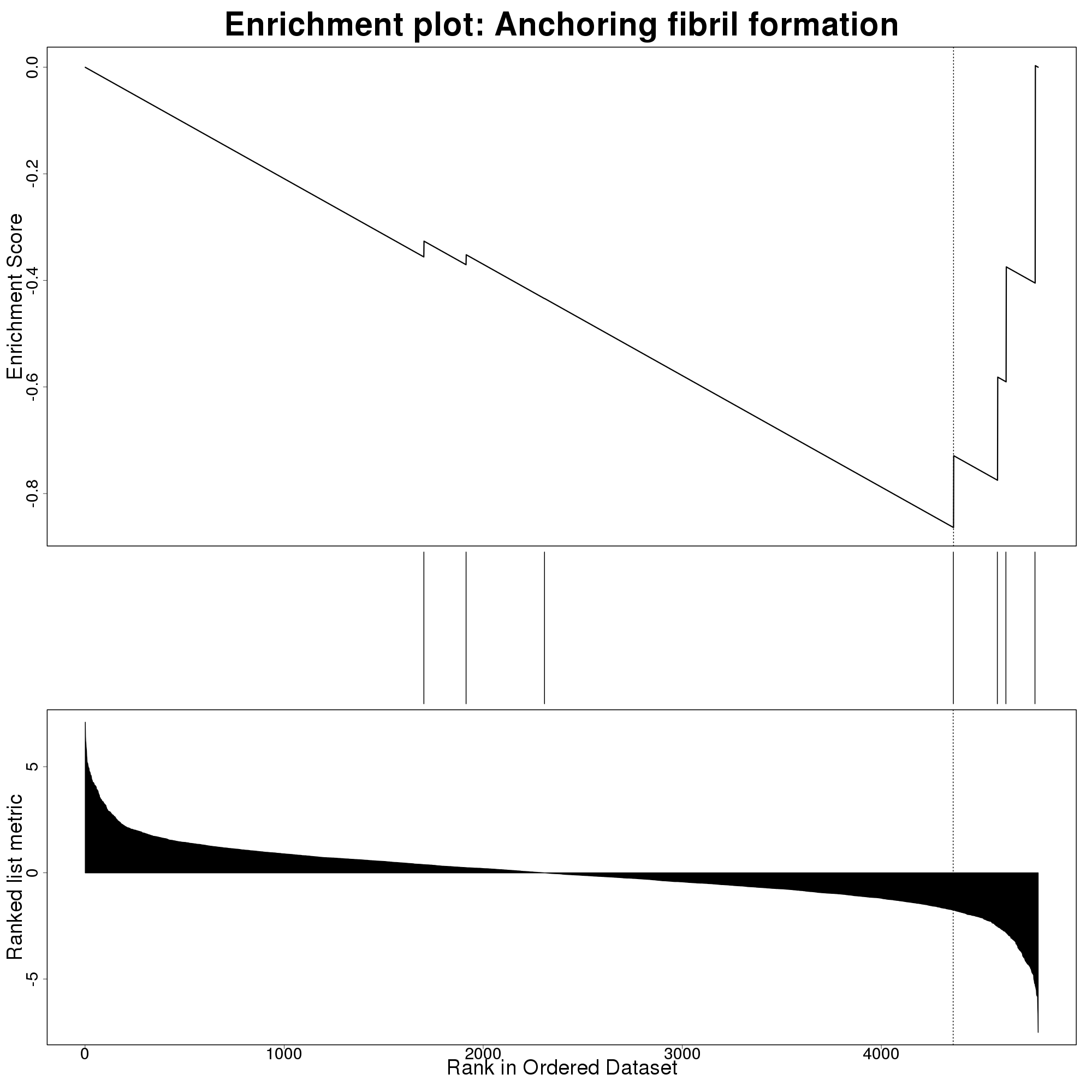

Supplement: Supplementary file 1 [file jcm-10-00407-s001.zip › sup/Supplementary_File_6/GSEA_Webgestalt/GSEA_Pathway_Reactome/Project_wg_result1604400246_GSEA/R_HSA_2214320.png]

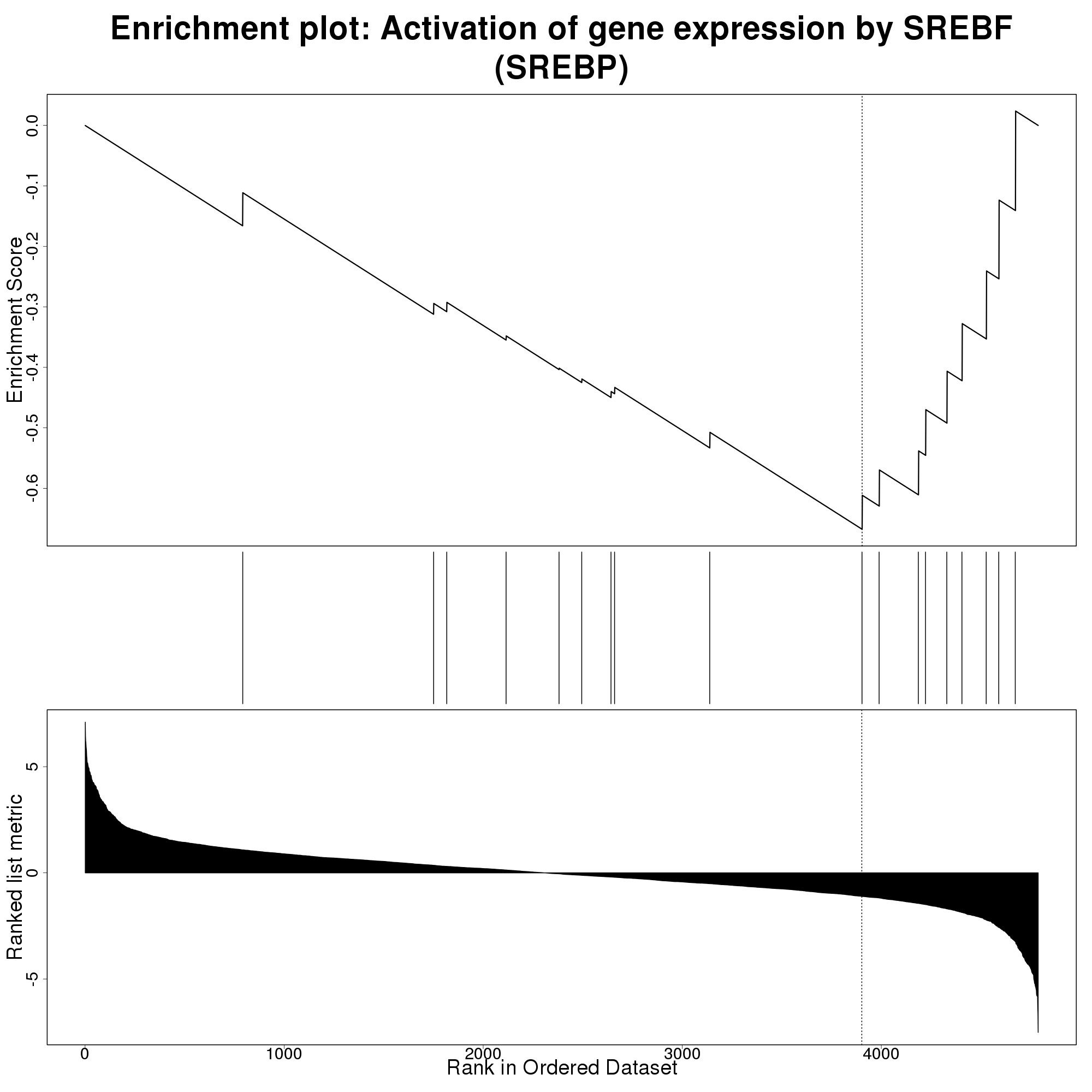

Supplement: Supplementary file 1 [file jcm-10-00407-s001.zip › sup/Supplementary_File_6/GSEA_Webgestalt/GSEA_Pathway_Reactome/Project_wg_result1604400246_GSEA/R_HSA_2426168.png]

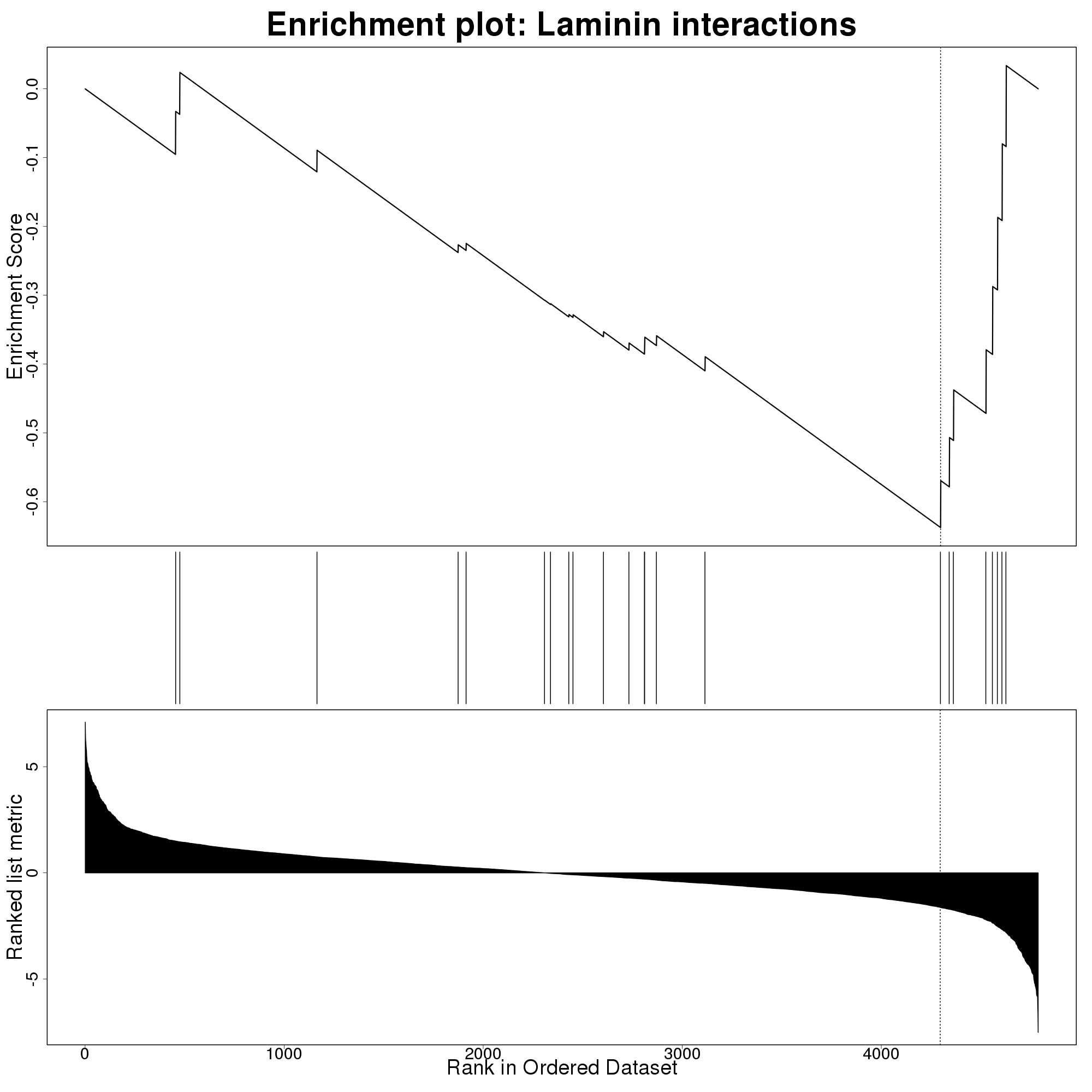

Supplement: Supplementary file 1 [file jcm-10-00407-s001.zip › sup/Supplementary_File_6/GSEA_Webgestalt/GSEA_Pathway_Reactome/Project_wg_result1604400246_GSEA/R_HSA_3000157.png]

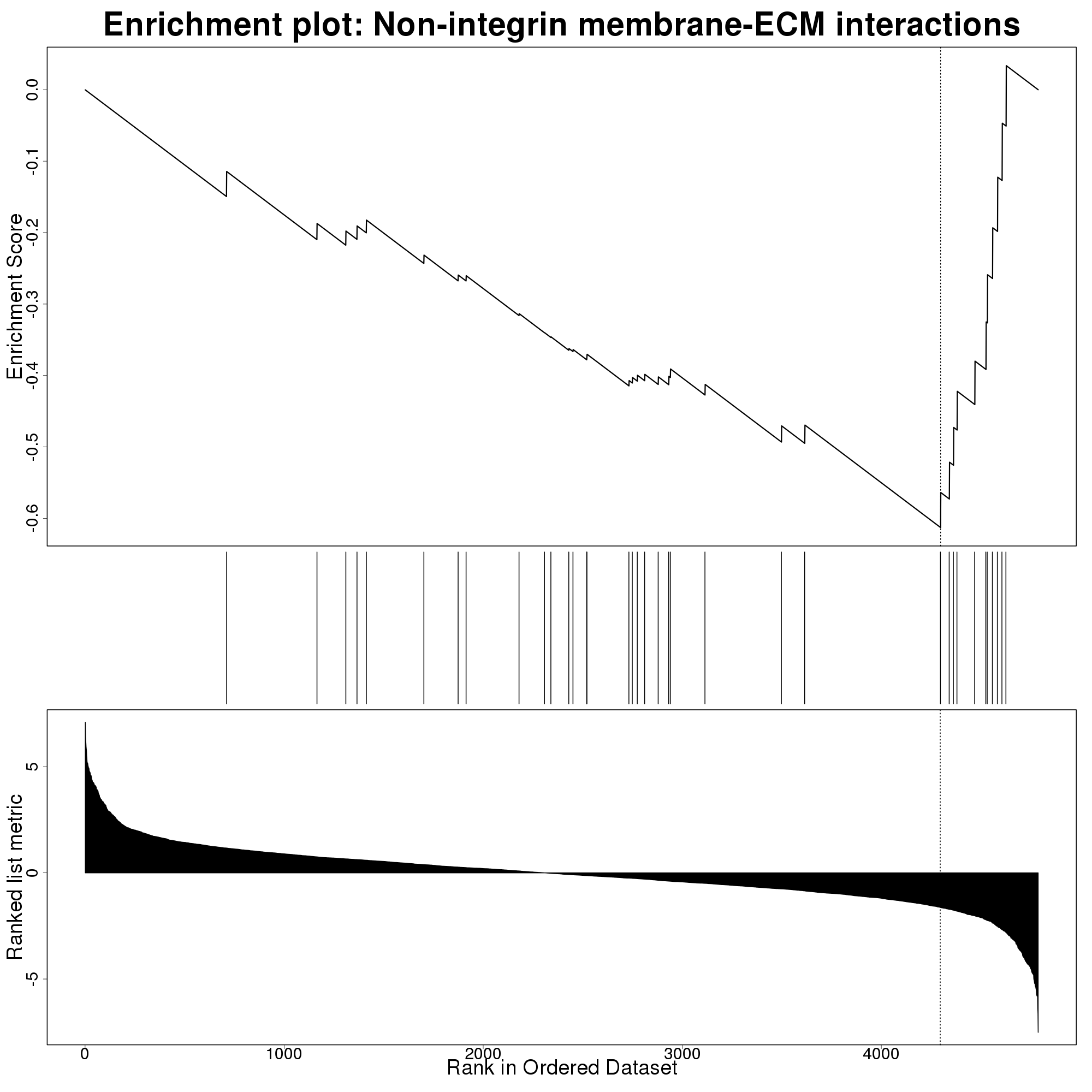

Supplement: Supplementary file 1 [file jcm-10-00407-s001.zip › sup/Supplementary_File_6/GSEA_Webgestalt/GSEA_Pathway_Reactome/Project_wg_result1604400246_GSEA/R_HSA_3000171.png]

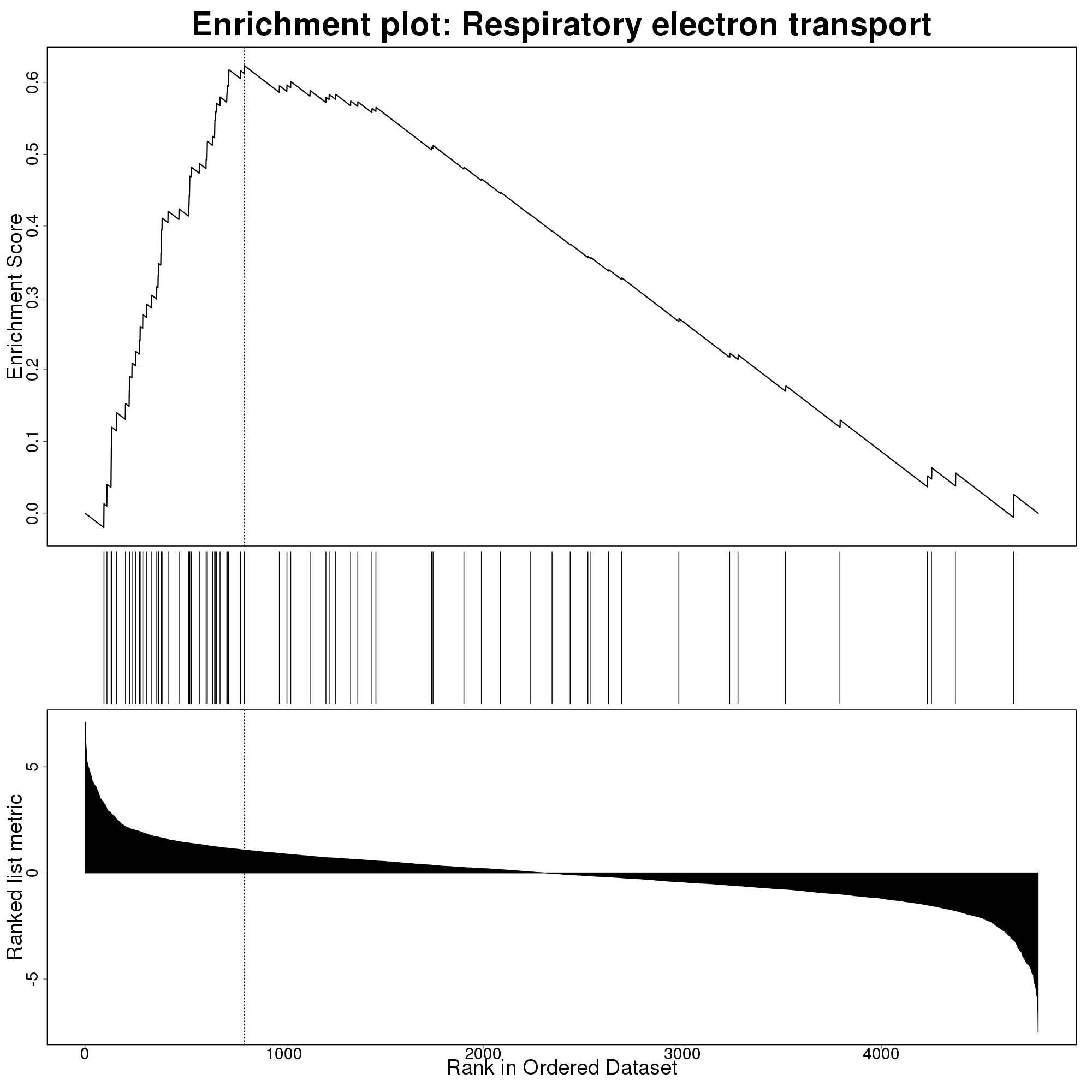

Supplement: Supplementary file 1 [file jcm-10-00407-s001.zip › sup/Supplementary_File_6/GSEA_Webgestalt/GSEA_Pathway_Reactome/Project_wg_result1604400246_GSEA/R_HSA_611105.png]

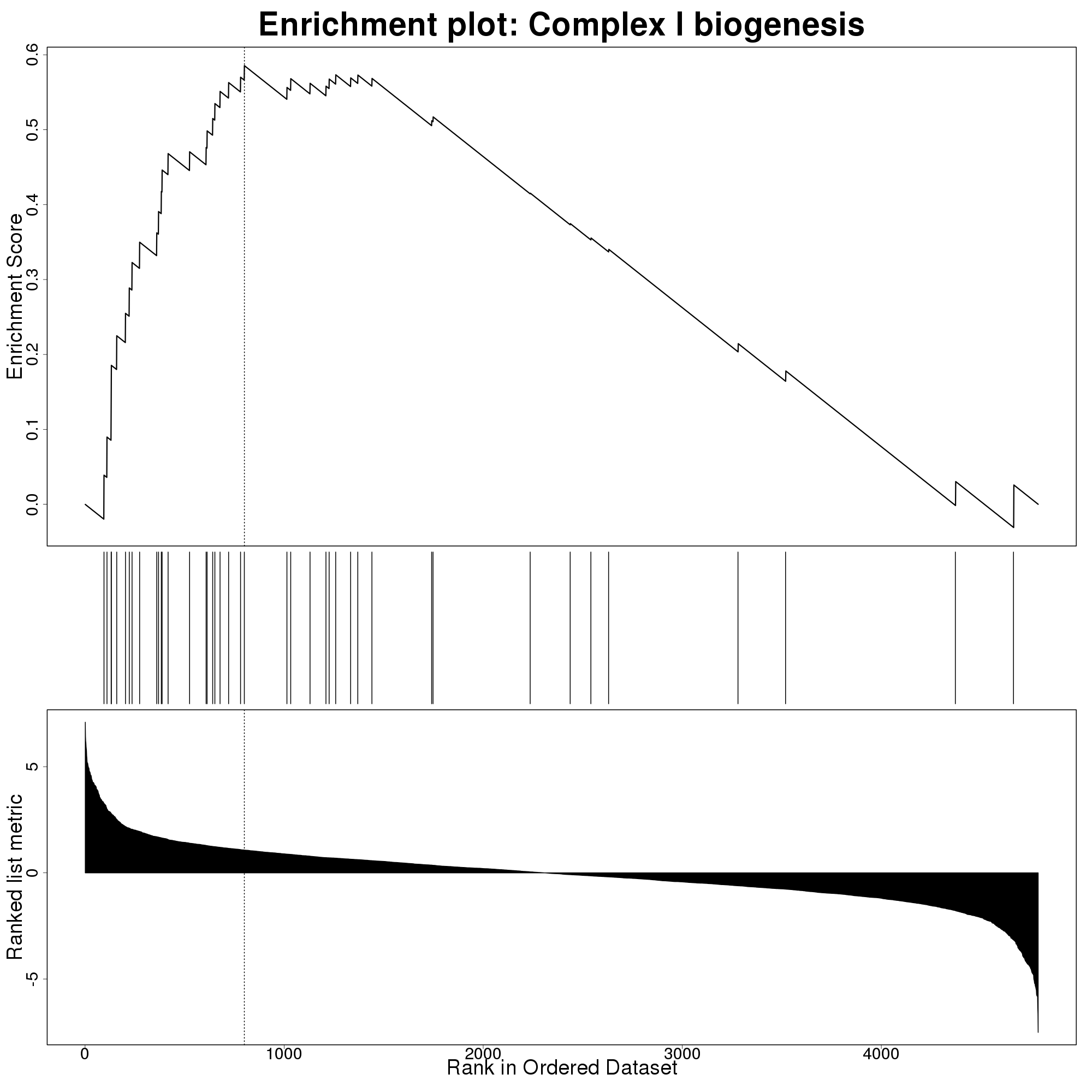

Supplement: Supplementary file 1 [file jcm-10-00407-s001.zip › sup/Supplementary_File_6/GSEA_Webgestalt/GSEA_Pathway_Reactome/Project_wg_result1604400246_GSEA/R_HSA_6799198.png]

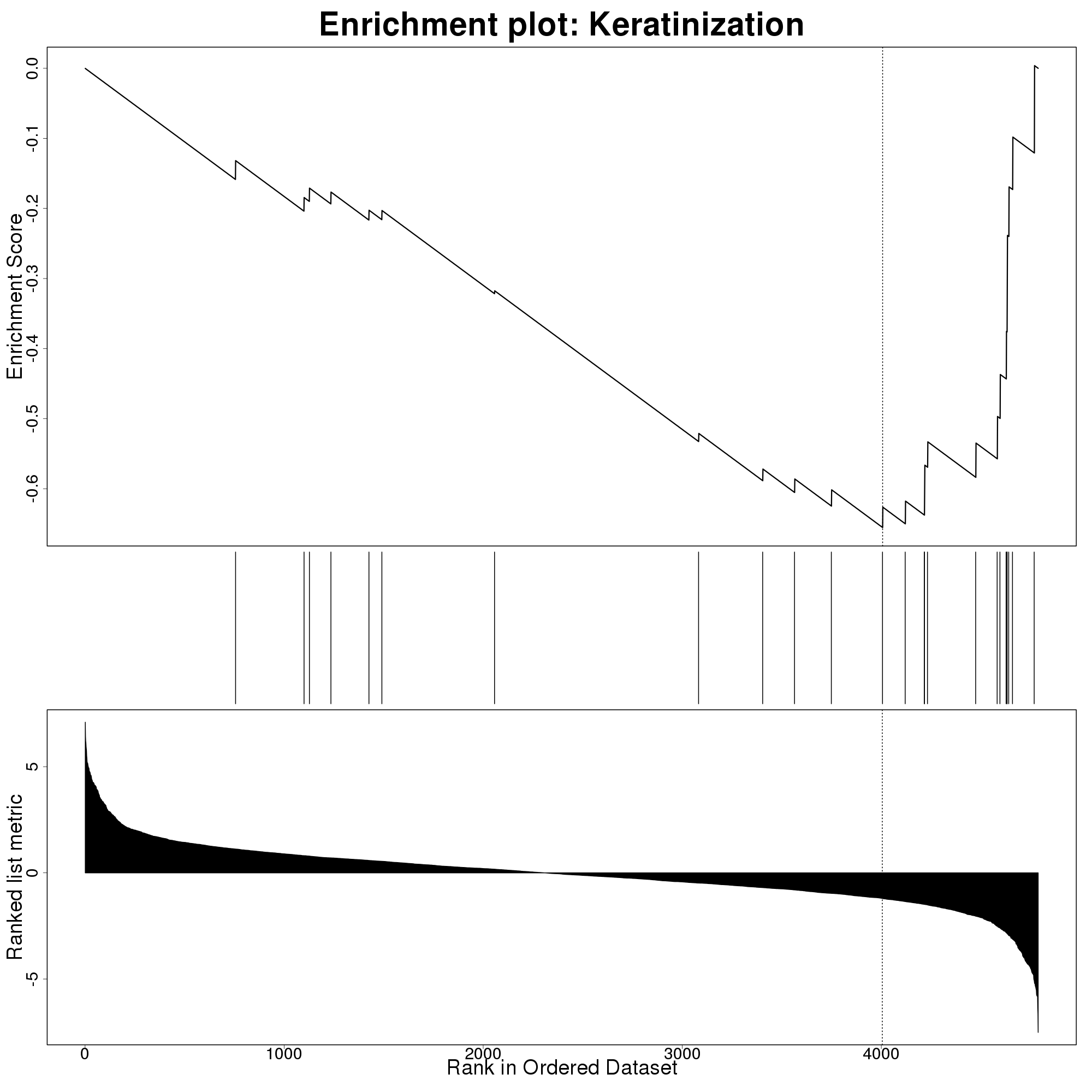

Supplement: Supplementary file 1 [file jcm-10-00407-s001.zip › sup/Supplementary_File_6/GSEA_Webgestalt/GSEA_Pathway_Reactome/Project_wg_result1604400246_GSEA/R_HSA_6805567.png]

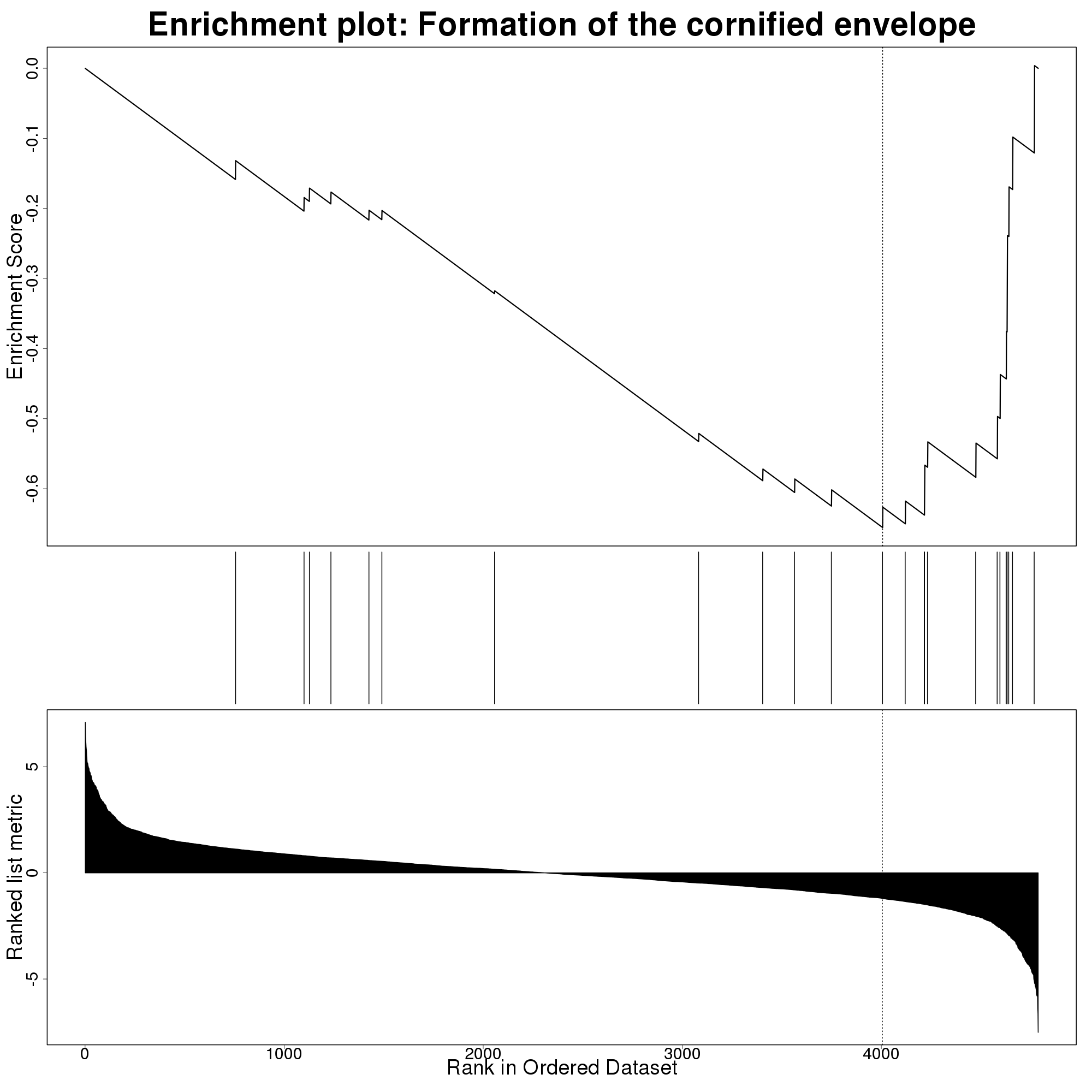

Supplement: Supplementary file 1 [file jcm-10-00407-s001.zip › sup/Supplementary_File_6/GSEA_Webgestalt/GSEA_Pathway_Reactome/Project_wg_result1604400246_GSEA/R_HSA_6809371.png]

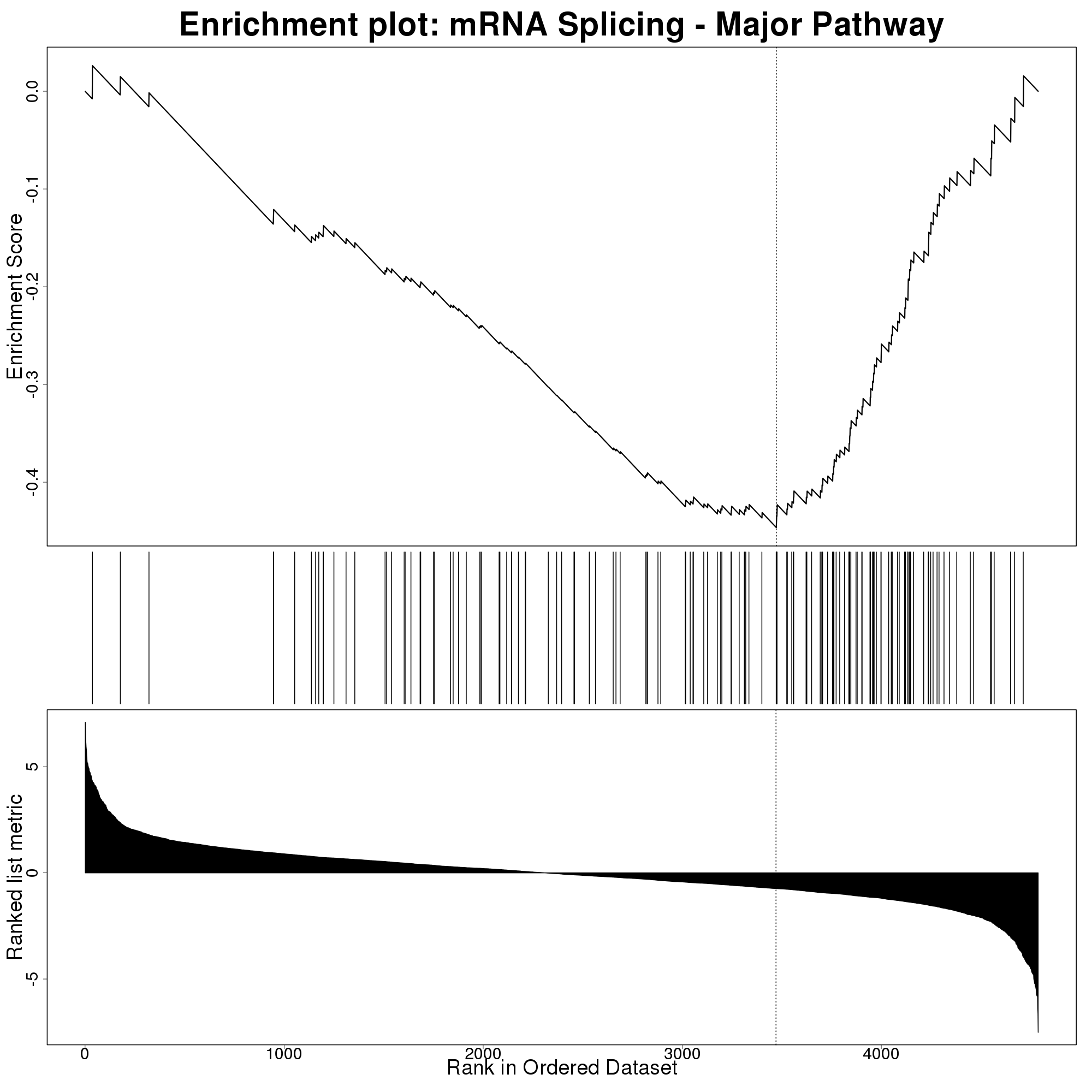

Supplement: Supplementary file 1 [file jcm-10-00407-s001.zip › sup/Supplementary_File_6/GSEA_Webgestalt/GSEA_Pathway_Reactome/Project_wg_result1604400246_GSEA/R_HSA_72163.png]

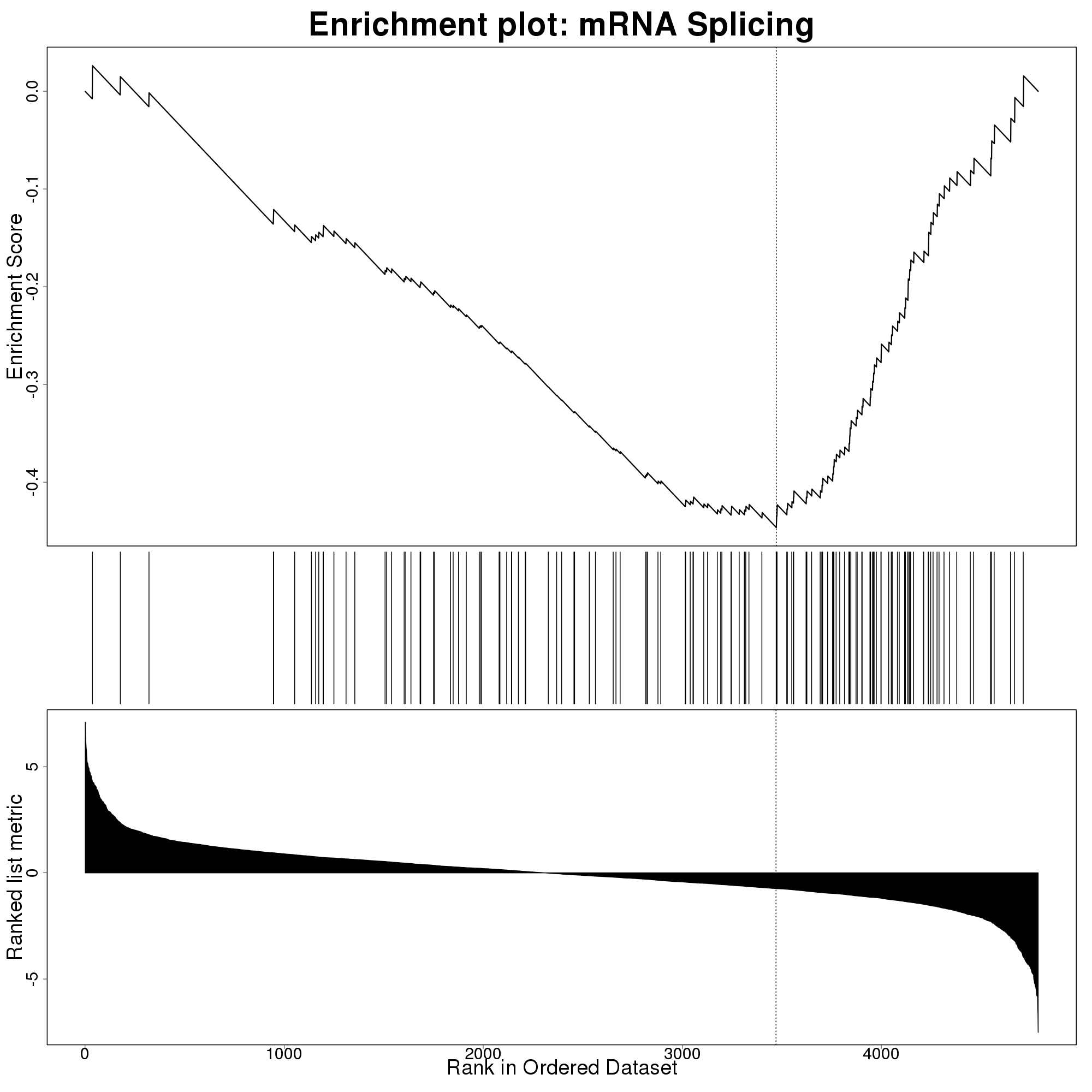

Supplement: Supplementary file 1 [file jcm-10-00407-s001.zip › sup/Supplementary_File_6/GSEA_Webgestalt/GSEA_Pathway_Reactome/Project_wg_result1604400246_GSEA/R_HSA_72172.png]

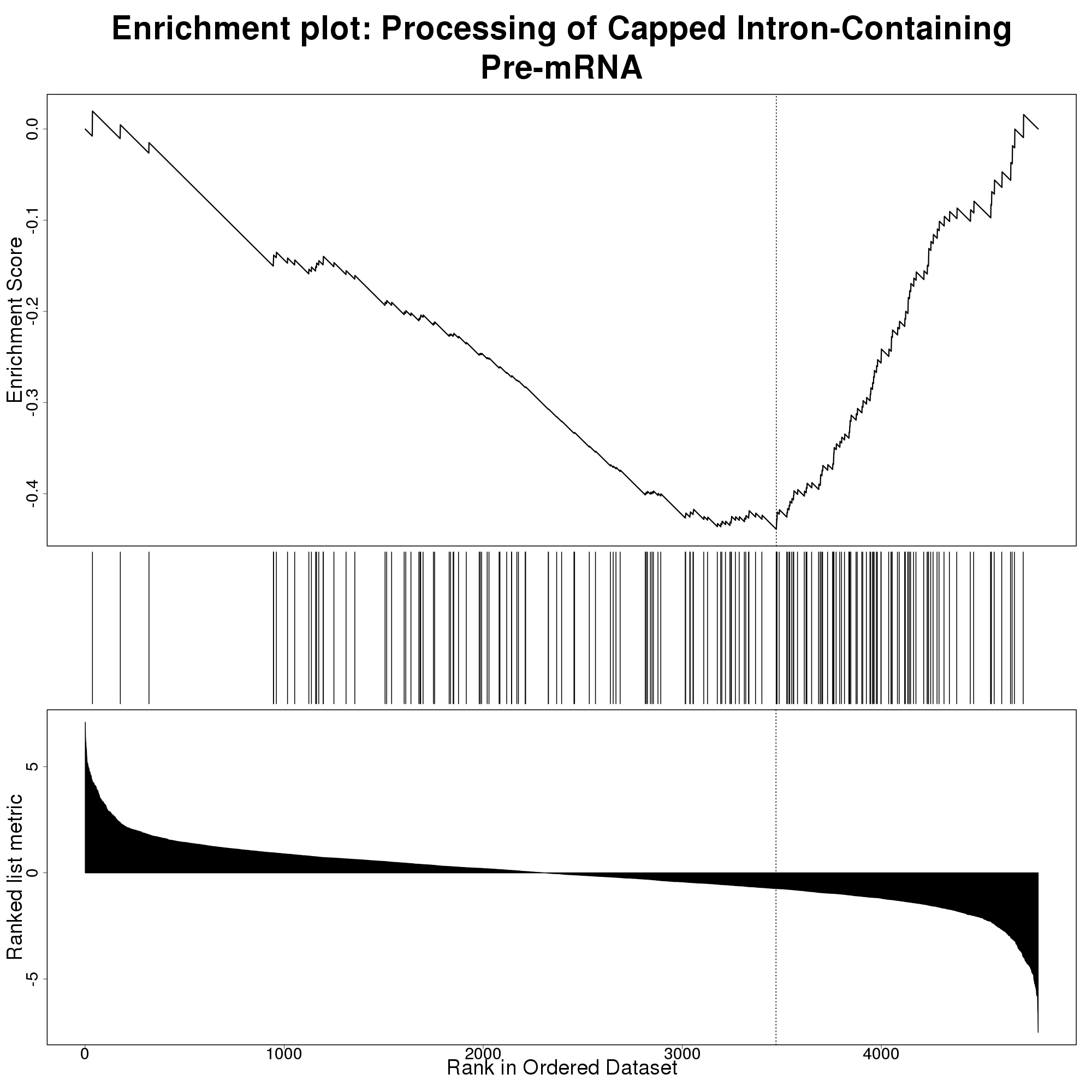

Supplement: Supplementary file 1 [file jcm-10-00407-s001.zip › sup/Supplementary_File_6/GSEA_Webgestalt/GSEA_Pathway_Reactome/Project_wg_result1604400246_GSEA/R_HSA_72203.png]

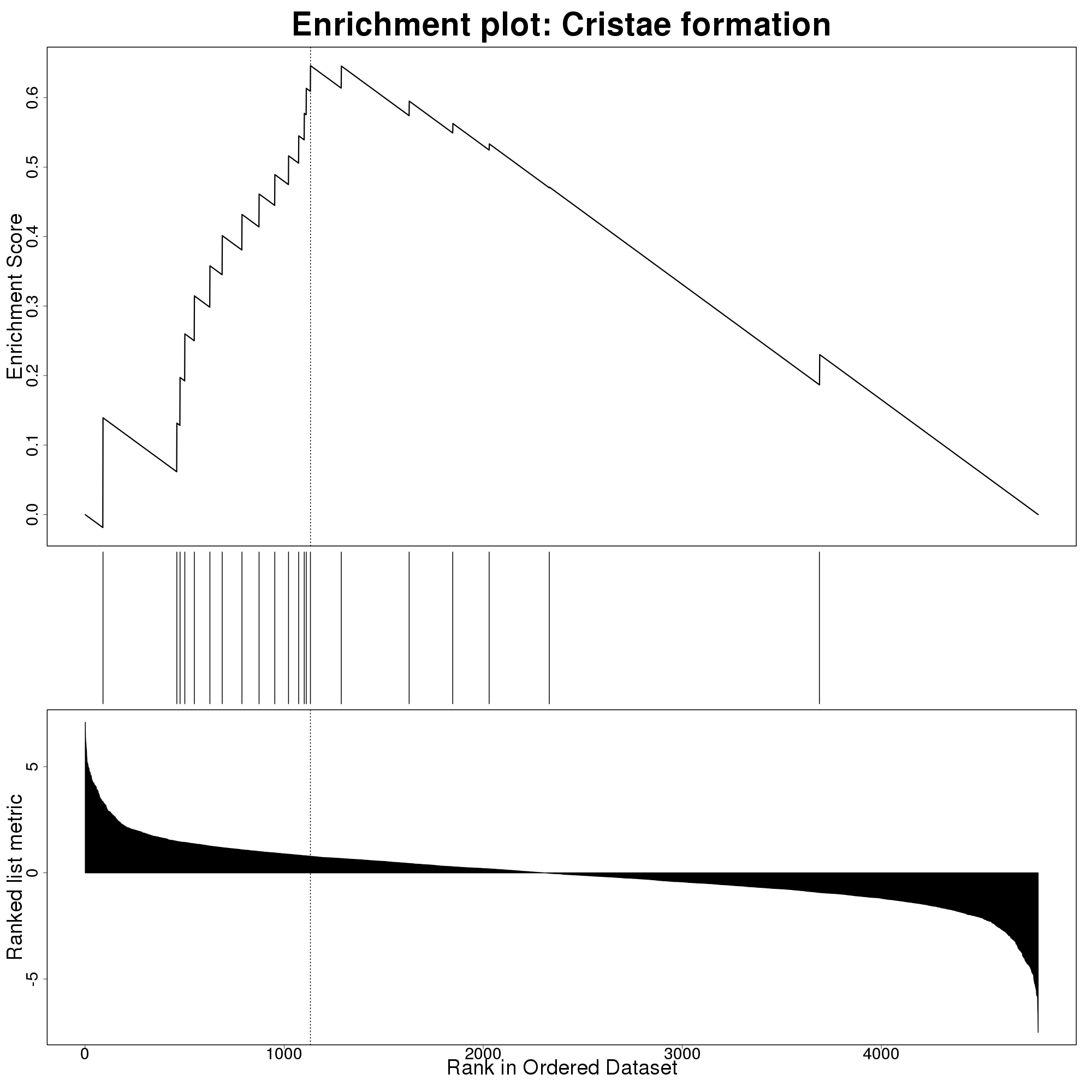

Supplement: Supplementary file 1 [file jcm-10-00407-s001.zip › sup/Supplementary_File_6/GSEA_Webgestalt/GSEA_Pathway_Reactome/Project_wg_result1604400246_GSEA/R_HSA_8949613.png]

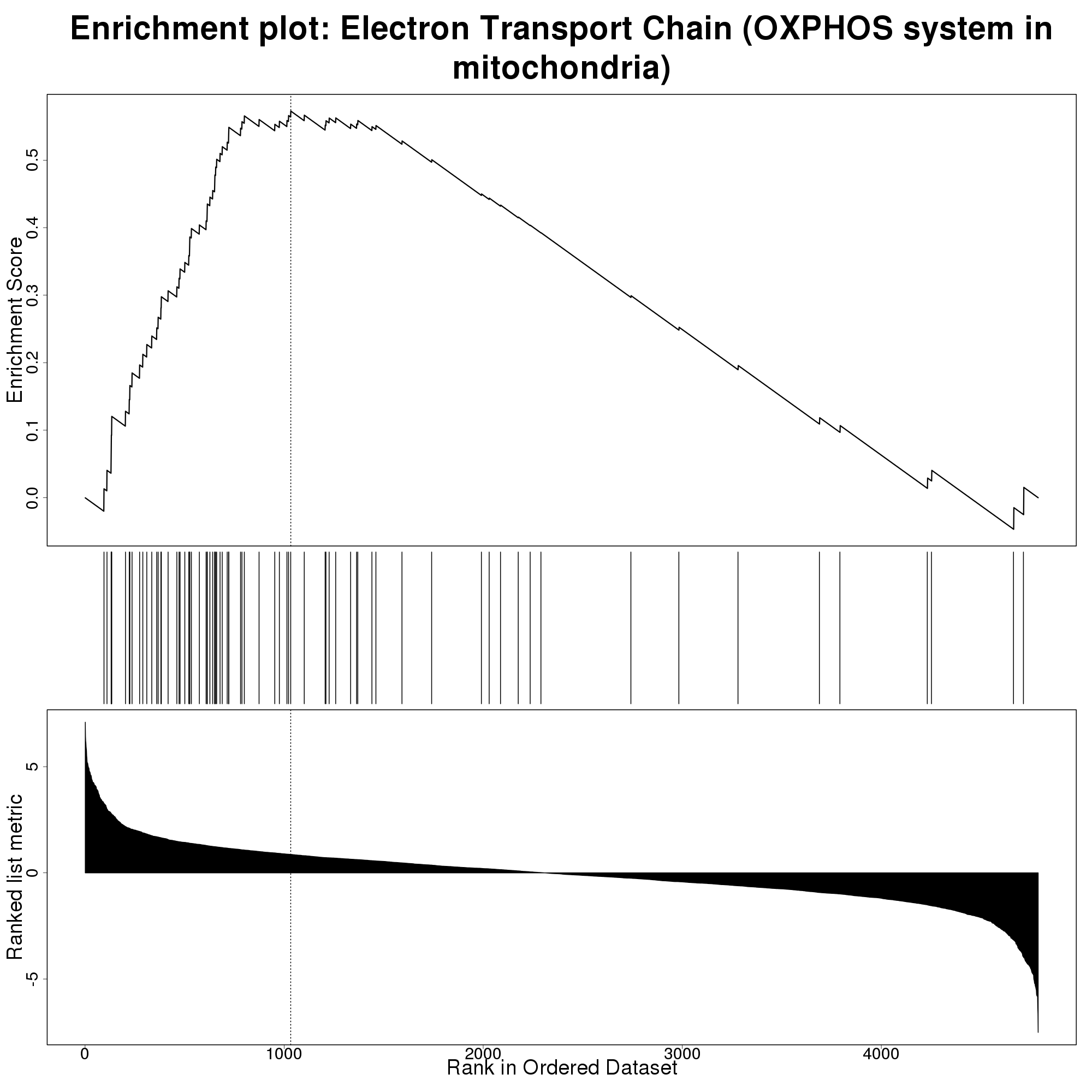

Supplement: Supplementary file 1 [file jcm-10-00407-s001.zip › sup/Supplementary_File_6/GSEA_Webgestalt/GSEA_Wikipathway/Project_wg_result1604400256_GSEA/WP111.png]

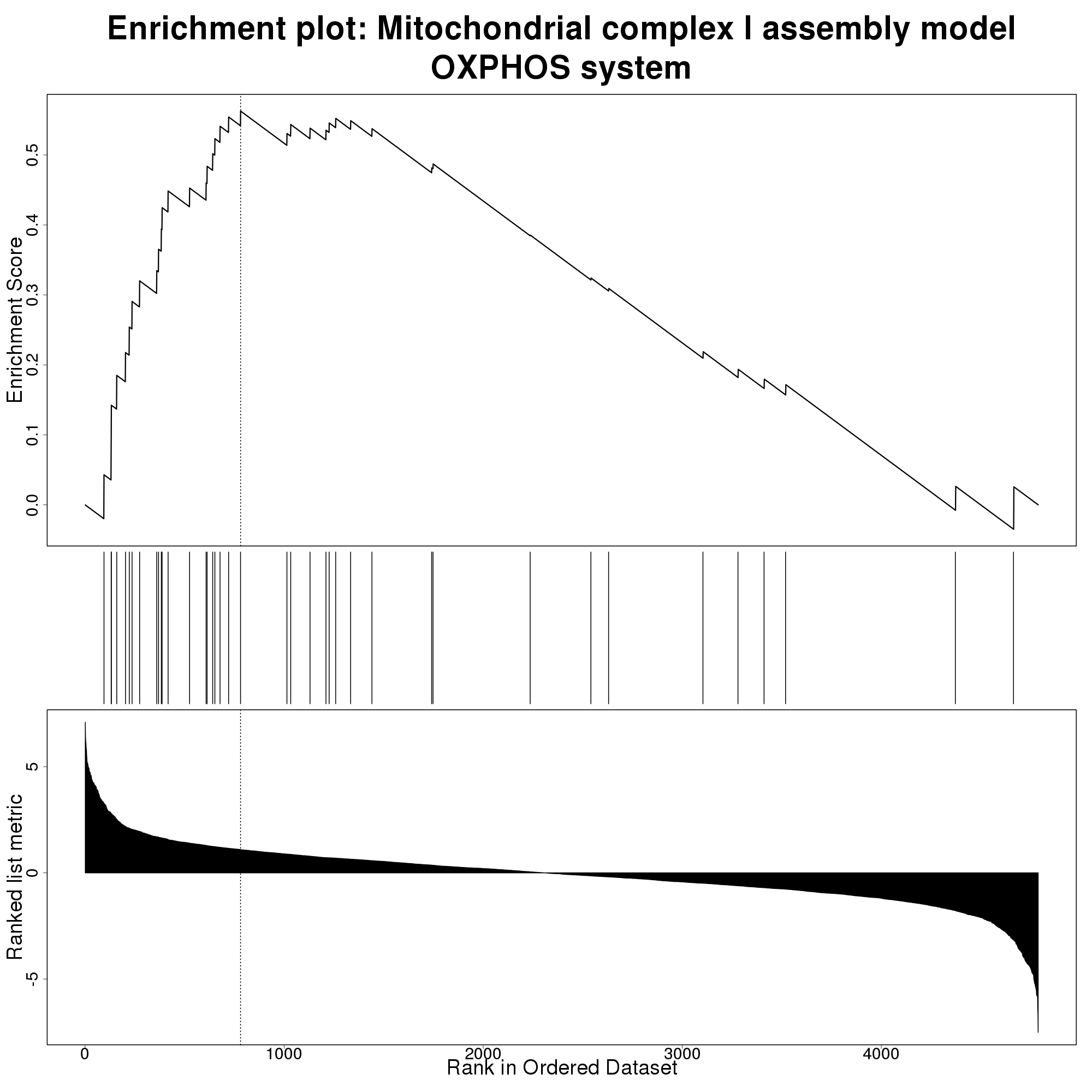

Supplement: Supplementary file 1 [file jcm-10-00407-s001.zip › sup/Supplementary_File_6/GSEA_Webgestalt/GSEA_Wikipathway/Project_wg_result1604400256_GSEA/WP4324.png]

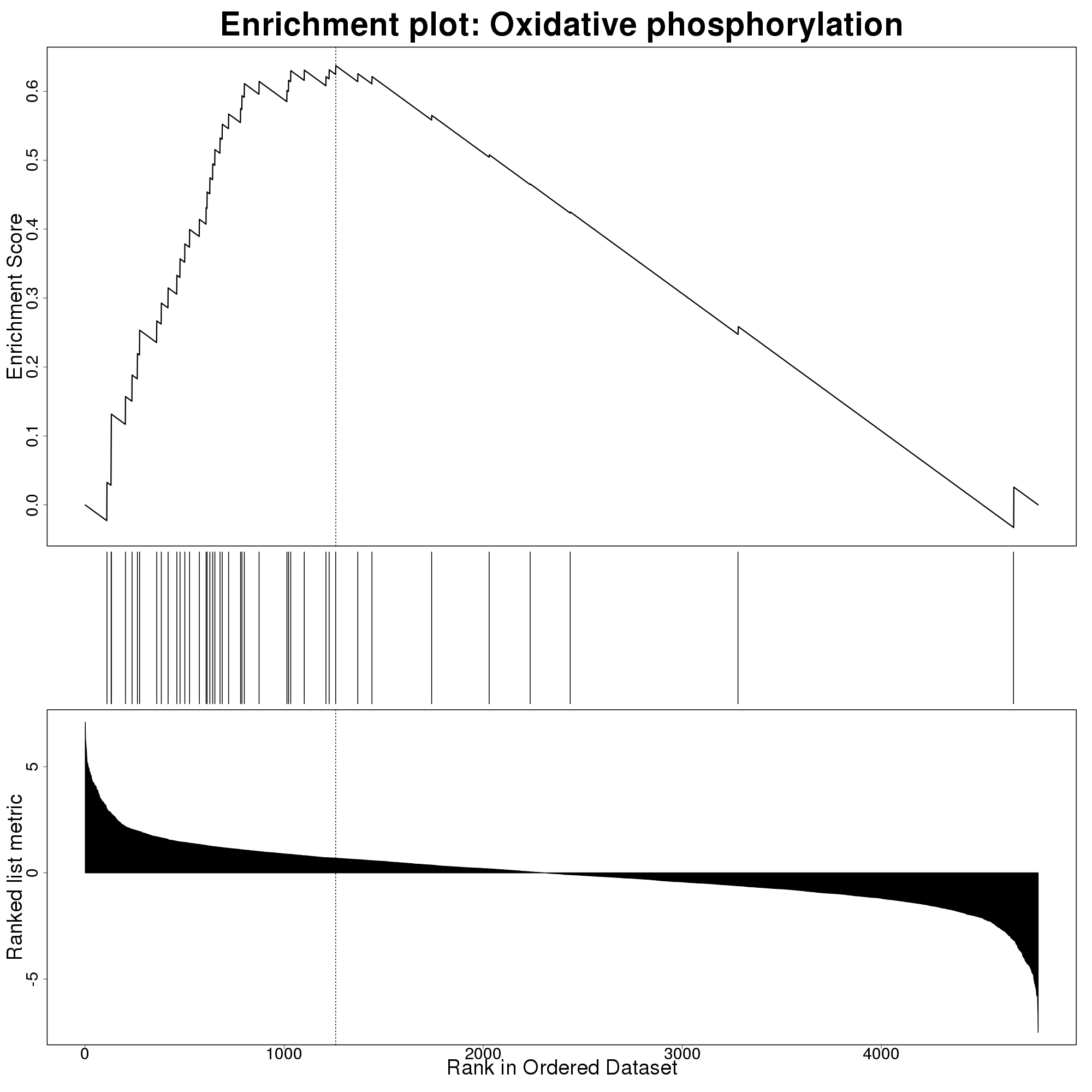

Supplement: Supplementary file 1 [file jcm-10-00407-s001.zip › sup/Supplementary_File_6/GSEA_Webgestalt/GSEA_Wikipathway/Project_wg_result1604400256_GSEA/WP623.png]
